# Empowering Children Through School Re-Entry Activities After the COVID-19 Pandemic

**DOI:** 10.5334/cie.17

**Published:** 2020-05-15

**Authors:** Michele Capurso, John L. Dennis, Luciana Pagano Salmi, Cristina Parrino, Claudia Mazzeschi

**Affiliations:** 1 Università degli Studi di Perugia, IT; 2 Università Cattolica del Sacro Cuore, IT; 3 University of Utah, US; 4 Ebookecm, IT

**Keywords:** Covid-19, coronavirus, children, school re-entry, socio-emotional processing, stress, coping, classroom activity

## Abstract

The isolation related to the COVID-19 pandemic is causing both physical and mental health concerns for children worldwide. When the pandemic is over, schools and kindergartens represent a crucial context that can play an important role in promoting young people’s well-being. This paper presents a school re-entry program aimed at creating an arena where children can process emotions, rediscover interpersonal connections, and develop an awareness of effective coping strategies. For all kindergarten, primary and middle school students, suggestions for evaluating the effectiveness of the program based on its educational and psycho-social components are given. School is an ideal setting to deliver these activities to children as it represents return to their daily routine. Schools also provide equal access to resources and reach children belonging to at-risk socio-economic categories and cultural minorities. Two printable activity packs are provided as additional materials for teachers who want to recreate or adapt the presented activities for their own contexts.

The crisis related to the Covid-19 pandemic is causing health and general wellbeing concerns worldwide. Although children appear to be less prone to be infected by the coronavirus than adults (Hong, Wang, Chung, & Chen, 2020), they may be more fragile from a psychological viewpoint (Jiao et al., 2020) and present anxiety, behavioral problems, and fear as a consequence of the isolation. As a result, mental health professionals suggest that specific interventions be carried out during the pandemic (Liu, Bao, Huang, Shi, & Lu, 2020), and have proposed guidelines for improving communication with children while they are required to stay at home with their families (Dalton, Rapa, & Stein 2020). While the proposed interventions focus on the lockdown phase of the pandemic, they will also be applicable when the immediate pandemic is over and children start returning to school and kindergarten – a phase that will play a crucial role in terms of children’s mental wellbeing (Sandoval, 2013).

Intervention guidelines for the educational contexts can be deduced and adapted from the more general school crisis literature (Jimerson, Brock, & Pletcher, 2005; Sandoval, 2013). They are presented here together with a set of worksheets and other training materials specifically created for the COVID-19 pandemic. Teachers, educators, and school psychologists can use these resources to plan a consistent school re-entry program to be run when school restarts at the end of the health crisis.

Related to the present pandemic situation, this article starts out by reviewing childhood stress, coping, and resilience dynamics and goes on to describe how a school can serve as a crucial context where children can make sense of their lived reality through narrative thoughts and recognizing and sharing their emotions. Finally, after a brief overview of crucial school-based intervention principles proven effective in the past, we introduce a multilevel school re-entry program that, while developed for Italian schools, can be implemented in a variety of school settings with children of different ages.

## The COVID-19 Pandemic

On March 11, 2020, the World Health Organization (WHO) declared the COVID-19 pandemic. Infection by a novel coronavirus named SARS-CoV-2 (Severe Acute Respiratory Syndrome Coronavirus-2) can cause a disease named COVID-19 (Coronavirus Disease, 2019; Pedersen & Ho, 2020). The first cases of COVID-19 were observed in December 2019 in the city of Wuhan, China (Chen et al., 2020). Rapidly, the disease spread all over the world, and, as of April 28, 2020, it had reached over three million people (CSSE, 2020).

The spreading of COVID-19 occurs through droplets released by talking, coughing, or sneezing by infected people (WHO, 2020). To contain the pandemic, WHO has recommended a set of personal hygiene procedures and strict measures of social distancing (WHO, 2020). As a consequence, several countries have ordered a general lockdown, including the closing of entire industries, businesses, social and recreational venues, and schools. In Italy, the lockdown started on March 8 in Northern Italy and then gradually extended to the whole country in subsequent days. Although the intensity of some lockdown measures was reduced on May 4, schools in Italy are expected to remain closed until September 2020; however, the situation is constantly developing and changing (GIMBE Foundation, 2020), causing additional uncertainty and anxiety. Such a long physical isolation and interruption of the social life connected to school are a cause for great concern with regard to the level of stress and its consequences on children.

## Stress, Trauma, and Coping in Children

### Quarantine as a Stressor for Children

Because the pandemic is still evolving, there are no definitive results yet, but early evidence indicates that the quarantine related to the COVID-19 pandemic has a negative effect on children’s lives. During recent parent-based interview research in Italy and Spain, for example, Orgilés, Morales, Delvecchio, Mazzeschi, and Espada (2020) collected 1,143 parent surveys. Results showed that 86% of the respondents reported changes in their children’s behavior and expression of emotions during the quarantine, including difficulty concentrating, boredom, irritability, restlessness, loneliness, discomfort, and expression of worries. Similar results have been found in other regions of Italy (Pisano, Galimi, & Cerniglia, 2020) and China (Jiao et al., 2020). It appears, therefore, that the prolonged isolation connected to the pandemic is causing high stress and psychological consequences in children.

### Stress and Its Characteristics in Children

According to one of the earliest researchers in the field, stress is defined as the “non-specific response of the body to any demand for change” (Selye, 1976, p. 137). Adaptation to stress and adversity is key to human development (Compas, Connor-Smith, Saltzman, Thomsen, & Wadsworth, 2001) and an essential function of the human body (Lazarus & Folkman, 1984). That is, when facing a stressor, human beings appraise the relationship between themselves and the environment as either taxing or exceeding their resources and, therefore, endangering their wellbeing (Lazarus & Folkman, 1984). As a consequence, stress is connected to the subjective assessment of one’s ability to respond to stimuli that derive from changes in the environment, the so-called “stressors” (Ammaniti, 2010).

The ability to appraise and respond to stressful situations is moderated by one’s level of cognitive and emotional development (Smith & Carlson, 1997). For children and young adults, the ways by which they understand and react to stressful events depend on their level of exposure, age, gender, psychological functioning, personality, support culture, and environment, including the influence of proximal adults (Compas, 1987). Younger children are accustomed to relying on adults and other influential figures to take care of their primary needs (Shaffer & Kipp, 2013). The same is partially true also for emotional processing: Children process reality by relying on the emotions transmitted by the adults who take care of them (Bowlby, 1969), a process called *emotional contagion* (Coyne et al., 2020; Hatfield et al., 1994).

To understand the reactions of children and adolescents in the face of critical events, it is important to embrace a developmental and dynamic perspective that sees the child as an active, persistently changing subject in a continuous relationship with his or her environment (Battacchi & Bastianoni, 2002; Lerner, Walsh, & Howard, 1998; Lingiardi & McWilliams, 2017). Research on children and adolescents shows that they are more likely than adults to report stressful changes or incidents that affect their family routines or concern their school life (Compas, 1987). For this reason, even for children, the critical factor in defining stress should be how the individual evaluates the event or situation in terms of its implications for his or her wellbeing.

The majority of children tend to present only mild and transitory psychological effects even in response to intense and distressing experiences, and recovery is the norm. However, in some cases, prolonged or unmanaged exposure to stress can lead to psychopathological consequences (e.g., depression, conduct disorders, anxiety disorders, and post-traumatic stress disorder; Danese, Smith, Chitsabesan, & Dubicka, 2020). Therefore, if teachers perceive persistent negative changes in a child’s behavior or emotional expressions, they should discuss them with the family (and with a school psychologist, where available) and together evaluate whether to seek professional psychological help. A clinical intervention can usually diminish the traumatic impact, assist in recovery, and bring the child back to a level of healthy academic, social, and emotional functioning (Theodore, 2016).

### Coping and Resilience in Children

The strategies we employ to manage and adapt to stressful and ever-changing environments and situations are called coping (Lazarus & Folkman, 1984). The perception of stressors and coping responses change throughout our lives and are connected to our appraisal of the situation, the type of problem faced, socio-cultural aspects, and developmental stage (Losoya, Eisenberg, & Fabes, 1998; Skinner & Zimmer-Gembeck, 2007). That is, coping is an active process in which a set of actions, implemented through cognitive, emotional, and behavioral strategies, are put in place to adapt to, change, or manage internal or external stressors (Compas, Jaser, Dunn, & Rodriguez, 2012). In turn, coping may be seen as connected to *resilience*; that is, an individual’s capacity to rediscover a state of stability during or following exposure to adverse experiences that have the potential to disrupt or destroy his or her successful functioning or development (Masten, Herbers, Cutuli, & Lafavor, 2008). Both coping and resilience serve human development because they help the individual to face difficulties (Leipold & Greve, 2009), and can be fostered in childhood if parents and educators promote trust, autonomy, personal identity, and agency (Grotberg, 2003; Spratling, Cavanaugh, Anne Derouin, Mary Dirks, & Searcy, 2019).

The concepts of coping and resilience are of particular relevance for teachers and other educators because, by shifting the focus of attention away from the stressor to individual responses, they open up several areas for an educational intervention. Thus, research has shown that school-based group intervention, including trauma-processing activities, cooperative play, and creative-expressive elements delivered by trained educators, can be effective in reducing the risk of post-traumatic stress disorder and other critical outcomes in children (Tol et al., 2008).

## Making Sense of Reality at School

When formal education restarts after the Covid-19 crisis, many educators will feel the urgent need and desire to “pick up where we left off.” Thus, teachers may be tempted to succumb to an urgency to recover the school program and in the process throw the pupils and themselves into a whirlwind of lectures, explanations, tests and grading, to make up for lost time as fast as possible. While such an attitude is a reasonable response to the prolonged stress and anxiety with which we all lived during the isolation, following it slavishly may cause schools to neglect basic student and teacher needs.

Instead, a decisive role for the restart phase with children and young people will be to help them to build a sense of what has happened and to reconstruct their social and developmental network within the school system (Sandoval, 2013; Theodore, 2016). That is, the crucial educational task of a school resuming its activity after the COVID-19 pandemic is not to merely fulfil the curriculum, but rather to lay the cognitive and social bases of a future that can be rebuilt on (Perticari, 2012). Meeting the curriculum requirements and constructing the future are not incompatible, but only the latter can give meaning to the former.

Before engaging in curriculum-based activities, therefore, schools need to instigate a sense-making process in children by providing an arena where they can process critical events connected to the Covid-19 pandemic at both an emotional and a cognitive level, thereby building up their resilience and minimize the risk of long-lasting trauma.

## The Intervention

### Principles and Methodology for Crisis Management in Schools

The activities presented here are based on a set of established and shared crisis-related intervention principles for educational settings (Jimerson et al., 2005; Johnson & Figley, 1998; Koplewicz & Cloitre, 2006; Theodore, 2016) implemented in different crises all over the world, and have been adapted to fit the current COVID-19 crisis. We hypothesized that the guidelines detailed below would generalize well to the current context.

#### Facilitate classroom discussions about the event

All the proposed activities encourage discussions about the event, either in small groups or with the whole classroom. They all are presented in the form of open-ended prompts, allowing a great deal of free interpretation and personalization for the student, for instance, by encouraging different narrative modes (e.g., story-telling, drawing, writing, fantasy). The activities should always be presented as optional, letting the students freely choose whether to speak about what happened or not. Giving space in the classroom to children’s narratives allows teachers to provide appropriate support and to facilitate re-adjustment of individuals and groups. It also helps to mitigate children’s short- and long-term reactions to prolonged stress and to identify potentially at-risk students for further psychological evaluations by mental health professionals.

Neglecting a crisis experience can have a substantial negative impact on children. For example, the now infamous Chowchilla kidnapping in California in 1976 involved the abduction of a busload of children, who were imprisoned in a buried container in the desert. Three days later the children escaped. Upon their return, the children were told to go home and forget about the incident. Five years later every child suffered from either depression, anxiety, or phobias, and longitudinally some continued to experience problems well into adulthood (Pitcher & Poland, 1992).

#### Be open to feelings and uncertainty

The aim of the classroom discussions is not to transmit a specific content or message, but to empathically listen without judgment or suggestion and to allow children to express different feelings and thoughts and accept them as a normal part of their individuality. Children often feel more comfortable drawing or playing as a means of expressing and dealing with their feelings (Gil, 1991; Webb, 2012). The key message here is that everyone has different ways of dealing with events and stressors, it is OK to be diverse, and if we listen to others, we can learn many things. Accept children’s confusion, uncertainty, ambiguity, as those are all an index of things that they are still processing and need time to fully organize or resolve. In school settings children depend on teachers and staff for emotional support, which lends credibility to school-based crisis intervention efforts (Brock & Jimerson, 2004) and best practices indicate that children need to be linked with peers and teachers through structured activities, where energy is challenged into productive sense-making activities that strengthen social connections (Prinstein et al., 1996).

#### Provide opportunities for children to reconnect socially and with the environment

The goal of this kind of activity is for participants to rediscover a sense of stability; to re-establish the school routine as soon as possible and to facilitate a re-connection with each other and with their environment (see, for example, Activities 2 and 6 in Table 2 and Activity 6 in Table 3). This, in turn, promotes social competence and a positive concept of self and others, while reinforcing resilience and coping capabilities (Interdisciplinary Group on Preventing School and Community Violence, 2013).

#### Shift attention from the stressful memory to an awareness of coping

Although schools have little power over the external crisis-related events, they have ample opportunities to work on the best ways for students to respond to the difficulties posed by COVID-19-related events. Therefore, working on the coping component is a crucial activity that can and should be carried out at school. For this reason, activities that encourage students to share their coping strategies are provided both for preschoolers and older children. The suggested activities will enhance the personal growth of both students and staff through an awareness of adaptive coping strategies for dealing with the troubling situation (Saylor, Belter, & Stokes, 1997).

#### Present facts and provide information

After providing due time for the expression of concerns and feelings, school professionals should provide children with the facts and basic information about the COVID-19 pandemic and management. Always make sure to use the latest official evidence-based sources when presenting facts to prevent the spread of false information. The level and specificity of information shared should be consistent with the child’s age and level of maturity. A helpful way is to use children’s questions and products (narratives, drawings, etc.) to get an idea of their information needs. In these discussions, children can be guided to evaluate what is accurate and what is not as a means of realistically appeasing their fears and concerns. Educators should not mislead children by providing them with a false sense of safety (Sandoval, 2013; Theodore, 2016). For instance, if the school re-entry is connected to the current data about the spread of the pandemic, teachers are not to say, “we will stay together forever and never have to stay at home again.” Instead, they could say, for instance, “we are now back at school, and all adults are working to make this last, but if the coronavirus should return, we will have to take new measures.”

Following these principles, the proposed intervention is comprised of two parts: teacher training and two classroom activity-packs comprising a set of worksheets. As of May 2020, both of them are being distributed free of charge nationwide in Italy.

## Teacher Training

### Participants

The course is directed at Italian teachers working with children and young people. As part of the course, two sets of school activities have been provided: one for students aged 7 to 12 and one for kindergarten pupils. The course has been advertised through the media and published on the Italian National Ministry of Education Training Catalogue for inservice teachers, SOFIA (course id: 42851). Due to the great demand, the course’s eBook has also been made available to the general public (available in Italian only – see Appendix A).

### Environment

The course is delivered through an online learning platform called ebookscuola.com. Ebook Scuola was started in 2018, based on a similar sister company dedicated to online training of medical practitioners established in 2011 (ebookecm.it).

### Educational Strategies

The course is comprised of the following steps:

1. Study of an eBook, which constitutes the course manual (see Table 1 and Appendix A).
2. Execution of a set of classroom-based activities using the activity packs provided (for primary and middle school students, see Appendix B; for kindergarten children, see Appendix C).
3. Completion of a teacher reflective practice report form.
4. Passing a final test at 60% efficiency.

**Table 1:** Synopsis for the Online Teachers Training Course.

Presentation of the program: Back to school to build the future
**Unit 1**
Microorganisms, infectious diseases, and the novel coronavirus infection
1.1 Microorganisms
1.2 Infectious diseases and the transmission pathways of microorganisms
1.3 Preventive measures and immunization
1.4 Therapy for viral infections, bacteria, fungi, and protozoa
1.5 Respiratory infections, the novel coronavirus, and the pandemic
1.6 The indications of the Ministry of Health to counter the infection from the novel coronavirus in Italy
**Unit 2**
Understanding the disease in children
2.1 Two models of understanding the disease in children
2.2 Towards overcoming stage theories
2.3 The competent child and understanding the disease
**Unit 3**
Helping children to reframe stressful experiences at school
3.1 Children: between daily challenges and stress
3.2 Some general definitions
3.3. Emotional reactions of children in the face of stressful events
3.4. The resources of children in the face of stress: resilience, coping strategies, social support, and positive identity
3.5. Feelings of fear and anxiety, other manifestations of stress, and traumatic elements
3.6 Why it is important to offer spaces for a socio-emotional reframing of stressful experiences?
3.7 What can the school do?
**Unit 4**
Additional guidelines for working with kindergarten children
4.1 Secure foundations and attachment in times of distress
4.2 The school as a place for a “secondary attachment”
4.3 How young children “receive” the emotions of adults around them
4.4 Back to play: exploration, freedom, and coping
**Teacher’s worksheet 1**
Reflective practice worksheet to be uploaded as a mandatory requirement for the online teacher courses after running the activities presented in Appendix 1a and 1b. Labelled Appendix B and C in this article.
**Appendix 1a and 1b**
Activities to help pupils to reframe the emotional experiences connected to the coronavirus crisis (different packs are provided for kindergarten and older children).
**Appendix 2**
Energizers: cultivating relationships in the class.
**Video Resources**
Microorganisms and health-protective actions.

Upon completion of the course, inservice teachers receive a certificate that is recognized by the Italian Ministry of Education as part of their required inservice professional training.

### Instructors

The classroom activities were designed by a professor of educational psychology with previous primary school teaching experience. The course manual has been edited by the same professor and by a professor of dynamic psychology, both from the University of Perugia, Italy. The manual’s content was created in collaboration with medical doctors, clinical psychologists, psychotherapists, and teachers. A professional graphic designer designed and assembled the final eBook and all the worksheets to be used in the classroom.

### Program Delivery and Schedule

The course is delivered using the ebookscuola.com platform, an Open Learning Environment (OLE) that enables users to learn where and when they wish and to receive and send written work asynchronously. This allows maximum flexibility in the study of the content even during the pandemic. Teachers can download the course manual in pdf, epub, or mobi format, and can then read it offline using their preferred device or printing it. Once they have studied the manual, they can complete Steps 2–4, as described above, to finish the program.

### Evaluation

At the end of the course, all attendees are administered a course evaluation, a multiple-choice questionnaire aimed at assessing how useful the course has been for their profession, how difficult it was for them, and whether the declared learning outcomes were fulfilled. Because the course is still ongoing, the results will be examined in a future paper.

## The Back-to-School Activity Pack for Children

Given the ongoing COVID-19 pandemic, the activities have not been implemented at the time of this writing, but they will start immediately upon re-opening of schools in Italy. Even if we are not currently able to examine the results, we decided that sharing the activity plan and evaluation methods at this stage could help other schools to plan their own similar school re-entry program. In line with the aforementioned crisis-intervention principles, the steps and objectives of the activities are presented in Tables 2 and 3.

**Table 2:** Overview of the School-Re-Entry Intervention for Preschool Children.

Objectives	Activity and Description	Rationale	Dimensions of the intervention

Freely regain confidence with other schoolmates and with the school’s spaces. Elicit pleasant moments relating to school attendance.	**1. Free play sessions, possibly outdoors** Play has numerous benefits concerning stress (Fiorelli & Russ, 2012). Play allows children to find a natural form of expression, an arena where cognitive and affective processes are put in place. The first activity to do when children re-enter kindergarten is to let them play freely. This will allow them to regain confidence with the school spaces, classroom tools, and others.	Play is a key component of childhood development and can support coping capabilities (Capurso & Ragni, 2016; Gray, 2015)	Social, emotional, creative
Participate in a structured environmental exploration activity to regain confidence in the school’s spaces. Foster children’s awareness of themselves and others concerning their environment.	**2. Guided tour of school spaces** Children are taken on a tour of the different areas in the school. For each room or school-designated space, children are asked to observe and list the different materials that characterize it. Teachers use probing questions and help the children re-organize the ideas and memories they already have. Teachers also help children to reconnect to that space with time references to remember what time during the day those specific spaces are used. Finally, teachers invite children to recall the aim of a particular school area and to share some fun event they were part of and that is connected to that space.	Young children develop a sense of self in time and space through environmental explorations (Hewes, 1982)	Spatial orientation; development of a sense of self in the context
Recognize that different emotions are a natural part of the self. Realize that other children lived through the same type of events connected to the COVID-19 crisis.	**3. How did I feel at home?** Teachers recap the fact that children had to stay at home with their parents because of the coronavirus emergency. They then show a set of feelings cards (see Appendix C) and ask pupils to: – recognize the represented emotion; – tell when, while they had to stay at home, they felt that specific emotion. **Given that there have been reports of increased incidents of domestic violence throughout the pandemic isolation, teachers should be aware of the fact that children may have witnessed or been a victim of violence. As with any other activity connected to family life, teachers should pay particular attention to signs of potential violence-related distress in children. In line with the local protocols and law, teachers should be ready to contact the local school and social authorities in case of signs or evidence that such violence occurred**.	To process the emotions connected to the stressful events, children need to be able to share them with others and with adults within a setting that can provide a sense of protection (Theodore, 2016)	Emotion recognition and emotion processing
Evoke salient adults present at home with the family and share with classmates. Reinforce in children an awareness of the presence in their life of key adult figures that they can rely on.	4. **The important members of my family** Trusted adults in the life of children are key figures for their emotional processing and represent a key factor that helps youngers to adjust to disruptions in their lives. Teachers ask children to name family members that help them in their daily life or whenever they are facing difficulty. Teachers guide children with specific prompts to recall different situations (e.g., fear, daily life routines, homework, play and have fun). After naming their significant family members, pupils are invited to:	Sharing and brainstorming with others allows children to reflect on different ways of coping with events and assess the consequences of different responses (Jimerson et al., 2005; Theodore, 2016)	Emotional and cognitive; family
	1. Draw themselves and one of their family members as the adult is helping them with something. 2. Tell the class what they drew. 3. Tell the class what they were feeling when the family member was helping them. 4. Explain what the family member was doing and what he/she was feeling while helping the child out. During the activity, the teacher outlines that, while the children’s families can have a different configuration, each child can find, within his/her family, one or more adults that can help them when they need to face some problems. To recap the activity, teachers can create a poster showing the different family figures (mothers, fathers, grandparents, siblings, uncles, etc.) and place next to them the child’s name or a mark that identifies them.		
Create a tangible reference to their own imagination.	**5**. Draw a picture of the coronavirus. Children are asked to imagine the coronavirus and to draw it (see Appendix C). This worksheet can also be used to identify what type of mental image the children have of the virus and give teachers perspective on how they understand it. Moreover, this worksheet prepares children for more cognitive work in Unit 7.	Various affective modulation and cognitive tools can be used to help children express their feelings and better understand the facts (Johnson & Figley, 1998; Theodore, 2016)	Concrete and creative
Make positive anticipatory thoughts and reconnect children with their school community.	**6. The story of the district or the school**. The story of the school gives a common background and aims at allowing children to reconnect with the environment and each other. Additionally, the story elicits positive anticipatory thoughts in children towards the future. The story ends with opening up children’s minds to their wishes and gives them a chance to express them and share with others. After reading the story out loud, teachers can run a set of different activities connecting with it. Activity 6.1 – Soap bubbles **Make sure to conduct this activity following the current safety measure as issued by your health care authority**. The child doing the bubbles should be positioned far apart from the others; it would be preferable to do this activity outdoors. *Materials*: Soap bubbles kit, one printed copy of the Story “At the School District” (see Appendix C). *Procedure*: Read the story “At the School District” to the children, then explain that they can wish for something they want to do the next day and then blow it into the wind. Each child is called on to express his/her wish, tell it out loud, and then make a bubble to blow it in the air.	Use metaphor to process the current situation and direct attention to focus on positive thoughts and mental images (Theodore, 2016)	Emotional, creative, positive thoughts
	Activity 6.2 – Represent your wish Children are invited to represent their wish with a small craft or a drawing. If the school has an outdoor area, it is advisable to let the children go outside to collect natural items for their craft. All the representations of the children’s wishes are collected on a poster for display. On the following days, during circle time activities, teachers and children can choose different wishes to make them come true. Children may receive a copy of the story to be taken home and to read within their family, to share and reinforce a sense of the future.		
Use practical strategies to prevent contagion.	**7. Wash your hands!** Handwashing is one of the best ways to protect children from getting sick. Teaching when and how to wash hands is the most important activity to stay healthy. *Procedure*: To introduce the activity, teachers remind the children about the Story “At the School District” (see Appendix C) and about the nasty little dot called coronavirus that jumped secretly from one person to the other. Then ask children if they know the meaning of the word “protect.” Listen to children’s responses and reformulate them to reflect the correct meaning. Then they ask if the children have any ideas on how to protect themselves from the coronavirus. Listen to the children’s answers and underline the importance of handwashing. Then, they introduce the following activities: Activity 7.1 – Let’s get dirty! *Materials*: Eco-friendly glitter, hand sanitizing gel or children’s body oil, a sheet of paper, small plastic toys. *Procedure*: Mix some lotion and glitter in a bowl, then have the children put some of the “germs” on their hands and rub them together. Invite them to touch some toys, the sheet of paper, and observe what happens to the “germs.” Ask them what would happen if they touched their mouth or face now. Finally, ask them what to do to get rid of the “germs” from their hands and try out different suggestions until you get to the handwashing resolution. Have children wash their hands and point out what happens to the “germs.” Activity 7.2 – Make your handwashing poster *Procedure*: Print one copy of Handout 7.2 for each child (see Appendix C). This is a blank page with a small logo that recalls the action of washing hands. Group children in twos and invite them to fill the page with a drawing or other pictures of their own choice. For example, they could put in the contour or the stamp of their own hand or any other subject. When finished, laminate the sheets so they are water-resistant and post them in the bathrooms and other areas of the school to remind children to wash their hands often. If there are too many sheets, you can rotate them.		Behavioral
	Activity 7.3 – Recap how to wash your hands Using the poster provided (see Appendix C), recap with children the different steps of the handwashing procedure. You can then display the poster in the bathrooms and near other sinks to help children remember the correct method of handwashing.		
To understand what germs are and use cognitive resources to understand the basic dynamics of microbiological life.	**8. Videos to know and understand germs** In the weeks following school re-entry, it may be advisable to plan a science lesson about microorganisms. Children can learn about the pandemic by understanding microbial life. For younger students, two videos from Cincinnati Children’s Hospital have been selected to help children understand what germs are and how they spread: How Germs Spread | Explaining the Science for Kids https://www.youtube.com/watch?v=YBGsoimPXZg Stop Germs From Spreading: https://youtu.be/JD85FDlxqCs Once again, be careful not to exaggerate scary messages to prevent an increase in childhood anxiety.	Children can socially improve their understanding of the illness through interaction with peers and interactive school activities (Capurso, Lo Bianco, Cortis, & Rossetti, 2016)	Cognitive

**Table 3:** Overview of the School-Re-Entry Intervention for Primary and Middle School Children.

Objectives	Activity and Description	Rationale	dimensions of the intervention

Recall a salient moment in the time at home with the family and share it with the classmates.	**1. Draw a moment of these days** With relation to the time spent at home due to the COVID-19 pandemic, children are asked to recall and draw a particular moment or event they experienced while confined at home (see Appendix B). The prompt is neutral (it does not say happy or sad moment), so that children are free to choose what they feel needs to be told. The teacher may use the content of the drawing (the type of scene, the emotions of the faces, and the moods of the characters) to get an initial sense of the children’s feelings.	Children need to be able to rethink and share aspects connected to the stressful event to process their emotions (Theodore, 2016)	Social, familiar
Give opportunities for emotional processing of the past stressful situations and reflection on students’ own and others’ coping strategies.	**2. John and Mary’s thoughts** This worksheet operates at an emotional level. It is used to allow children to express thoughts and concerns they may have had during the coronavirus crisis when activities were interrupted and they had to live in isolation with their families. Children are presented with a vignette that says that John and Mary have heard of the coronavirus from their parents and on television and have different thoughts (see Appendix B). Students are invited to write their thoughts on the vignette. This activity uses a “projective” methodology, as children are not asked directly what their feelings are, but instead, they project those feelings to the vignette’s characters. The results can be shared with other schoolmates. During this activity, children may start to talk naturally about themselves. If this happens, they should be allowed to do it and let the conversation flow freely. Activity 3 must follow immediately after Activity 2.	Sharing and brainstorming with others allows children to reflect on different ways of coping with events and assess the consequences of different responses (Jimerson et al., 2005; Theodore, 2016)	Emotional
	**3. When I’m worried** After talking about the vignette’s characters, children can now shift their attention to themselves. They are asked to list their common worries (not necessarily linked to the COVID-19 pandemic), but most important, they are redirected to think about their coping strategies and about the helping relationships they can rely on (see Appendix B). In terms of coping, these last two aspects are more important than fear or worrying. A sense of distress can be handled better when children perceive they have a set of resources that they can use to face the situation. So, when sharing the results of the activity, always point out the many strategies children will tell about. You may even set up a class poster to recap all the useful strategies and people children come up with. This is a crucial exercise because it moves the focus of the child’s mind from the problem to the coping strategy, so it is worth allowing enough time to it in the class. **Given that there have been reports of increased incidents of domestic violence throughout the pandemic isolation, teachers should be aware of the fact that children may have witnessed or been victim of violence. As with any other activity connected to family life, teachers should pay particular attention to recognizing signs of potential violence-related distress in children. In line with the local protocols and law, teachers should be ready to contact the local school and social authorities in case of signs or evidence that such violence occurred**.		Cognitive-emotional
Create a tangible reference to their own imagination and use logical thought to recall useful strategies to prevent contagion.	**4. Draw the coronavirus** This activity works on an imaginative and cognitive level. Children are asked to imagine the coronavirus and to draw it (see Appendix B). This worksheet can also be used to identify what type of mental image the children have of the virus and give teachers insight into how the students understand it. Moreover, the worksheet prepares children to more cognitive work with Worksheet 5 and Unit 7 (see Appendix B).	Various affective modulation and cognitive tools can be used to help children express their feelings and better understand the facts (Johnson & Figley, 1998; Theodore, 2016)	Imaginative
	**5. What a forgetful guy!** Children are asked to recall three key recommendations to prevent contagion (see Appendix B). This worksheet is used to verify if and how children have internalized medical advice heard on TV. Teachers can use children’s comments to reinforce positive behaviors and correct any incorrect statements. When commenting on healthy behaviors, teachers should always refer to updated governmental official recommendations. It is important to find a correct balance between recommended healthy behavior (e.g., hand washing, cleaning of personal devices) and the concept that microorganisms are a natural part of our environment and that many of them play a key role in sustaining human life along with the rest of the planet. Embracing correct behavior must not become an obsession with any microorganism.		Cognitive
Make positive anticipatory thoughts and reconnect children with their school community.	**6. Back to school again** This activity serves to create positive anticipatory thought and to reconnect the students with their schoolmates (see Appendix B). Now that they are back in school, children can go back to thinking about their relationships with others and all the activities they can do together in the following weeks.	Direct attention to focus on positive thoughts and mental images (Theodore, 2016);	Socio-relational
Know germs and use cognitive resources to understand the basic dynamics of microbiological life.	**7. Videos to know and understand germs** In the weeks following school re-entry, it may be advisable to plan a science lesson about microorganisms. Children can learn from the pandemic by studying and understanding microbial life. For younger students, two videos from Cincinnati Children’s Hospital Medical Centre have been selected to help them understand what germs are and how they spread; How Germs Spread | Explaining the Science for Kids https://www.youtube.com/watch?v=YBGsoimPXZg Stop Germs from Spreading https://youtu.be/JD85FDlxqCs Once again, be careful to prevent increasing children’s anxiety by avoiding exaggeration of scary messages.	Children can socially improve their illness understanding thorough peers and school interactive activities (Capurso et al., 2016)	Cognitive

### Participants

The activities presented here were configured for children aged 3 to 12 years old who are able to execute simple tasks efficiently.

### The Context

The activities should be implemented as soon as school restarts after the COVID-19 crisis. They are meant to prepare students for subsequent cognitive and curriculum-based learning in school because the goal is to recreate a classroom connection and a safe socio-emotional environment. The activities proposed here are meant to be implemented in the classroom. Students are required to produce individual work that they will then share with a classmate.

### Learning Objectives

The activities pursue the following learning outcomes:

- Allow children proper space and time to reconnect with each other by strengthening previous relationships and establishing new ones.
- Allow children to feel safe and secure at school.
- Allow children to process stressful events at school and develop resilience, coping strategies, and a positive self-image.
- Understand microorganisms and know basic infection-prevention behaviors.

### Materials

Children’s activities are delivered through two sets of worksheets for kindergarten and older children, respectively. For a detailed overview of the kindergarten activities, see Table 2 and Appendix C; for primary and middle school, see Table 3 and Appendix B.

## Implementation of the Intervention

### Educational Strategies

The activities can be delivered to the class in the form of small workshop sessions using the activity sheets provided (Appendices B and C). While the initial phase of the activity is always individual, children should then be encouraged to compare and discuss the results in pairs or small groups. As noted in the introductory part of this article, narrative thoughts should be encouraged as a means of constructing a sense of the children’s reality. According to Bruner (1986), narrative thought is a key instrument that humans use to make sense of reality. Creating a narrative is not a passive action; on the contrary, Bruner claimed that through narratives, humans explore hypotheses and test different solutions, which, in turn, helps them actively construct their worlds. Besides, several of the presented activities aim at facilitating the expression of feelings. Each student should be allowed to share, but no one should be forced – always remind students of their right to pass on their turn. Listening uncritically to others is already a form of participation that should be positively appraised and students should be reminded that feelings are neither right nor wrong; they just are a natural part of ourselves.

### Instructors

The activities can be carried out by any educator eligible to teach the target for each age range (3–12). Other teachers who work in the same class should be made aware of the activity and its educational objectives. Teachers with counseling experience are best equipped to present the program, but any other teacher can administer it, provided they understand that the main aim of the activities is to create opportunities for communication and thoughts to share, and not to deliver specific content. Educators working in another context (e.g., community centers, after-school care centers, summer camps) can also implement the activity, provided this is included in their institution’s educational plan and that instructors remain readily available to children in the days following the activities. Under no circumstance should the activity be run occasionally or partially without a clear educational and delivery plan. Supervision by a school or educational psychologist, where available, is highly recommended.

### Program Delivery and Schedule

The program should be delivered to the class face-to-face on the days immediately following school re-entry. The whole program lasts for three to five hours, depending on children’s age and inclination to share their products and thoughts. For primary and middle school classes, Activities 2 and 3 from Appendix B must be run one after the other on the same day, as they represent the crucial transition from stressful thoughts to coping strategies and helping relationships. To be able to follow each child in primary school, an optimal student-to-teacher ratio when delivering the activities would be 12–15 students per teacher or class assistant.

## Evaluation

For children in primary and middle schools, the program may be evaluated based on both its educational and psycho-social components and value.

### Educational Evaluation

For primary and middle school students, a brief, self-report questionnaire has been provided as part of Appendix B. Children complete the questionnaire retrospectively; the items measure on a five-item Likert scale students’ perception and like of the activities, their involvement, and how they feel the activities helped them to learn about coping. Two open-ended questions allow students to indicate the activity that they most appreciated and to suggest improvements for other possible future programs. Children with limited writing skills or with a learning disability may complete the questionnaire by proxy.

### Evaluation of the Psycho-Social Effects of the Program

In primary and middle school, evaluation of the psycho-social benefits of the program may be carried out using two validated tools within a quasi-experimental research design under the supervision of a school psychologist. Given that many components of a crisis impact adults’ and children’s anxiety and negative affect (Brock et al., 2016; Weems et al., 2007), we anticipate a positive effect of the program on these psychological traits. Participants’ feelings and positive and negative emotional state should be measured at two time points, before starting the activities and after completing them. Comparing pre- and post-program states at an individual level gives an idea of the effectiveness of the program for the participants, and comparing the whole classroom results at two time points gives an idea of how the program worked at the class level. For this purpose, two validated psychological tools are suggested.

#### State-Trait Anxiety Inventory for Children (How I Feel Questionnaire), STAIC (Spielberger & Edwards, 1973)

The STAIC is a measure of anxiety in primary school children. The A-State form is used here, given the short duration of the presented activities. This form consists of a 20-item scale that measures state anxiety in children 8–14 years of age. The scale assesses the shorter-term state anxiety that is commonly experienced in specific situations. In our case, the anxiety-provoking situation is the return to school after the long crisis caused by the COVID-19 pandemic. It prompts the child to rate 20 statements from “hardly ever true” to “often true.”

#### Positive Affect and Negative Affect Schedule-Child Form (PANAS-C) State form (Watson, Clark, & Tellegen, 1988)

This 27-item, self-report questionnaire is used to measure respondents’ emotions at present. Items are grouped into two separate subscales measuring positive and negative affect, respectively. The respondent is asked to read several words that describe present feelings and emotions and enter a number that corresponds to the value on a 5-item, Likert-type scale. The response scale ranges from “not much or not at all,” with a value of 1, to “a lot,” with a value of 5. The Italian validated version of the scale is comprised of 22 items (Ciucci et al., 2017). The scale is publicly available (see https://www.phenxtoolkit.org/protocols/view/180502 for further information).

#### Effect in Play Scale-Preschool version (APS-P; Kaugars & Russ, 2009)

For children in kindergarten, it may be possible to evaluate their emotional state at different times through play. A trained professional can use this standardized instrument aimed at measuring cognitive (organization, elaboration, imagination, comfort) as well as affective components (the frequency and variety of affective themes and positive versus negative affective themes) of a video-recorded pretend play session. The APS-P has been validated, and is readily available in Italy (Mazzeschi et al., 2016).

Finally, after the activity will be delivered, it will be possible to perform some qualitative and descriptive research based on a content analysis of the children’s products and drawings (Angell, Alexander, & Hunt, 2014; Bombi, Pinto, & Cannoni, 2007).

## Conclusion

The set of activities presented here have been created in Italy and made available to all teachers as part of their required inservice training. Given the current state of the COVID-19 pandemic and the consequent lockdown in the country, we cannot present any results of the school program yet, but we have outlined our agenda for its evaluation and future research.

We propose that once children are back at school, their need to process the events connected with the prolonged isolation caused by the COVID-19 pandemic will be paramount, and it would be a serious mistake to neglect it. We hypothesize that intervening in the classroom setting offers several advantages, including:

1. It provides equal access to resources for everyone and reaches students belonging to more at-risk socio-economic categories and cultural minorities.
2. The school represents an ordinary setting and gives pupils a sense of normality, along with a return to their normal life.
3. School can play a unique role in helping children to analyze past events and identify key coping strategies.

This program focuses on children. In addition to the issues described above, there is another variable that can impact students at school re-entry, namely teachers’ stress. Analyzing this factor and how to support teachers goes behind the purpose of this paper, but it is a topic that deserves attention.

It should be noted that our program is geared to the Italian context. Its adaptation to different social and cultural settings may require changing some of the proposed activities, or connecting them to habits, events, rituals that may be part of the local culture. One crucial aspect will be adjusting the program presentation to different types of classrooms and their configurations following the Covid-19 pandemic. For instance, in some countries return to school may be optional for some time, or classes may be split into smaller groups, meeting at different times, or use a blended methodology where part of the class attends from home. Teachers are invited to adjust the program accordingly, keeping in mind its core idea: Any adversity that humans face also brings developmental opportunities. If we have a chance to think it over and discuss it with our peers, we can learn from it and become more complex and adaptable individuals, and find ourselves healthier and stronger than before.

## Additional Files

The additional files for this article can be found as follows:

10.5334/cie.17.s1 Appendix A. Training manual for all school teachers (in Italian; Capurso & Mazzeschi, 2020).

10.5334/cie.17.s2 Appendix B. Activity pack for primary and middle school students.

10.5334/cie.17.s3 Appendix C. Activity pack for kindergarten pupils.
