# Supplementary material for: Empowering Children Through School Re-Entry Activities After the COVID-19 Pandemic
Source: Contin Educ. 2020 May 15;1(1):64–82. doi: 10.5334/cie.17 (PMC11104315; doi:10.5334/cie.17)
Supplement: Appendix A. — Training manual for all school teachers (in Italian; Capurso & Mazzeschi, 2020). [file cie-1-1-17-s1.zip › Accogliere i bambini in classe dopo emergenza coronavirus/Ebook/Accogliere_bambini_coronavirus.pdf]

Accogliere i bambini in classe dopo l'emergenza coronavirus

Come fornire contenimento emotivo e informazioni corrette

A cura di Michele Capurso e Claudia Mazzeschi

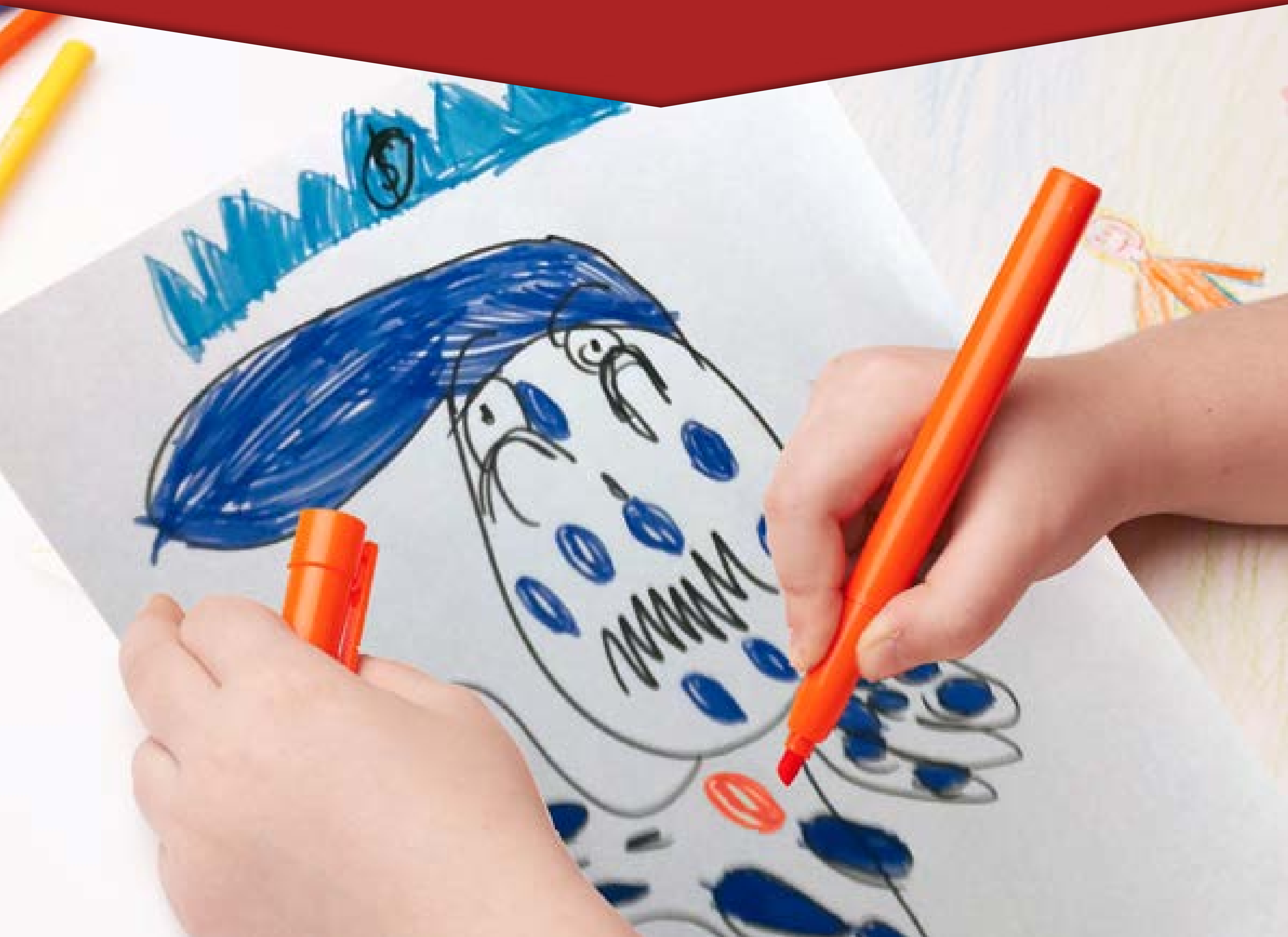

Accogliere bambini in classe dopo l'emergenza coronavirus

Come fornire contenimento emotivo e informazioni corrette

a cura di Michele Capurso e Claudia Mazzeschi

Con contributi di

Cristina Parrino, Michele Capurso, Giulia Cenci,

Giulia Gizzi, Livia Buratta, Elisa Delvecchio, Claudia Mazzeschi

ebookscuola.com

Disegno in copertina di Viola Verona (6 anni)

Questo ebook fa parte di un corso riconosciuto dal MIUR in base alla Direttiva 170/2016 solo ed esclusivamente attraverso registrazione ed iscrizione al seguente link:
https://www.ebookscuola.com/corsi-per-insegnanti/rientro-a-scuola-coronavirus-24.html

Questo ebook è rilasciato con licenza Creative Common BY-NC.
Sei pertanto libero di **condividere** quest'opera — riprodurre, distribuire, comunicare al pubblico, esporre in pubblico, rappresentare, eseguire e recitare questo materiale con qualsiasi mezzo e formato.

ISBN: 9788831253093

2020 by ebookscuola.com
Bookia srl, Piazza Deffenu n. 12, 09125 Cagliari

Indice

Presentazione dell'opera: Tornare a scuola per costruire il futuro 7

Capitolo 1
I microrganismi, le malattie infettive e l'infezione da nuovo coronavirus 12

Scopo del capitolo 12

1.1 I microrganismi: definizione e classificazione 12
1.1.1 I virus 13
1.1.2 I batteri 14
1.1.3 I funghi 14
1.1.4 I protozoi 15

1.2 Le malattie infettive e le vie di trasmissione dei microrganismi 15
1.2.1 Le malattie infettive 15
1.2.2 La trasmissione dei microrganismi 17

1.3 Le misure di prevenzione e l'immunizzazione 19
1.3.1 Le misure per ridurre l'esposizione a microrganismi patogeni 20
1.3.2 La vaccinazione e la sieroprofilassi 20

1.4 La terapia delle infezioni da virus, batteri, funghi e protozoi 21
1.4.1 La terapia antivirale 21
1.4.2 La terapia antibatterica 21
1.4.3 La terapia antifungina 22
1.4.4 La terapia antiprotozoaria 22

1.5 Le infezioni respiratorie, il nuovo coronavirus e la pandemia 22
1.5.1 Le infezioni respiratorie 22
1.5.2 I coronavirus e il nuovo coronavirus SARS-COV2 24
1.5.3 La pandemia 28

1.6 Le indicazioni del Ministero della Salute per contrastare l'infezione da nuovo coronavirus in Italia 29

1.7 Conclusioni 32

1.8 Dizionario medico 33

Bibliografia del capitolo 34

Capitolo 2
La comprensione della malattia nel bambino 36

2.1 Due modelli nella comprensione della malattia nel bambino 37

2.1.1 Lo sviluppo mentale e la comprensione della malattia secondo il modello di Piaget 38

2.1.2 Alcuni esempi delle spiegazioni date dai bambini 41

2.1.3 Le caratteristiche principali della comprensione della malattia secondo il modello piagetiano 46

2.2 Verso il superamento delle teorie stadiali 48

2.2.1 Il Socio-costruttivismo 49

2.2.2 La Zona di sviluppo prossimale 50

2.2.3 Lo Scaffolding 50

2.2.4 Il Pensiero narrativo 51

2.2.5 La costruzione sociale di significati nel sistema bioecologico 52

2.2.6 Esempi di costruzione sociale e di narrazione connessi alla malattia nei bambini 52

2.3 Il bambino competente e la comprensione della malattia 57

2.4 Cosa possiamo concludere 61

Bibliografia del capitolo 62

Capitolo 3
Aiutare bambini e ragazzi a rielaborare vissuti stressogeni a scuola 67

3.1 Bambini e ragazzi tra sfide quotidiane e stress 67

3.2 Alcune definizioni generali 68

3.2.1 Lo stress 68

3.2.2 Lo stress in età evolutiva 70

3.2.3 La funzione dello stress in età evolutiva 70

3.2.4 Il ruolo specifico della famiglia 73

3.3. Reazioni emotive dei bambini di fronte ad eventi stressanti 74

3.4. Le risorse di bambini e ragazzi di fronte allo stress:
resilienza, strategie di coping, supporto sociale e identità positiva 75
3.5. Sentimenti di paura e ansia, altre manifestazioni di stress
ed elementi traumatici. 78
3.6. Perché è importante offrire spazi per la rielaborazione socio-emotiva
dei vissuti stressogeni 82
3.7. Cosa può fare la scuola? 83

Bibliografia del capitolo 86

Esercitazione 1
Svolgimento dell'unità di apprendimentoper la rielaborazione
socio-emotiva dei vissuti relativi all'emergenza causata dal nuovo coronavirus 92

Appendice 1
Attività per aiutare gli alunni a rielaborare i vissuti emotivi connessi
all'esperienza del Coronavirus 93

Appendice 2
Energisers: coltivare le relazioni nel gruppo classe 101

Risorse video
Microrganismi e azioni di tutela della salute 106

Note sugli autori 107

Tornare a scuola per costruire il futuro

Michele Capurso, Claudia Mazzeschi

Un giorno, forse di primavera, tutto questo finirà.

Camminando per strada non scorgeremo più mascherine azzurre ma torneremo ad incrociare i volti umani degli altri. Quel giorno, più di otto milioni di bambini e ragazzi torneranno a scuola. Li accompagneranno i genitori, o forse la raggiungeranno con dei mezzi pubblici disinfettati e tirati a lucido per l'occasione. Al suono di una campanella entreranno in classe, sommessamente e timidamente, senza sapere bene come festeggiare la ripresa di una ritualità che in altri tempi i più grandi avrebbero commentato con anatemi e improperi. Gli alunni ritroveranno il loro banco, smarriti a causa di una prossimità con l'altro così a lungo avversata dalle raccomandazioni ministeriali. Alle 8.15 suonerà la prima ora e bambini e ragazzi saranno richiamati all'ordine. Finalmente, i lettori di questo testo, gli insegnanti, faranno il loro ingresso in aula. Il docente si siederà in cattedra e per la prima volta, dopo tanti giorni di ansia e isolamento, gli sguardi torneranno ad incrociarsi: non attraverso un occhio elettronico, ma per mezzo di un contatto diretto, umano, da persona a persona. Gli alunni scruteranno l'insegnante con quello sguardo di attesa e quella compostezza un po' innaturale, che chi insegna conosce bene e che riflette l'anticipazione della classe per qualcosa di importante che sta per accadere. Cosa stanno aspettando gli studenti?

Bambini e ragazzi attenderanno che l'insegnante li aiuti a dare un senso a quanto è successo, a ricostruire la propria trama sociale ed evolutiva, a riprendere quel percorso di crescita così improvvisamente interrotto dalla crisi del Covid-19. Come si può riprendere un percorso di crescita comune dopo un evento così catastrofico, planetario, subdolo e pervasivo da aver sconvolto per mesi le vite di miliardi di abitanti del nostro pianeta? Il bisogno e il desiderio di "riprendere da dove eravamo arrivati" saranno molto forti; alcuni docenti potrebbero essere facilmente presi dalla fretta di recuperare il programma e potrebbero essere tentati di gettarsi nel vortice di letture, spiegazioni, interrogazioni per recuperare al meglio il tempo perduto.

Non siamo convinti che questo sia il modo migliore per ricostruire le trame di una comunità scolastica e di un clima di classe così lungamente provati dagli eventi. Il

compito educativo della scuola che riprende dopo l'emergenza del coronavirus non sarà soltanto quello del "programma da realizzare", quanto quello di rigettare le basi cognitive, emotive e relazionali di un *futuro da costruire* (Perticari, 2012). Le due cose non sono inconciliabili, ma soltanto la seconda è capace di dare un senso alla prima. Esiste tutto un sistema di bisogni della psiche umana che debbono essere soddisfatti prima che la nostra mente diventi capace di impegnarsi a fondo in compiti di natura cognitiva (Maslow, 1954/2010). I bisogni di sicurezza, di appartenenza, di socializzazione, di contatto umano, di senso, rappresentano dei prerequisiti fondamentali per la strutturazione di un valido processo educativo (Capurso, 2004), ma proprio questi sono stati messi a dura prova dall'emergenza causata dalla epidemia di Covid-19.

Scopo di questo libro è fornire agli insegnanti di scuola media e primaria una serie di indicazioni utili a ricreare le basi socio-emotive di una comunità di classe che è stata a lungo deteriorata dall'isolamento e dalla crisi pandemica. Per nostra fortuna la letteratura su stress e trauma ci informa del fatto che la mente umana è capace di grandi processi di recupero quando si trova a gestire delle difficoltà. Il processo vitale fondamentale capace di sostenere lo sviluppo umano è l'autopoiesi (Maturana e Varela 2012/1970). Il termine è stato coniato dagli autori negli anni Settanta, per cercare di rispondere alla domanda "cos'è la vita?". È davvero curioso e allo stesso tempo affascinante che due biologi, per rispondere a questa domanda, abbiano utilizzato un termine che rimanda alle parole greche *αὐτός* (autós, se stesso) e *ποίησις* (póiesis, creazione; Mengal & Del Miglio, 2001) e che, in un modo piuttosto libero, potremmo tradurre con "autoproduzione poetica del sé". L'autopoiesi è la proprietà fondamentale della vita, quella di sostenere se stessa per mezzo di scambi di energia, di informazioni e di materiali con l'ambiente, mantenendo però coerenti e organizzate le proprie caratteristiche interne (Ford & Lerner, 1995). Dunque, attraverso l'autopoiesi c'è qualcosa che cambia e si trasforma continuamente (il rapporto tra persona e ambiente) e qualcosa che rimane coerentemente se stesso (la radice dell'identità e la struttura interna del sistema vivente). Il risultato finale è che, in virtù dell'autopoiesi, il sistema vivente non si disperde nelle sue interazioni con l'ambiente, ma mantiene una "identità del tutto", che, per dirla con la Gestalt, è data dalla somma delle sue parti interne e da una loro continua interazione reciproca: "Ogni volta che incontri una rete le cui operazioni producano se stessa come risultato, sei di fronte a un sistema autopoietico. Produce se stesso" (Maturana, in Luisi, 2006). Ma il concetto va oltre, perché implica anche la stessa capacità del soggetto vivente di contribuire a modellare l'ambiente intorno a lui, per favorire i propri processi vitali, che nel caso di bambini e ragazzi sono fatti di gioco, crescita, sviluppo, apprendimento (Bronfenbrenner, 2005; Ford & Lerner, 1995).

Di conseguenza, lo scopo di questo volume può essere riassunto da questa domanda: come può la scuola sostenere i processi di autopoiesi umana (cioè i processi vitali al servizio dello sviluppo integrale della persona) ai tempi della pandemia di Covid-19? Per rispondere a questo quesito il volume è diviso in quattro parti.

Il primo capitolo fornisce le informazioni essenziali relative a quanto sta accadendo in relazione all'epidemia di Covid-19 (COrona VIrus Disease, malattia identificata nel 2019, causata da un nuovo tipo di coronavirus, denominato SARS-COV2). Il testo è stato scritto anche con lo scopo di fornire un riferimento, semplice e rigoroso, per programmare future lezioni di scienze e biologia da svolgere in classe. Il capitolo spiega infatti le caratteristiche di diversi tipi di microrganismi, illustrando come alcuni di essi possano diventare patogeni; gli ultimi paragrafi sono dedicati all'infezione da nuovo coronavirus e descrivono anche le misure di igiene e profilassi raccomandate dal nostro governo. A corredo di questo capitolo, ebookscuola ha curato la traduzione italiana di due brevi animazioni, originariamente realizzate dal Cincinnati Children's Hospital Medical Center e messe a disposizione dei lettori che sono liberi di usarle (si veda: https://www.ebookscuola.com/microrganismi).

Il secondo capitolo invece illustra, sulla base di un approccio evolutivo, in che modo bambini e ragazzi comprendono e vivono la malattia e i concetti ad essa correlati. Anche la comprensione della malattia, come ogni altro aspetto della realtà, viene infatti compresa in modo diverso dai soggetti in via di sviluppo, e appare legata ad una combinazione dinamica delle caratteristiche psicologiche della persona e dei vincoli e possibilità di conoscenza forniti dall'ambiente. È utile quindi che gli insegnanti sappiano mettere in relazione i diversi concetti relativi alla malattia, al contagio e alla cura con le diverse fasi evolutive che stanno attraversando i propri alunni. Il capitolo illustra anche come bambini e ragazzi possano svolgere un ruolo attivo nel costruire nuove forme di comprensione e gestione delle diverse situazioni legate alla malattia, se vengono opportunamente accompagnati per mezzo di attività di confronto e co-costruzione sociale dei significati.

Il terzo capitolo di questo libro entra nel vivo delle dinamiche psichiche dello stress e dell'ansia infantile, per poi spiegare il possibile ruolo che la scuola può assumere al momento della ripresa delle lezioni. Il testo spiega in che modo l'essere umano è capace di proseguire la sua autopoiesi anche quando ci si trova di fronte a situazioni problematiche. Il capitolo illustra quindi i processi legati alla resilienza, cioè alla capacità di tornare ad acquisire forme e modi di vita naturali, precedenti ad un evento stressante che li ha provocati, e delinea le diverse strategie di coping, cioè le strategie con cui le persone riescono effettivamente ad affrontare le situazioni stressanti e i problemi della vita quotidiana.

Il testo contiene poi due appendici: la prima presenta un percorso di rielaborazione socio emotiva da svolgere in classe al momento del rientro. Si tratta di un percorso progettato con attenzione da esperti dell'università di Perugia sulla base delle evidenze scientifiche relative alla rielaborazione di eventi stressanti in bambini e ragazzi di età scolare. Il percorso segue una direzione inversa rispetto all'organizzazione dei primi tre capitoli di questo testo. Si parte, infatti, con la dimensione emotiva, per poi passare alle strategie di coping e alla comprensione della malattia e delle norme igieniche da

seguire. Tale progressione è voluta e segue precise indicazioni metodologiche ed è necessario seguirla con precisione.

L'appendice 2, invece, può essere utilizzata più liberamente nelle settimane successive al rientro e presenta attività di gioco e rilassamento per infondere energia positiva ed entusiasmo alla classe.

Tutte le attività presentate hanno la finalità di rinforzare la comunità scolastica, fornendo occasioni di confronto e condivisione con l'altro, con lo scopo di gettare solide basi per i successivi processi di apprendimento.

* * *

Questo libro viene pubblicato in un momento di grande incertezza. Mentre scriviamo non sappiamo ancora se la scuola riaprirà per terminare l'anno scolastico 2019-2020 o se invece ci si ritroverà tutti a settembre. Questo non cambia il senso del nostro messaggio e degli strumenti che forniamo tramite questo testo: qualunque situazione della vita che ci pone di fronte a dei vincoli, offre allo stesso tempo nuove opportunità di crescita. Crediamo che il compito della scuola possa essere quello di sostenere gli alunni per ottenere il meglio dall'emergenza legata al nuovo coronavirus, aiutandoli a scoprire in se stessi e negli altri risorse e capacità di coping e di adattamento. La scuola può fare tutto questo se sarà capace di riconoscere i loro bisogni emotivi, di socializzazione e di comprensione della realtà come prerequisito essenziale del loro sano sviluppo e del successivo impegno curricolare.

Mentre scriviamo non sappiamo ancora quando sarà il rientro, ma quando sarà (e speriamo presto, ma, ci auguriamo, quando opportuno), sarà importante poterne parlare, anche solo per un po', insieme.

Perugia, 25 marzo 2020

Bibliografia

Bronfenbrenner, U. (2005). The Bioecological Theory of Human development. In U. Bronfenbrenner (Ed.), *Making human beings human: Bioecological perspectives on human development* (pp. 3-15). Thousand Oaks: Sage Publications.

Capurso, M. (2004). *Relazioni educative e apprendimento: modelli e strumenti per una didattica significativa*. Gardolo, Trento: Erickson.

Ford, D. H., & Lerner, R. M. (1995). *Teoria dei sistemi evolutivi: un approccio integrato* (S. Castelli, Trans.): Cortina.

Luisi, P. L. (2006). Autopoiesi e definizione del vivente. Retrieved from http://www.asia.it/adon.pl?act=doc&doc=448

Maslow, A. H. (1954/2010). *Motivazione e personalità*: Armando Editore.

Maturana, H. R., & Varela F. J. (2012/1970). *Autopoiesi e cognizione: la realizzazione del vivente*. Venezia: Marsilio.

Mengal, P., & Del Miglio, C. (Eds.). (2001) Nuovo dizionario di Psicologia. Roma: Borla.

Perticari, P. (2012). *Alla prova dell'inatteso*: Armando.

Capitolo 1

I microrganismi, le malattie infettive e l'infezione da nuovo coronavirus

Cristina Parrino

Scopo del capitolo

Questo capitolo ha lo scopo di fornire informazioni scientifiche sui microrganismi, sulla loro modalità di trasmissione e sulle misure essenziali di igiene e profilassi. Gli ultimi paragrafi del capitolo sono dedicati all'infezione da nuovo coronavirus.

Il capitolo è rivolto a tutti gli insegnanti che vi potranno trovare delle nozioni chiare, essenziali e utili a gestire in modo corretto ed equilibrato la comunicazione con gli alunni nei giorni successivi al rientro in classe.

Gli insegnanti di scienze, invece, potranno usare i contenuti del capitolo come fonte di aggiornamento personale e per creare unità di apprendimento più specifiche, adeguate al proprio programma scolastico. Per questo scopo ebookscuola mette a disposizione anche un video "Conoscere i microrganismi", disponibile all'indirizzo https://www.ebookscuola.com/microrganismi.

1.1 I microrganismi: definizione e classificazione

Nel linguaggio comune si utilizzano i termini **"microrganismi"** e **"microbi"**, entrambi derivati dalla combinazione di parole di origine greca **(**"μικρός", piccolo; "βίος" vita), per indicare piccoli esseri viventi non visibili ad occhio nudo, ma osservabili al microscopio. Generalmente, con il termine **"microbi"** si fa riferimento ai microrganismi in grado di causare malattie. In ambito biologico e medico questi microrganismi sono definiti *microrganismi patogeni* (Treccani, 2020; Lanciotti, 2017). Non tutti i microrganismi causano malattie e alcuni di essi sono, invece, necessari all'uomo per mantenere un ottimale stato di salute, e indispensabili in natura e nei processi produttivi, per esempio, nel settore alimentare, biomedico, veterinario, biotecnologico e industriale (Lanciotti, 2017; Murray, 2018).

Il corpo umano ospita numerosi batteri e alcuni funghi e parassiti, che si trovano sulla pelle, all'interno delle cavità nasali e della bocca, nel tratto intestinale e nelle vie urinarie. L'insieme di questi microrganismi viene definito *microbioma*. La presenza equilibrata di questi microrganismi è di cruciale importanza, non solo per la maturazione del nostro sistema immunitario, ma anche per alcune funzioni del metabolismo, per la digestione dei cibi ingeriti e per la protezione contro i *microrganismi patogeni* (Murray, 2018). Alcune malattie possono essere causate da un disequilibrio nel *microbioma*, dovuto per esempio all'uso inappropriato di *antibiotici*, o da un cambio di sede della normale flora "residente", come può succedere a seguito di un trauma (per esempio con l'ingresso di microrganismi cutanei all'interno dell'addome; Murray, 2018).

Le malattie causate da microrganismi presenti nel corpo umano sono definite *infezioni endogene*, mentre quelle causate da microrganismi presenti nell'ambiente sono chiamate *infezioni esogene* (Murray, 2018).

È possibile suddividere i **microrganismi** in quattro **gruppi principali**, che mostrano un grado crescente di complessità nella propria struttura (Murray, 2018; Berkowitz & Jerris, 2016):

- Virus
- Batteri
- Funghi
- Protozoi

1.1.1 I virus

I virus sono dei microrganismi molto piccoli e con una struttura veramente semplice, che comprende (Murray, 2018; Berkowitz & Jerris, 2016):

- una sorta di "libretto di istruzioni" (materiale genetico costituito da un *acido nucleico*, come DNA o RNA)
- dei "mattoncini" (alcune proteine strutturali)
- un cappuccio "grasso" (un involucro di lipidi), presente solo in alcuni casi.

I vari virus conosciuti sono suddivisi in famiglie in base alle caratteristiche della loro struttura (Murray, 2018). Una delle caratteristiche principali dei virus è che questi microrganismi sono **completamente dipendenti dalle cellule viventi** per poter sopravvivere e moltiplicarsi. Per questo motivo i virus sono definiti ***parassiti endocellulari obbligati*** (Murray, 2018).

I virus sono in grado di:

- fare il loro ingresso all'interno delle cellule viventi
- svestirsi dagli involucri esterni
- sfruttare tutti i macchinari interni delle cellule per produrre e assemblare i ma-

teriali indispensabili per poter dare origine a nuovi virus, cioè i "mattoncini' e i nuovi "libretti di istruzioni"
- fuoriuscire dalle cellule
- propagarsi da una cellula all'altra

La sopravvivenza dei virus nell'ambiente esterno dipende da numerosi fattori tra i quali la presenza del cappuccio grasso e le condizioni ambientali come la temperatura esterna e il grado di umidità (Murray, 2018; Berkowitz & Jerris, 2016).

1.1.2 I batteri

I batteri sono dei microrganismi che presentano una maggiore complessità e sono dotati di (Murray, 2018; Berkowitz & Jerris, 2016):
- due "libretti di istruzioni" (materiale genetico costituito da entrambi gli acidi nucleici DNA e RNA)
- macchinari necessari per produrre nuovi batteri
- una parete cellulare organizzata
- strutture accessorie, presenti solo in alcuni batteri, come flagelli e "peletti" che permettono ai batteri di attaccarsi, muoversi e trasferire il materiale genetico (Figura 1).

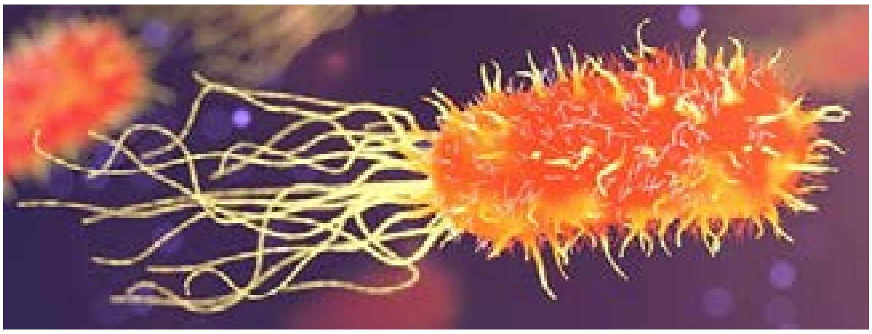

Figura 1. *Batterio con strutture che permettono il movimento (fonte: pixbay.com)*

I batteri sono classificati in base a:
- colore assunto utilizzando dei coloranti specifici (Gram-positivi in blu e Gram-negativi in rosso)
- forma (rotonda, a bastoncino, a spirale)
- capacità di vivere in presenza o assenza di aria (aerobi, anaerobi)
- possibilità di produrre le spore, forme molto resistenti di batteri "addormentati"

1.1.3 I funghi

I funghi o miceti sono organismi che presentano un ulteriore livello di complessità rispetto a virus e batteri e appartengono agli *eucarioti*, cioè organismi con un nucleo ben riconoscibile e con strutture cellulari ben definite (Lanciotti, 2017; Berkowitz & Jerris, 2016). Una caratteristica che li distingue dalle altre cellule eucariote è la presenza

di una parte cellulare esterna rigida, che conferisce ai funghi delle caratteristiche specifiche e che viene sfruttata sia per fare diagnosi di infezioni fungine, che per la terapia contro di essi (Murray, 2018).

I funghi sono suddivisi in (Murray, 2018; Berkowitz & Jerris, 2016):

- lieviti (organismi *unicellulari*)
- muffe (organismi *multicellulari*)
- funghi dimorfici (organismi che esistono in entrambe le forme)

Essi hanno un'ampia varietà di dimensioni, forme e colori e tutti i funghi producono le spore (Lanciotti, 2017). Le spore dei funghi, diversamente da quanto si verifica per i batteri, hanno una funzione nella loro riproduzione (Murray, 2018; Berkowitz & Jerris, 2016). Si ricorda che alcuni funghi sono utilizzati per la produzione di alimenti (per esempio il pane) e farmaci come gli *antibiotici* (vedi paragrafo 1.4;Lanciotti, 2017).

1.1.4 I protozoi

I protozoi, insieme ai vermi e agli artropodi (insetti) rientrano nella categoria dei parassiti, microrganismi e organismi *eucarioti* (Murray, 2018; Berkowitz & Jerris, 2016).

I protozoi, nello specifico, sono microrganismi unicellulari, di varie forme e dimensioni. Gran parte è presente a livello ambientale (terreno, acqua e materia organica in decomposizione; Lanciotti, 2017, Berkowitz & Jerris, 2016). Generalmente i protozoi si moltiplicano all'interno degli ospiti e sono in grado di colonizzare tutti i tessuti e gli organi del corpo umano (Lanciotti, 2017, Berkowitz & Jerris, 2016). Possono, inoltre, produrre le cisti, che rappresentano delle forme in grado di resistere nell'ambiente (Lanciotti, 2017, Berkowitz & Jerris, 2016).

1.2 Le malattie infettive e le vie di trasmissione dei microrganismi

1.2.1 Le malattie infettive

Le malattie infettive rappresentano ancora oggi un problema sanitario rilevante, soprattutto in alcune aree del mondo (World Health Organization, 2001). Come già illustrato nel paragrafo 1.1 non tutti i microrganismi sono responsabili di malattie nell'uomo. I microrganismi che determinano l'insorgenza di malattie sono, infatti, definiti *patogeni* (Murray, 2018).

Il corpo umano è dotato di numerosi sistemi di difesa contro i *microrganismi patogeni*. Tra questi, la prima linea di difesa è costituita da:

- cute (pelle) e *mucose* (rivestimento interno degli organi cavi che comunicano con l'esterno, per esempio la bocca, mucosa orale), con funzione di barriera
- lacrime, saliva, acidità gastrica e urine, con azione antimicrobica o meccanica

I microrganismi, dopo avere superato la prima linea di difesa ed essere entrati in contatto con gli individui, iniziano a riprodursi e innescano una reazione da parte del sistema immunitario dell'uomo (Epicentro, 2020a).

Generalmente il contatto con i microrganismi richiede un certo tempo affinché si possano osservare i segni e i sintomi caratteristici di ogni malattia. Il tempo che trascorre tra l'incontro e l'ingresso del microrganismo nel corpo umano e la comparsa della malattia è definito "**periodo di incubazione**" (Epicentro, 2020a).

La presenza di microrganismi che si riproducono attivamene nell'organismo definisce il quadro di "infezione." Le infezioni possono essere (Epicentro, 2020a):

- **sintomatiche**, cioè accompagnate da segni e sintomi
- **asintomatiche**, cioè senza nessun segno e/o sintomo

Le malattie infettive conosciute sono numerosissime e alcuni esempi sono riassunti in **Tabella 1** (Epicentro, 2020a).

I vari microrganismi (virus, batteri, funghi e protozoi) causano malattie infettive con degli aspetti caratteristici. I virus, per esempio, mostrano una particolare predilezione per specifiche cellule viventi, come per esempio quelle del tratto respiratorio (Murray, 2018). Una singola categoria di batteri, invece, può infettare diversi tessuti, ma un organo o un tessuto specifico possono essere infettati da molti batteri differenti, come per esempio nel caso della polmonite che può essere causata da molti agenti batterici (Murray, 2018). Molti funghi, invece, che non determinano malattie in condizioni di buona salute, possono causare malattie nelle persone con un sistema immunitario molto compromesso (Murray, 2018).

Tabella 1. *Esempi di malattie infettive e microrganismi responsabili (Murray, 2018; Berkowitz & Jerris, 2016; Epicentro, 2020a)*

Malattia infettiva	Microrganismo responsabile	Gruppo di appartenenza del microrganismo
**Varicella**	Virus varicella-zoster	**Virus**
**Morbillo**	Morbillivirus	
**Epatite**	Virus dell'epatite A, B, C, D, E	
**Influenza**	Rinovirus, Coronavirus, Adenovirus	
**Polmonite**	Virus dell'Influenza A, Virus Respiratorio Sinciziale, Coronavirus	

**Faringite**	Streptococco piogeno (gruppo A)	**Batteri**
**Congiuntivite**	Stafilococco Aureo, Streptococco Pneumoniae, Haemophilus aegyptius	
**Otite media**	Streptococco pneumoniae, Haemophilus influenzae, Moraxella catarrhalis	
**Cistite**	Escherichia Coli, Proteo mirabile, Stafilococco saprofitico	
**Polmonite**	Streptococco Pneumoniae, Stafilococco Aureo, Klebsiella pneumoniae	
**Infezioni vaginali**	Candida Albicans	**Funghi**
**Tigna**	Trichophyton (diverse specie)	
**Polmonite**	Candida Albicans, Cryptococco neoformans, Aspergillus fumigatus	
**Leishmaniosi**	Leishmania (diverse specie)	**Protozoi**
**Toxoplasmosi**	Toxoplasma gondii	
**Malaria**	Plasmodi (diverse specie)	

1.2.2 La trasmissione dei microrganismi

Un aspetto molto importante delle malattie infettive è rappresentato dalle dinamiche con cui esse possono essere contagiate, cioè **trasmesse da un individuo all'altro** (Epicentro, 2020a). Non bisogna, però, automaticamente associare il concetto di "contagiosità" a tutte le malattie infettive. Si distinguono, infatti:

- malattie infettive **contagiose**
- malattie infettive **non contagiose**

Le **malattie infettive contagiose** sono causate da microrganismi patogeni che in modo diretto o indiretto sono trasmesse da una persona all'altra (per esempio il morbillo; Epicentro, 2020a). Le **malattie infettive non contagiose** sono dovute al coinvolgimento di altri organismi (*vettori*) in grado di trasportare i *microrganismi patogeni* all'interno del corpo degli individui (per esempio la malaria attraverso la puntura della zanzara e "l'iniezione" del plasmodio nel corpo umano; Epicentro, 2020a).

Le malattie infettive possono essere **trasmesse** da un individuo all'altro attraverso (World Health Organization, 2001):

- il **sangue**, a causa di ferite, introduzione nell'organismo attraverso iniezioni o durante i rapporti sessuali (esempi: HIV, epatite B o C)
- l'**aria**, per l'inalazione di minuscole particelle sospese in aria anche per lunghi periodi
- le **goccioline aero-trasmesse**, per ingresso nelle vie respiratorie di particelle di dimensioni maggiori emesse durante azioni semplici quali parlare, starnutire o tossire, oppure durante procedure di intubazione o indagini diagnostiche (broncoscopia)
- il **contatto** diretto con trasferimento dei microrganismi da uomo a uomo o indiretto con trasferimento dei microrganismi su superfici o oggetti
- i **veicoli**, agenti non animati come cibo, acqua, sangue
- i **vettori**, organismi come gli artropodi, le zanzare e le zecche

Alcuni microrganismi possono essere trasmessi anche attraverso più di una via di trasmissione.

Con il termine ***zoonosi*** sono indicate le malattie infettive trasmesse dagli animali all'uomo (Epicentro, 2020b). I *microrganismi patogeni* possono essere trasmessi direttamente all'uomo, oppure, come si verifica nella maggior parte dei casi, possono contaminare acqua e cibi (Epicentro, 2020b). Alcuni esempi di zoonosi sono rappresentati dall'influenza aviaria, dalla brucellosi, dalla rabbia e dalla leishmaniosi.

Le porte d'ingresso utilizzate dai microrganismi sono rappresentate da ferite cutanee, con interruzione della barriera costituita dalla pelle, dalle *mucose*, dal tratto gastrointestinale, dal tratto respiratorio, dalle vie urinarie (World Health Organization, 2001).

I microrganismi, a loro volta, possono essere riversati nell'ambiente e possono raggiungere gli altri individui attraverso lacrime, secrezioni nasali, liquido spermatico, liquidi presenti a livello polmonare o addominale, feci, urina, sudore, saliva, vomito, latte materno e contatto pelle a pelle (World Health Organization, 2001).

Gli esseri umani, l'acqua e gli oggetti possono costituire dei veri e propri "depositi" per l'immissione dei microrganismi nell'ambiente (World Health Organization, 2001).

L'instaurarsi di una **malattia infettiva** è determinato dalla contemporanea presenza di un ***microrganismo patogeno*** e di **caratteristiche specifiche** dei singoli **individui**, che devono essere sprovvisti di difese naturali innate (sistema immunitario) oppure acquisite (per esempio i vaccini) per uno specifico microrganismo (Epicentro, 2020a).

Alcune condizioni possono, inoltre, rendere, per motivi differenti, alcuni individui

particolarmente a rischio di sviluppare malattia infettiva, e tra questi vi sono (World Health Organization, 2001):

- i neonati
- le persone con diabete mellito
- le persone in condizioni di ridotto funzionamento del sistema immunitario (*immunosoppressione*)
- le persone con malattie cardiopolmonari
- le persone di età avanzata

In base alla **diffusione** nella popolazione le malattie infettive possono manifestarsi in maniera (Epicentro, 2020a; WHO, 2020a):

- sporadica
- epidemica
- endemica
- pandemica

I **casi sporadici** sono quei casi che si osservano in popolazioni in cui la specifica malattia infettiva non è presente in modo stabile (Epicentro, 2020a).

Una malattia infettiva si presenta in maniera **epidemica** quando si osserva un rapido aumento del numero dei casi, oltre le attese, in un breve periodo di tempo (World Health Organization, 2001; Epicentro, 2020a).

Si parlerà di malattia **endemica**, nel caso in cui il *microrganismo patogeno* sia presente nella popolazione in maniera stabile e la malattia si presenti con un numero orientativamente fisso di casi (Epicentro, 2020a).

La diffusione **pandemica** indica, invece, un interessamento globale, esteso a tutto il mondo e che si verifica per la presenza di nuovi *microrganismi patogeni* (per esempio il virus dell'influenza) per i quali la popolazione non ha immunità (WHO, 2020a).

È stato osservato come la maggiore frequenza e rapidità di movimento delle popolazioni rispetto al passato sia una delle cause di diffusione delle malattie infettive e dell'insorgenza di epidemie internazionali e pandemie (Epicentro, 2020a).

1.3 Le misure di prevenzione e l'immunizzazione

Come indicato nel paragrafo 1.2.2, affinché insorga una malattia infettiva sono indispensabili un *microrganismo patogeno* e alcune caratteristiche di predisposizione degli individui. Per tale ragione, per prevenire una malattia infettiva si può agire su due fronti, cioè riducendo (Epicentro, 2020a):

- l'esposizione ai *microrganismi patogeni*
- la predisposizione degli individui a sviluppare la malattia, attraverso *vaccinazione e sieroprofilassi*

1.3.1 Le misure per ridurre l'esposizione a microrganismi patogeni

Procedure semplici come il lavaggio delle mani, l'utilizzo di acqua e cibi non contaminati e l'isolamento fisico dei soggetti con infezione sono delle strategie efficaci per ridurre il rischio di venire a contatto con microrganismi patogeni (World Health Organization, 2001).

L'approccio alle malattie sessualmente trasmesse include attività di educazione sanitaria, la ricerca di soggetti con infezioni asintomatiche e curabili (screening) e la destigmatizzazione della malattia (World Health Organization, 2001).

Per le malattie trasmesse da *vettori* (artropodi, le zanzare e le zecche), invece, è fondamentale la prevenzione delle punture e dei morsi (World Health Organization, 2001).

Per le *zoonosi* gli interventi che aiutano a ridurre l'esposizione a *microrganismi patogeni* sono l'igiene regolare e la vaccinazione degli animali domestici e l'attenzione ad evitare il contatto con gli animali selvatici, che dovrebbero vivere lontani dagli ambienti domestici umani (Epicentro, 2020b).

1.3.2 La vaccinazione e la sieroprofilassi

L'**immunizzazione naturale** a una malattia infettiva si ottiene dopo avere contratto e superato un'infezione (Epicentro, 2020a). La **vaccinazione** è invece una forma di **immunizzazione artificiale**, che protegge l'uomo dai *microrganismi patogeni* senza avere sviluppato la malattia (World Health Organization, 2001; Epicentro, 2020a). La vaccinazione, infatti, mette a contatto virus, batteri o parti di essi con il sistema immunitario e stimola una risposta che renderà l'organismo in grado di reagire prontamente nel caso in cui dovesse entrare a contatto con il *microrganismo patogeno* (Epicentro, 2020a).

La vaccinazione è una forma di **immunizzazione attiva** che stimola la produzione di anticorpi da parte del sistema immunitario e può essere ottenuta somministrando (Epicentro, 2020a)

- *microrganismi patogeni* **vivi attenuati**, cioè con minore capacità di dare segni e sintomi di malattia (per esempio il vaccino per il morbillo)
- *microrganismi patogeni* **inattivati**, resi non in grado di causare la malattia (per esempio il vaccino per l'epatite A)
- **tossine**, prodotti dei microrganismi non in grado di causare la malattia **(**per esempio il vaccino per il tetano)
- **parti** di *microrganismi patogeni* (per esempio il vaccino per lo pneumococco)
- **prodotti virali** geneticamente modificati (per esempio il vaccino per l'epatite B)

L'immunizzazione attiva ha di solito durata a lungo termine. La **vaccinazione protegge** sia il **singolo individuo** che la **collettività**, in quanto la presenza di numerosi soggetti immuni contrasta la diffusione della malattia e protegge anche coloro che non si sono vaccinati o che non possono essere sottoposti a vaccinazione (Epicentro, 2020a). La somministrazione di vaccini vivi attenuati, infatti, è controindicata per i soggetti

con sistema immunitario compromesso (World Health Organization, 2001). È, inoltre, importante ricordare che in passato la vaccinazione ha permesso di eradicare malattie come il vaiolo (Epicentro, 2020a).

La **sieroprofilassi** è, invece, una forma di **immunizzazione passiva** che prevede il trasferimento di anticorpi da donazioni di sangue. Questa forma di immunizzazione ha, a differenza della vaccinazione, una breve durata ma un effetto rapido (Epicentro, 2020a).

1.4 La terapia delle infezioni da virus, batteri, funghi e protozoi

Dopo avere preso in esame le caratteristiche dei vari gruppi di microrganismi nel paragrafo 1.1 e la complessità delle vie di trasmissione delle malattie infettive nel paragrafo 1.2, è più facile comprendere perché ogni *microrganismo patogeno* necessiti di una terapia specifica e perché sia indispensabile seguire scrupolosamente le indicazioni e le prescrizioni dei professionisti sanitari.

Altro elemento aggiuntivo che complica il quadro è rappresentato dalla capacità dei vari microrganismi di sviluppare **resistenza alle terapie antimicrobiche** (Berkowitz & Jerris, 2016; Agenzia Italiana del Farmaco, 2020). Ogni gruppo di microrganismi ha, infatti, sviluppato l'**abilità di sfuggire alle terapie** attraverso meccanismi anche molto differenti (Murray, 2018; Berkowitz & Jerris, 2016). I *microrganismi patogeni* possono essere di per sé resistenti ad una determinata classe di farmaci oppure possono "**acquisirla**" nel tempo. L'acquisizione della capacità di resistere alla terapia può essere indotta anche dall'utilizzo di alcuni farmaci (*antibiotici*), che spingono i *microrganismi patogeni* a cambiare il loro materiale genetico, per riuscire a sopravvivere (Murray, 2018; Berkowitz & Jerris, 2016).

1.4.1 La terapia antivirale

La terapia antivirale agisce bloccando diverse fasi del ciclo di replicazione dei virus, microrganismi completamente dipendenti dalle cellule viventi per sopravvivere e moltiplicarsi (Murray, 2018; Berkowitz & Jerris, 2016).

La terapia antivirale attualmente disponibile è attiva su un **numero limitato di virus** tra cui i virus dell'influenza, diversi herpes virus, i virus dell'epatite B e C e l'HIV (Murray, 2018; Berkowitz & Jerris, 2016).

Negli ultimi anni la maggior parte dei nuovi farmaci antivirali è stato sviluppato per il trattamento dell'AIDS e dell'epatite B e C. In misura minore sono stati messi a punto anche farmaci anche per le infezioni del tratto respiratorio e per le infezioni causate dagli herpesvirus (Murray, 2018).

1.4.2 La terapia antibatterica

La disponibilità della terapia antibatterica è stata una vera e propria rivoluzione

nella lotta alle malattie infettive. Il primo *antibiotico* naturale, identificato da Alexander Fleming, è stato la penicillina (Berkowitz & Jerris, 2016). Con il termine "*antibiotico*" si fa riferimento a sostanze di origine batterica o fungina capaci di contrastare crescita di altri microrganismi (batteri), mentre per le molecole prodotte sinteticamente si preferisce utilizzare il termine di "*chemioterapici antibatterici*" (Murray, 2018; Berkowitz & Jerris, 2016; Agenzia Italiana del Farmaco, 2020). Gli *antibiotici* e i *chemioterapici antibatterici* sono farmaci attivi solo contro i batteri, mentre il loro uso è inopportuno nel caso di malattie infettive causate da virus, funghi o protozoi (Agenzia Italiana del Farmaco, 2020). Sono disponibili numerose famiglie di molecole con azione antibatterica e la loro azione può portare ad un blocco dell'azione dei batteri (azione batteriostatica) o alla morte dei batteri (azione battericida), attraverso danni alla parete o alla membrana cellulare o con l'interferenza nella produzione di materiali e dei "libretti di istruzione" (gli acidi nucleici DNA e RNA) necessari per generare nuovi batteri (Murray, 2018; Berkowitz & Jerris, 2016).

1.4.3 La terapia antifungina

La terapia antifungina è attiva contro i funghi e richiede spesso lunghi periodi di trattamento. Il numero di agenti antifungini disponibili è limitato rispetto alle altre categorie di farmaci e si associa molto spesso a fenomeni di tossicità per chi li assume (Murray, 2018; Berkowitz & Jerris, 2016). I funghi e le cellule di cui è costituito il corpo umano, infatti, sono *eucarioti*. I farmaci anti fungini agiscono principalmente interferendo con la formazione della parete e della membrana dei funghi o con il loro metabolismo (Murray, 2018; Berkowitz & Jerris, 2016).

1.4.4 La terapia antiprotozoaria

La terapia antiprotozoaria è attiva contro i protozoi e, così come la terapia antifungina, presenta problemi di tossicità per chi la assume. Anche i protozoi, infatti, sono *eucarioti* (Murray, 2018; Berkowitz & Jerris, 2016). Alcuni farmaci mostrano maggiore selettività nei confronti dei protozoi. I bersagli della loro azione sono rappresentati dagli acidi nucleici, dai "mattoncini" di cui si costituiscono i protozoi (proteine) o alcuni passaggi specifici del loro metabolismo (Murray, 2018; Berkowitz & Jerris, 2016).

1.5 Le infezioni respiratorie, il nuovo coronavirus e la pandemia

1.5.1 Le infezioni respiratorie

Molti virus differenti sono responsabili nell'uomo di infezioni delle vie respiratorie (**Tabella 1**). Le infezioni respiratorie si possono presentare con manifestazioni che variano dai tipici sintomi del "raffreddore comune" fino a forme di gravi polmoniti (Murray, 2018). Le diverse infezioni respiratorie virali si osservano con maggiore frequenza nei mesi invernali e danno sintomi molto simili, difficilmente distinguibili

eseguendo soltanto una visita medica (Murray, 2018). I **virus** che più comunemente possono causare **infezioni respiratorie** sono (Murray, 2018; Berkowitz & Jerris, 2016):

- Rinovirus
- Coronavirus
- Virus dell'influenza
- Paramyxovirus
- Virus parainfluenzali
- Virus respiratorio sinciziale
- Metapneumovirus umano
- Adenovirus

In generale, in caso di infezioni lievi delle alte vie respiratorie (raffreddore) si suggerisce il riposo e si consiglia di seguire norme igieniche come, per esempio, aerare l'ambiente ed evitare sia le condizioni che favoriscono la secchezza delle vie respiratorie che il fumo di sigaretta (Epicentro, 2020c).

In questi casi, i farmaci prescritti dai medici sono di solito "sintomatici", cioè farmaci che agiscono sui sintomi della malattia ma non sulla causa. In caso di influenza, con sintomi importanti, possono essere utilizzate le terapie antivirali, che devono essere assunte esclusivamente su indicazione medica. È importante, inoltre, sottolineare che la terapia antibatterica va utilizzata solo in caso di sovrapposizione di infezioni batteriche, in aggiunta all'infezione da virus (Epicentro, 2020c). In corso di influenza, per esempio, si possono verificare forme di polmonite di origine batterica (Murray, 2018).

A causa del grande numero e della varietà dei virus che possono causare il raffreddore comune, e per la loro capacità di cambiare nel tempo, non è stato possibile sviluppare un vaccino contro il raffreddore comune. È, invece, disponibile il vaccino per l'influenza, che viene "ridisegnato" ogni anno e rappresenta una valida difesa soprattutto per i bambini e le persone con il sistema immunitario compromesso (Epicentro, 2020c).

Per poter attribuire i segni e i sintomi di una malattia ad uno specifico virus è necessario identificare il virus attraverso alcuni esami diagnostici (Lanciotti, 2017). Poiché i virus devono trovarsi all'interno di cellule viventi per sopravvivere, per far crescere i virus in laboratorio è necessario "coltivarli" utilizzando tessuti viventi.

Nel caso specifico delle **infezioni respiratorie** si possono eseguire (Berkowitz & Jerris, 2016):

- **raccolta di cellule dalla cavità nasale e/o dalla gola** (faringe), utilizzando un bastoncino chiamato tampone (**Figura 2**) e successiva ricerca dei "foglietti di istruzioni" dei virus (DNA o RNA);
- esami del sangue per la **ricerca di anticorpi** diretti contro i virus

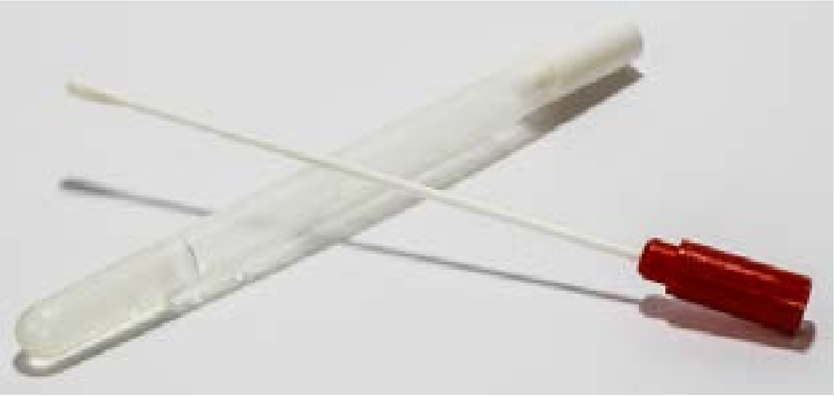

Figura 2. *Tampone per la raccolta di cellule dalla cavità nasale e/o dalla gola (fonte: pixabay.com)*

1.5.2 I coronavirus e il nuovo coronavirus SARS-COV2

I coronavirus sono un gruppo di virus, contenenti l'acido nucleico RNA, che nell'uomo causano generalmente infezioni respiratorie di lieve entità (raffreddore). L'aspetto dei coronavirus è caratterizzato dalla presenza di "spicole" sulla superficie esterna dei virus, che creano una sorta di "corona", da cui deriva il loro nome (Murray, 2018; Berkowitz & Jerris, 2016; Figura 3).

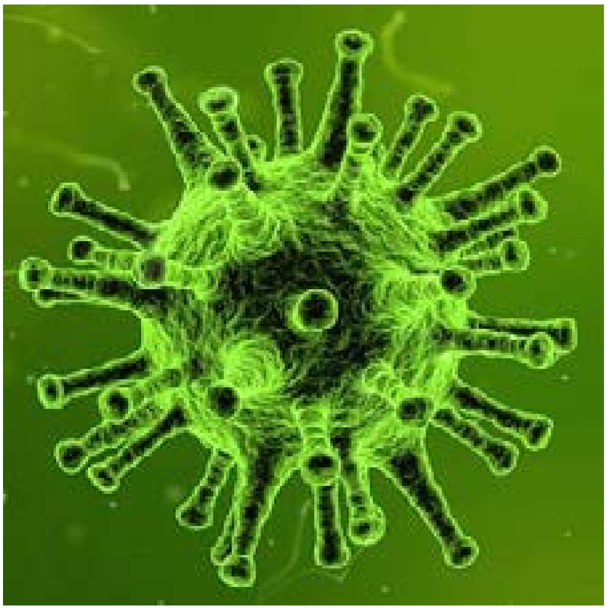

Figura 3. *Aspetto del nuovo coronavirus COVID-19, con la presenza di "spicole" sulla superficie (fonte: pixabay.com)*

Esistono in totale **4 famiglie di coronavirus**, ma solo due sono in grado di dare infezioni nell'uomo (alfa e beta) e sono definiti coronavirus umani comuni (Epicentro, 2020d):

- 229E (coronavirus alpha)
- NL63 (coronavirus alpha)
- OC43 (coronavirus beta)
- HKU1 (coronavirus beta)

Negli ultimi decenni tre virus appartenenti a questo gruppo, però, hanno causato **sindromi respiratorie di maggiore gravità** (Murray, 2018; Berkowitz & Jerris, 2016; Epicentro, 2020d):

- la SARS-CoV (*Severe Acute Respiratory Syndrome) nel 2002*
- *la MERS*-CoV (*Middle East Respiratory Syndrome*) nel 2013
- la SARS-CoV-2 (*CoronaVirus Disease*) nel 2019

Queste ultime si caratterizzano per l'insorgenza di una grave infezione in grado di determinare la morte in una percentuale elevata di soggetti e per l'origine dei virus, che sembra riconducibile ai **pipistrelli** (Murray, 2018; Berkowitz & Jerris, 2016; Epicentro, 2020d).

La **trasmissione** dei coronavirus umani da una persona all'altra avviene attraverso (Epicentro, 2020e):

- goccioline aero-trasmesse durante starnuti o colpi di tosse
- saliva
- stretti contatti personali diretti (convivenza, dialogo a distanza ravvicinata)
- contatto con bocca, naso o occhi attraverso le mani contaminate
- contaminazione fecale (rara)

I **sintomi e i segni** più comuni nell'uomo sono rappresentati da (Epicentro, 2020f):

- gocciolamento nasale
- mal di gola
- tosse
- mal di testa
- sensazione di malessere generale
- febbre

Nei **casi più gravi** si possono, invece, osservare (Epicentro, 2020f):

- polmonite
- febbre alta
- difficoltà respiratorie fino all'insufficienza respiratoria
- decesso

Il **punto di partenza** dell'**infezione da nuovo coronavirus è stato identificato nel mercato del pesce della città di Wuhan** (**Figura 4**), in Cina, nel dicembre 2019 (Epicentro, 2020d; Wang et al., 2020; Huang et al., 2020). All'inizio è stata individuata una connessione tra le varie persone che sviluppavano l'infezione e il mercato di Wuhan dove vi era un gran numero di animali vivi, suggerendo così una **trasmissione del virus dagli animali all'uomo**. Successivamente, però, si è iniziato a registrare un numero sempre maggiore di persone con l'infezione respiratoria ma senza nessun legame con il mercato, facendo ipotizzare la **trasmissione da uomo a uomo** (Center of Disease Control and Prevention, 2020).

L'Organizzazione Mondiale della Sanità ne ha dato notizia a inizio gennaio 2020, dichiarando che le autorità sanitarie in Cina avevano identificato un **nuovo coronavirus**, responsabile di un focolaio di polmonite. Il nuovo virus è stato denominato **SARS-CoV-2** e la **malattia respiratoria** da esso causata **COVID-19** (*Coronavirus Disease 2019*; Center of Disease Control and Prevention, 2020; Epicentro, 2020d; Maraolo, 2020a). Da Wuhan l'infezione si è progressivamente propagata alle altre città Cina, all'Europa, agli Stati Uniti e in moltissimi Paesi del mondo (Maraolo, 2020a).

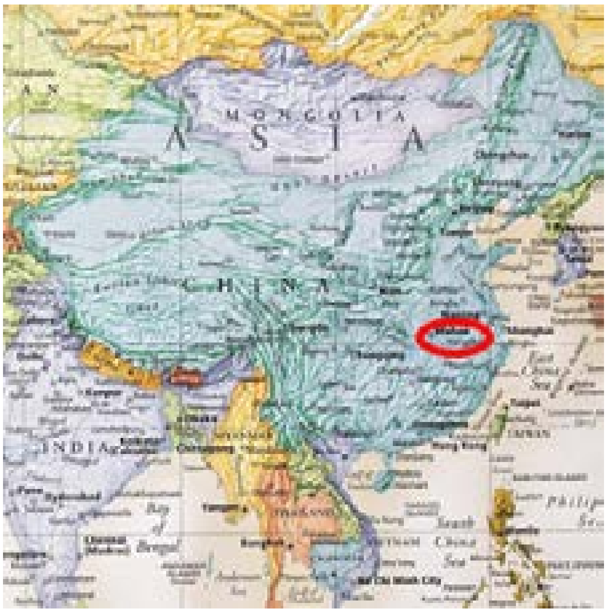

Figura 4. *Localizzazione della città di Wuhan in Cina*

Come per gli altri coronavirus l'espressione della malattia COVID-19, sotto forma di **infezione respiratoria**, ha un'ampia variabilità e può presentarsi in **forma** (Center of Disease Control and Prevention, 2020; Epicentro, 2020f; Maraolo, 2020a):

- asintomatica (completa assenza di sintomi)
- lieve (sintomi simili a raffreddore o influenza)
- grave (necessità di ricovero in ospedale)
- estremamente grave (decesso)

È importante precisare che è stato segnalato che anche le **persone che non presentano sintomi** (asintomatici) possono **trasmettere il virus** ad altri individui e contribuire così alla diffusione dell'infezione (Epicentro, 2020e).

Le persone nelle quali sono state osservate le **forme più gravi della malattia** sono individui di età più avanzata, in gran parte uomini, generalmente con altre malattie come patologie cardiache, patologie polmonari e diabete mellito (Wang et al., 2020; Center of Disease Control and Prevention, 2020; Parrino, 2020).

In Italia i primi casi di COVID-19, due turisti provenienti dalla Cina, sono stati identificati a Roma a fine gennaio 2020 (Epicentro, 2020e). Il primo caso locale, invece, si è verificato a fine febbraio 2020: un uomo di 38 anni ha fatto accesso all'ospedale di Codogno, in provincia di Lodi in Lombardia, per gravi difficoltà respiratorie ed è stata diagnosticata la COVID-19.

Da quel momento si è osservato un numero progressivamente maggiore di casi nelle regioni del nord dell'Italia, in particolare in Lombardia, in Veneto e in Emilia-Romagna (Maraolo, 2020b). Anche nelle altre regioni italiane si è osservato un aumento dei casi di infezione, che è risultato, al 16 marzo 2020, ancora contenuto rispetto a quanto osservato nelle regioni dell'Italia settentrionale (Epicentro, 2020g, 2020h; Figura 5).

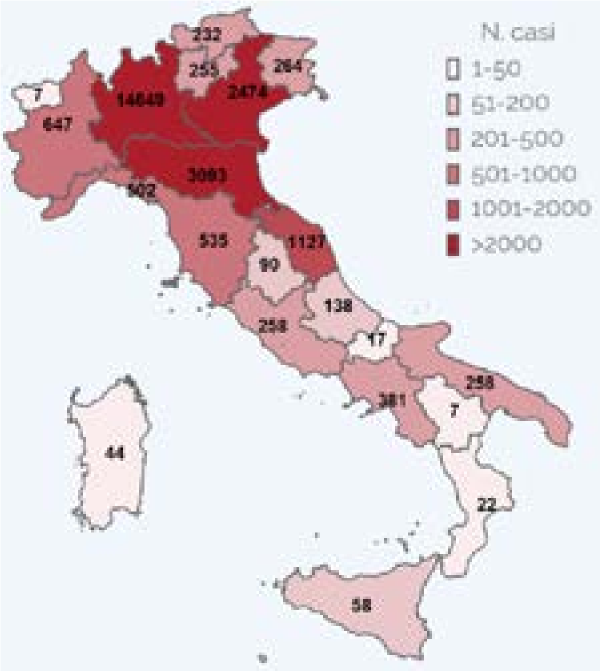

Figura 5. *Numero totale di casi COVID-19 per regione diagnosticati dai laboratori regionali di riferimento aggiornati al 16 marzo 2020 (fonte: Epicentro, 2020g)*

L'Istituto Superiore di Sanità riportava, al 16 marzo 2020 (Epicentro, 2020g):

- un numero totale di **25.058 casi** di COVID-19 in Italia
- 2.339 casi di operatori sanitari infettati
- un numero complessivo di 1.697 decessi

Il 37.4% delle persone con infezione COVID-19 aveva un'età superiore ai 70 anni, il 37.3% un'età compresa tra i 51 e i 70 anni, il 24.2% un'età compresa tra i 19 e i 50 anni e l'1.1% un'età inferiore ai 18 anni. Gli uomini sono risultati maggiormente interessati dall'infezione rispetto alle donne (Epicentro, 2020g).

È stato suggerito che in Italia la trasmissione dell'infezione COVID-19 sia avvenuta nel territorio nazionale, a differenza dei primi tre casi identificati nella regione Lazio che, invece, si pensa abbiano contratto l'infezione in Cina (Istituto Superiore di Sanità, 2020).

In una percentuale importante di casi l'infezione ha richiesto il ricovero in ospedale e nei casi particolarmente critici il trasferimento in Terapia Intensiva per garantire un supporto alla respirazione (Istituto Superiore di Sanità, 2020).

Attualmente **non esistono vaccini né terapie specifiche** per l'infezione COVID-19, ma si agisce cercando di alleviare i sintomi delle persone affette e di supportare la respirazione nei casi in cui si sviluppi insufficienza respiratoria (Epicentro, 2020e). La comunità scientifica si è messa all'opera per poter sviluppare un vaccino e per cercare di individuare e approvare degli schemi di terapia (farmaci antivirali, farmaci attivi su altre malattie).

1.5.3 La pandemia

A causa dell'aumento dei casi di COVID-19 all'interno dei territori nazionali e a causa dell'estensione dell'infezione ad un numero sempre maggiore di Paesi nel mondo, l'Organizzazione Mondiale della Sanità, a gennaio 2020, si è espressa dichiarando che l'infezione da SARS-CoV-2 rappresentava un'emergenza internazionale per la salute pubblica (Center of Disease Control and Prevention, 2020).

A metà marzo 2020, per via dell'interessamento esteso a tutto il mondo, l'Organizzazione Mondiale della Sanità ha, invece, definito la COVID-19 una **pandemia** (WHO, 2020b; Figura 6).

Le **pandemie** si verificano quando nuovi *microrganismi patogeni*, in questo caso un nuovo virus, infettano gli individui e si diffondono nelle popolazioni prive di immunità (Center of Disease Control and Prevention, 2020; WHO, 2020a). In passato sono state osservate altre pandemie sostenute da virus dell'influenza, ma questa è la **prima pandemia** sostenuta da un **nuovo coronavirus** (Center of Disease Control and Prevention, 2020).

Sono stati riportati casi di infezione in 114 Paesi del mondo, con più del 90% dei casi principalmente concentrati in quattro Paesi (WHO, 2020c).

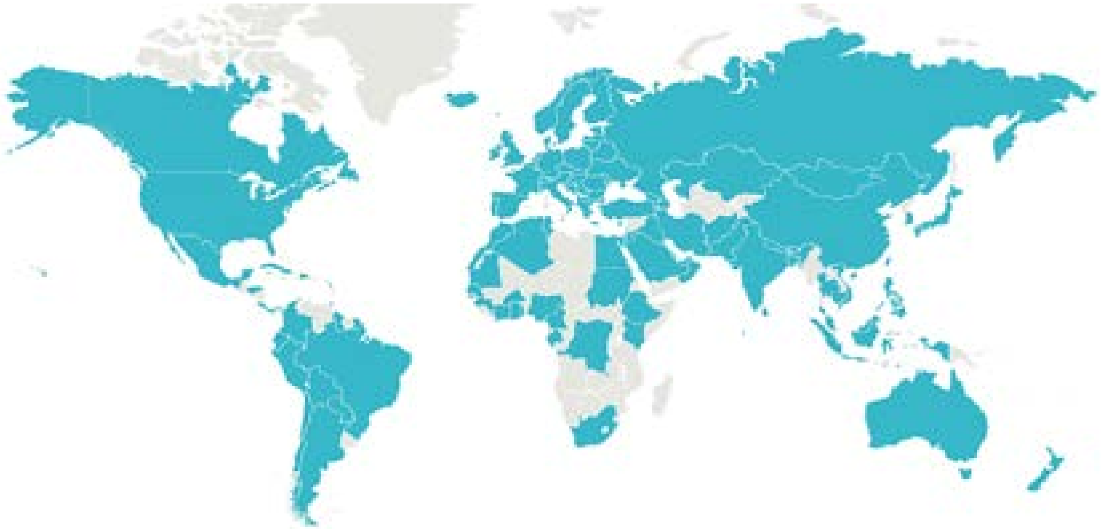

Figura 6. *Aree del mondo interessate da COVID-19 (aggiornato al 16 Marzo 2020) (fonte: Center of Disease Control and Prevention, 2020)*

L'Organizzazione Mondiale della Sanità si è espressa favorevolmente nei confronti delle misure adottate da Iran, Italia, Korea e Cina per contenere e prevenire l'infezione da SARS-CoV-2, e ha invitato tutti i Paesi ad agire in maniera urgente e aggressiva, pur riconoscendo il potenziale peso di queste azioni sulla società e sull'economia (WHO, 2020b).

Il Direttore Generale dell'Organizzazione Mondiale della Sanità ha, quindi, esortato tutti i Paesi a (WHO, 2020b):

1. Prepararsi ed essere pronti
2. Identificare, proteggere e trattare
3. Ridurre la trasmissione del virus
4. Innovare e imparare

1.6 Le indicazioni del Ministero della Salute per contrastare l'infezione da nuovo coronavirus in Italia

In Italia il Ministero della Salute ha messo a disposizione dei cittadini una serie di risorse per potersi documentare e agire responsabilmente (Ministero della Salute, 2020).

In data **17 Febbraio 2020** è stato formulato un elenco con i **"Dieci comportamenti da seguire" (Box 1**).

Il **6 marzo 2020** sono state rese disponibili le **"Raccomandazioni per la prevenzione" (Box 2**), che confermavano alcune indicazioni già presenti nell'elenco con i "Dieci comportamenti da seguire" e introducevano il tema del **distanziamento sociale**, al fine di **contenere il contagio** da nuovo coronavirus (Ministero della Salute, 2020).

Il **9 marzo 2020** è stato, inoltre, preparato un "**Vademecum" (Box 3**) che raccoglieva le risposte alle domande più frequenti sull'infezione (Ministero della Salute, 2020).

In data **11 marzo 2020**, è stato pubblicato sul sito del Ministero della Salute anche il documento "**Domande frequenti ai tempi del coronavirus"** ed è partita la campagna **"#iorestoacasa"** che invitava i cittadini a rimanere presso il proprio domicilio, per contribuire a contenere la diffusione dell'infezione.

Indicazioni analoghe sono disponibili anche sul sito della Organizzazione Mondiale della Sanità in lingua inglese (WHO, 2020c).

Tutti i cittadini italiani sono stati chiamati ad agire con senso civico e nel rispetto della salute della collettività, al fine di contenere l'infezione.

Tutti gli sforzi delle comunità scientifiche sono volti all'identificazione di terapie efficaci e alla preparazione di vaccini per cercare di debellare questa infezione responsabile della pandemia del 2020.

Box 1. Nuovo coronavirus – Dieci comportamenti da seguire
(Ministero della Salute, 2020)

- Lavati spesso le mani con acqua e sapone o con gel a base alcolica
- Evita il contatto ravvicinato con persone che soffrono di infezioni respiratorie acute
- Non toccarti occhi, naso e bocca con le mani
- Copri bocca e naso con fazzoletti monouso quando starnutisci o tossisci. Se non hai un fazzoletto usa la piega del gomito
- Non prendere farmaci antivirali né antibiotici senza la prescrizione del medico
- Pulisci le superfici con disinfettanti a base di cloro o alcol
- Usa la mascherina solo se sospetti di essere malato o se assisti persone malate
- I prodotti MADE IN CHINA e i pacchi ricevuti dalla Cina non sono pericolosi
- Gli animali da compagnia non diffondono il nuovo coronavirus
- In caso di dubbi non recarti al pronto soccorso, chiama il tuo medico di famiglia e segui le sue indicazioni

Box 2. Raccomandazioni per la prevenzione
(Ministero della Salute, 2020)

- Lavati spesso le mani con acqua e sapone o con gel a base alcolica
- Non toccarti occhi, naso e bocca con le mani
- Copri bocca e naso con fazzoletti monouso quando starnutisci o tossisci. Altrimenti usa la piega del gomito
- Evita i contatti ravvicinati mantenendo la distanza di almeno un metro
- Evita luoghi affollati
- Evita le strette di mano e gli abbracci fino a quando questa emergenza sarà finita
- Se hai sintomi simili all'influenza resta a casa, non recarti al pronto soccorso o presso gli studi medici, ma contatta il medico di medicina generale, i pediatri di libera scelta, la guardia medica o i numeri regionali

Box 3. Vademecum
(Ministero della Salute, 2020)

- **Quali sono i sintomi a cui devo fare attenzione?**

Febbre e sintomi simil-influenzali come tosse, mal di gola, respiro corto, dolore ai muscoli, stanchezza sono segnali di una possibile infezione da nuovo coronavirus.

- **Ho febbre e/o sintomi influenzali, cosa devo fare?**

Resta in casa e chiama il medico di famiglia, il pediatra o la guardia medica.

- **Dopo quanto tempo devo chiamare il medico?**

Subito. Se ritieni di essere contagiato chiama appena avverti i sintomi di infezione respiratoria, spiegando i sintomi e i contati a rischio.

- **Non riesco a contattare il mio medico di famiglia, cosa devo fare?**

Chiama uno dei numeri di emergenza indicati sul sito www.salute.gov.it/nuovocoronavirus

- **Posso andare direttamente al pronto soccorso o dal mio medico di famiglia?**

No. Se accedi al pronto soccorso o vai in ambulatorio senza prima averlo concordato con il medico potresti contagiare altre persone.

- **Come posso proteggere i miei familiari?**

Segui sempre i comportamenti di igiene personale (lavati regolarmente le mani con acqua e sapone o usa un gel a base alcolica) e mantieni pulito l'ambiente. Se pensi di essere infetto indossa una mascherina chirurgica, resta a distanza dai tuoi familiari e disinfetta spesso gli oggetti di uso comune.

- **Dove posso fare il test?**

I test vengono eseguiti unicamente in laboratori del Servizio Sanitario Nazionale selezionati. Se il tuo medico ritiene che sia necessario un test ti fornirà indicazioni su come procedere.

- **Dove trovo altre informazioni attendibili?**

Segui solo le indicazioni specifiche e aggiornate dei siti web ufficiali, delle autorità locali e della Protezione Civile.

1.7 Conclusioni

Questo capitolo ha presentato alcune informazioni scientifiche essenziali sui diversi tipi di microrganismi presenti in natura, sulle infezioni respiratorie di origine virale e sulla recente pandemia da nuovo coronavirus allo scopo di fornire all'insegnante gli strumenti per avviare un dialogo con gli alunni basato sulle evidenze scientifiche a disposizione. Ciascun docente potrà utilizzare tali informazioni per gestire la comunicazione con i propri alunni nei periodi successivi al rientro in classe dopo l'emergenza causata dal nuovo coronavirus. Come spiegato all'inizio del capitolo, non tutti i microrganismi hanno effetti nocivi per l'uomo. Appare dunque importante saper gestire una comunicazione equilibrata con agli alunni, evitando sia messaggi eccessivamente allarmistici (esempio: "bisogna eliminare sempre tutti i microrganismi"), e quelli non rappresentativi delle conoscenze scientifiche disponibili (esempio "è sempre necessario assumere l'antibiotico anche in caso di infezioni virali"), sia comportamenti di diniego che possono in realtà favorire il diffondersi delle infezioni.

1.8 Dizionario medico

Acido nucleico: materiale genetico, che può essere rappresentato da DNA o RNA, e che funziona come una sorta di "libretto di istruzioni" per gli organismi.

Antibiotico: sostanza di origine naturale, batterica o fungina, capace di contrastare la crescita di altri microrganismi (batteri).

Chemioterapico antibatterico: molecola prodotta sinteticamente capace di contrastare la crescita di altri microrganismi (batteri).

Eucarioti: organismi con un nucleo ben riconoscibile e con strutture cellulari ben definite.

Immunosoppressione: condizione di ridotto funzionamento del sistema immunitario dovuta a malattie o a farmaci che riducono l'efficacia del sistema immunitario.

Infezioni endogene: malattie causate da microrganismi generalmente presenti nel corpo umano.

Infezioni esogene: malattie causate da microrganismi presenti nell'ambiente.

Microbioma: insieme di batteri, funghi e parassiti, ospitato dal corpo umano in varie sedi, che contribuisce al mantenimento di un buono stato di salute.

Microrganismo patogeno: microrganismo in grado di causare malattie nell'uomo.

Mucosa: rivestimento interno degli organi cavi che comunicano con l'esterno (esempio bocca, apparato digerente, apparato respiratorio).

Multicellulare: costituito da più cellule.

Parassiti endocellulari obbligati: microrganismi completamente dipendenti dalle cellule viventi per poter sopravvivere e moltiplicarsi.

Periodo di incubazione: tempo che trascorre tra l'incontro con un microrganismo responsabile di infezione e la comparsa di segni e i sintomi caratteristici della malattia.

Sieroprofilassi: forma di immunizzazione passiva che prevede il trasferimento di anticorpi da donazioni di sangue.

Unicellulare: costituito da una sola cellula.

Vettori: organismi in grado di trasportare microrganismi patogeni all'interno del corpo umano.

Zoonosi: malattie infettive trasmesse dagli animali all'uomo.

Bibliografia del capitolo

Agenzia Italiana del Farmaco (2020, 26 marzo). www.aifa.gov.it/farmaci-antibiotici

Berkowitz, F. E., & Jerris, R. C. (2016). Practical Medical Microbiology for Clinicians. John Wiley & Sons. ISBN 978-1-119-06674-3

Centers for Disease Control and Prevention (2020, 21 marzo). *Situation Summary.* https://www.cdc.gov/coronavirus/2019-ncov/cases-updates/summary.html?CDC_AA_refVal=https%3A%2F%2Fwww.cdc.gov%2Fcoronavirus%2F2019-ncov%2Fsummary.html

Epicentro (2020a). *Le malattie infettive.* https://www.epicentro.iss.it/infettive/

Epicentro (2020b). *Zoonosi - Istituto Superiore di Sanità.* https://www.epicentro.iss.it/zoonosi

Epicentro (2020c). *Sindromi parainfluenzali precauzioni e trattamento.* https://www.epicentro.iss.it/parainfluenzali/precauzioni

Epicentro (2020d). *Coronavirus - Cosa sono i coronavirus.* https://www.epicentro.iss.it/coronavirus/cosa-sono

Epicentro (2020e). *Coronavirus - Trasmissione, prevenzione e trattamento.* https://www.epicentro.iss.it/coronavirus/trasmissione-prevenzione-trattamento

Epicentro (2020f). *Coronavirus - Sintomi e diagnosi.* https://www.epicentro.iss.it/coronavirus/sintomi-diagnosi

Epicentro (2020g).https://www.epicentro.iss.it/coronavirus/bollettino/Infografica

Epicentro (2020h). *Sorveglianza integrata COVID-19: i principali dati nazionali.* https://www.epicentro.iss.it/coronavirus/sars-cov-2-sorveglianza-dati

Huang, C., Wang, Y., Li, X., Ren, L., Zhao, J., Hu, Y., ... & Cheng, Z. (2020). Clinical features of patients infected with 2019 novel coronavirus in Wuhan, China. The Lancet, 395(10223), 497-506.

Istituto Superiore di Sanità, (2020, 12 marzo). *Epidemia COVID-19 Aggiornamento nazionale 12 marzo 2020 – ore 16:00.*

Lanciotti, E. (2017). Microbiologia clinica. IV Edizione, Casa editrice ambrosiana. EAN: 9788808181855

Maraolo, A. E. (2020a). COVID-19: Italia in prima linea contro il nuovo coronavirus. Medici Oggi. https://medicioggi.it/contributi-scientifici/covid-19-italia-in-prima-linea-contro-il-nuovo-coronavirus/

Maraolo, A. E. (2020b). Infezione da coronavirus 2019-nCoV–il mondo con il fiato sospeso. Medici Oggi. https://medicioggi.it/contributi-scientifici/infezione-da-coronavirus-2019-ncov-il-mondo-con-il-fiato-sospeso/

Ministero della salute (2020). *Nuovo coronavirus.* http://www.salute.gov.it/nuovocoronavirus

Murray, P. R. (2018). Basic Medical Microbiology. Elsevier Health Sciences. ISBN: 978-0-323-47676-8

Parrino, C. (2020). Diabete mellito, infezioni respiratorie e Sindrome COVID-19: evidenze disponibili e consigli pratici per la gestione delle persone con diabete durante il ricovero ospedaliero. Medici Oggi. https://medicioggi.it/contributi-scientifici/diabete-mellito-infezioni-respiratorie-e-sindrome-covid-19-evidenze-disponibili-e-consigli-pratici-per-la-gestione-delle-persone-con-diabete-durante-il-ricovero-ospedaliero/

Treccani (2020). *MICRORGANISMI in "Enciclopedia Italiana." www.treccani.it/enciclopedia/microrganismi_%28Enciclopedia-Italiana%29/*

Wang, D., Hu, B., Hu, C., Zhu, F., Liu, X., Zhang, J., ... & Zhao, Y. (2020). Clinical characteristics of 138 hospitalized patients with 2019 novel coronavirus–infected pneumonia in Wuhan, China. Jama.

World Health Organization (2020a, 12 marzo). *What is a pandemic?* https://www.who.int/csr/disease/swineflu/frequently_asked_questions/pandemic/en/

World Health Organization (2020b). *WHO Director-General's opening remarks at the media briefing on COVID-19 - 23 March 2020.* https://www.who.int/dg/speeches/detail/who-director-general-s-opening-remarks-at-the-media-briefing-on-covid-19---11-march-2020

World Health Organization (2020c). *Advice for public.* https://www.who.int/emergencies/diseases/novel-coronavirus-2019/advice-for-public

World Health Organization. (2001). Infections and infectious diseases: a manual for nurses and midwives in the WHO European Region (No. EUR/01/5019329). Copenhagen: WHO Regional Office for Europe.

Capitolo 2

La comprensione della malattia nel bambino[1]

Michele Capurso

La malattia rappresenta per il bambino una specie di evento incidentale, un'interruzione di quella rassicurante routine fatta di progetti, desideri, amicizie e gioco che consentono il naturale sviluppo della propria vita (Bianchi di Castelbianco, Capurso, & Di Renzo, 2007). La prima cosa che gli adulti pensano quando sentono parlare di una malattia riguarda la sua gravità e le possibili cure. Per i bambini non è sempre e necessariamente così. Da un lato essi tendono a vivere molto di più nel presente, preoccupandosi, ad esempio, se la malattia andrà a compromettere le loro capacità di giocare, incontrare gli amici, o partecipare alla vita scolastica (Capurso, Lo Bianco, Cortis, & Rossetti, 2016); dall'altro l'incapacità di trovare spiegazioni logiche alla malattia può generare soprattutto nei più piccoli vissuti di ansia o sensi di colpa (Dempsey, 2019; Eiser, 1985b).

Spesso gli adulti che si occupano di una malattia adottano un approccio di tipo biomedico, vale a dire centrato sulla ricerca scientifica della causa del malessere e sulla individuazione della terapia medica più efficace per curare la malattia (Shorter, 2005). Le attenzioni riservate a chi si ammala si concretizzano spesso in risposte di tipo fisico-chimico, tese a minimizzare i sintomi e a curare la patologia. Questo tipo di approccio è naturalmente molto efficace nel trattare la malattia dal punto di vista organico, ma tende a trascurare la dimensione psicologica e sociale legata alla malattia (Engel, 1977). Si rischia cioè di dimenticarsi che ogni individuo, sano o malato che sia, è anche portatore di una forza vitale che lo spinge verso lo sviluppo, verso la crescita e la piena realizzazione dei propri potenziali. Freud chiamava questa forza «Eros» (Freud, 1920/2013) e la ricollegava al desiderio umano di creare, produrre e costruire. Gli autori della psicologia umanistica, Maslow e Rogers, la chiameranno «tendenza attualizzante» (Maslow, 1954/2010; Rogers, Kinget, & Galli, 1970).

---

[1] Questo capitolo si basa in parte sul capitolo di M. Capurso (2007), "Io e la mia malattia", inserito nel testo a cura di F. Bianchi di Castelbianco, M. Capurso, M. Di Renzo (eds.), *Ti racconto il mio ospedale: esprimere e comprendere il vissuto della malattia*, Roma, Magi. Ringrazio l'editore Magi per avermi consentito di riprendere e aggiornare questo lavoro.

I bambini che rientrano a scuola dopo un lungo periodo di assenza dovuto alla pandemia di Covid-19 non hanno, generalmente, vissuto esperienze gravi di malattia. Hanno però vissuto delle esperienze piuttosto prolungate di limitazioni sul piano

biopsicosociale. Sono stati costretti ad un'assenza forzata e avranno assorbito in modo vicario l'ansia e le preoccupazioni dei loro genitori e parenti (Nolte, Guiney, Fonagy, Mayes, & Luyten, 2011). Infatti la situazione di crisi collettiva cui tutti siamo stati sottoposti è stata vissuta anzitutto tramite delle categorie affettive e oltretutto ci ha colpito in modo collettivo, mettendo in discussione il nostro mondo quotidiano, fatto di routine e certezze ed esponendoci ad un prolungato e quotidiano senso di insicurezza e vulnerabilità (Aptekar & Boore, 1990). Nei più piccoli, la pandemia causata dal nuovo coronavirus ha sospeso con l'isolamento forzato il normale processo di sviluppo psicosociale e ha generato, per molto tempo, un prolungato senso di insicurezza. La campagna mediatica #andratuttobene, partita dalla città di Modugno (Bari) l'11 marzo 2020 (ANSA), rappresenta la voce di resilienza, incoraggiamento e vitalità che i bambini d'Italia vogliono far sentire agli adulti ma forse anche a loro stessi. È come se volessero rispondere così all'ordine/consiglio del governo #iorestoacasa. Non in modo polemico, ma dichiarando un'energia costruttiva, come a voler affermare la propria forza vitale ed evolutiva tramite la certezza di un futuro positivo, un sole e un arcobaleno sopra le nuvole del momento, che tra poco tornerà a risplendere per tutti.

In questo caso, la serietà della situazione deriva non tanto dalla gravità della malattia, quanto dall'estensione territoriale, numerica e temporale che i provvedimenti di contenimento hanno avuto. Per molti giorni tutti i bambini d'Italia non hanno potuto incontrare i loro amici, giocare in gruppo, andare a scuola, frequentare attività pomeridiane. Il terzo capitolo di questo libro ci spiega come la scuola può rispondere a tutto questo, ma prima di affrontare questo argomento è necessario capire in che modo i bambini giungono a costruire la loro comprensione della malattia.

2.1 Due modelli nella comprensione della malattia nel bambino

La comprensione della malattia è il risultato personale di un processo di costruzione di senso. Il concetto di malattia che si arriva ad elaborare si basa sulle conoscenze e sulle credenze che le persone possiedono in relazione ai sintomi, a quella determinata malattia, allo stato di salute e alla medicina in generale (Friedman, 2013). Come vedremo si tratta quindi di un processo che, soprattutto nei bambini, appare strettamente legato ad aspetti culturali e di vita quotidiana.

Sostanzialmente possiamo indicare due modelli che spiegano in che modo il bambino possa giungere a comprendere i concetti legati alla malattia (Capurso, 2019; Eiser, 1985a; Rushforth, 1999).

2.1.1 Lo sviluppo mentale e la comprensione della malattia secondo il modello di Piaget

Il primo di questi modelli si basa sulla teoria di Piaget e connette la comprensione dei meccanismi che possono generare una malattia agli stadi evolutivi delineati dall'autore (Piaget, 1953). Come è noto, secondo Piaget, all'interno dei diversi stati evolutivi che ogni bambino attraversa nel corso del suo sviluppo, il pensiero assume forme specifiche qualitativamente diverse da quelle di fasi precedenti (vedi Tabella 2.1).

Tabella 2.1 *Gli stadi evolutivi di Piaget e le relative caratteristiche del pensiero infantile (fonte: tradotto e adattato da Shaffer & Kipp, 2013)*

**Età approssimativa**	**Stadio**	**Schemi cognitivi principali e modalità dirappresentazione dell'esperienza**	**Principali traguardi evolutivi**
Dalla nascita a 2 anni	Sensomotorio	I neonati dispongono di una serie di riflessi innati che gli consentono di iniziare una prima interazione con il mondo circostante. Il loro pensiero è strettamente legato all'azione sugli oggetti e sul mondo.<br>Gradualmente, iniziano ad usare le loro abilità motorie e sensoriali per esplorare il mondo circostante e acquisire delle conoscenze di base sul suo funzionamento. Verso la fine di questo stadio divengono progressivamente più capaci di coordinarsi per svolgere azioni sensomotorie sempre più complesse.	I bambini acquisiscono un primitivo senso del "sé" e degli "altri". Comprendono che il proprio corpo ha dei confini e delle caratteristiche ben definite. Intorno ai due anni d'età matura la permanenza dell'oggetto, cioè la consapevolezza che gli oggetti continuano ad esistere anche quando non li si vedono. Contemporaneamente, il bambino inizia ad interiorizzare schemi comportamentali e immagini mentali del mondo esterno.

Dai 2 ai 6 anni	Preoperatorio	Compare il pensiero simbolico, cioè la capacità di usare immagini o parole per rappresentare e comprendere determinati aspetti del mondo circostante. Il pensiero è inizialmente egocentrico, cioè incapace di prendere in considerazione il punto di vista degli altri, e solo verso la fine dello stadio compare la capacità di iniziare a tenere presente il punto di vista degli altri. Il bambino confonde spesso il piano morale e il piano fisico degli eventi.	Il bambino diventa capace di fare giochi fantastici (il manico di scopa diventa un cavallo), di imitare azioni in modo differito nel tempo, di usare il linguaggio per evocare eventi passati o per anticipare eventi futuri.<br>Il pensiero di questa età è caratterizzato da:<br>*Finalismo,* cioè dal fatto che i fenomeni naturali hanno come centro l'uomo (la pallina rotola perché vuole far giocare il bambino)<br>*Animismo*: le cose sono concepite come viventi e dotate di intenzionalità (il sole è rappresentato con gli occhi e la bocca).<br>*Artificialismo*: tutto l'universo è stato costruito secondo le regole della costruzione umana (le valli sono stata scavate e la terra avanzata è stata usata per formare le montagne).
dai 6 ai 12 anni	Operatorio-concreto	Compare la capacità di effettuare operazioni logiche e risolvere compiti e problemi, (somme, sottrazioni, raggruppamenti in base a criteri ordinatori, seriazione, reversibilità), ma questi devono di solito appoggiarsi a riferimenti concreti.	I bambini divengono progressivamente più abili nella loro capacità di osservare il mondo esterno e di dedurre leggi e funzionamento delle cose. Si usano i rapporti causali per spiegare gli eventi, ma il pensiero è ancora legato agli oggetti reali.
dai 12 anni in poi	Operatorio-formale	Si diventa capaci di pensare in modo astratto. Compaiono funzioni metacognitive (pensare al come si pensa).	L'adolescente diviene gradualmente capace di pensare eventi, oggetti, e concetti astratti. Sa organizzare e connettere tra loro le informazioni in modo sistematico, riconoscendo le relazioni tra più parti. Il pensiero può diventare idealistico, slegato dalla concretezza delle cose. Compare la capacità di ponderare soluzioni diverse al medesimo problema.

Gli stadi piagetiani si muovono da una dimensione che inizialmente è puramente senso-motoria, per poi attraversare, tra i 2 e i 7/8 anni, una fase detta simbolica o preoperatoria e proseguire tra gli 8 e gli 11 anni con uno stadio operatorio concreto che sfocerà, solo verso la preadolescenza, nello stato più maturo del pensiero operatorio-formale (vedi Tabella 2.1).

Sulla scia di questa teoria alcuni autori hanno voluto indagare la comprensione della malattia dei bambini di diverse età. Secondo Bibace and Walsh (1981), le spiegazioni che i bambini danno sulle cause delle malattie sono raggruppabili in base ad alcune categorie specifiche, che hanno una logica legata allo stadio di sviluppo mentale e quindi all'età dei soggetti. Più precisamente, le credenze dei bambini rispetto alla malattia riflettono il tipo di logica che essi adoperano per descrivere concetti fisici come il principio di conservazione della quantità o quello di causalità. Per svolgere la loro ricerca gli autori hanno intervistato un gruppo di 180 bambini e ragazzi aventi dai 4 ai 14 anni d'età, chiedendo loro quali fossero le ragioni per cui ci si ammala. Sulla base di questo hanno individuato 7 tipologie di spiegazione corrispondenti ai diversi stadi evolutivi piagetiani (Tabella 2.2).

Tabella 2.2 *I diversi stadi evolutivi di Piaget e le corrispondenti spiegazioni della malattia date dai bambini secondo Bibace e Walsh (1980, 1981) Tradotto e riadattato (Capurso, 2001).*

Età	Stadio evolutivo secondo Piaget	Categoria di comprensione secondo Bibace e Walsh	Esempio di spiegazione del bambino
0-2	Senso-motorio	Incomprensibile	--
2-7	Preoperatorio	Fenomenico	**Come si prende il raffreddore?**<br>*Dal sole.*<br>**Come fa il sole a darti il raffreddore?**<br>*Te lo dà e basta.*<br>**Come si prende il raffreddore?**<br>*Dagli alberi.*<br>**Come si prende il morbillo?**<br>*Da Dio.*<br>**Come fa Dio a dare il morbillo alle persone?**<br>*Lo fa nel cielo.*
		Contagio o magia	**Come si prende il raffreddore?**<br>Da fuori.<br>**Come si fa a prenderlo da fuori?**<br>*Si prende e basta. Ti viene perché qualcuno ti viene vicino.*<br>**Ma come fa?**<br>*Non so. Penso per magia.*

7-11	Operatorio-concreto	Contaminazione per contatto	**Che cos'è il raffreddore?**<br>*È quando c'è l'inverno...*<br>**Come si fa a prenderlo?**<br>*Quando tu esci senza un berretto e poi inizi a starnutire. La tua testa diventa fredda, perché il freddo la tocca e poi il freddo ti va in tutto il corpo.*
		Internalizzazione	**Che cos'è un raffreddore?**<br>*Fai un sacco di starnuti, parli in modo strano e il tuo naso è tutto otturato.*<br>**Come fa la gente a prendere il raffreddore?**<br>*Quando c'è l'inverno, si respira troppa aria nel naso e questa finisce per otturarlo.*<br>**Come fa questo a causarti il raffreddore?**<br>*I batteri ti entrano dentro quando respiri. Poi i polmoni diventano troppo molli (fa un sospiro) e ti va nel naso.*<br>**Come si fa a guarire?**<br>*Aria calda e pulita. Ti va nel naso e rimanda via l'aria fredda.*
11 +	Operatorio-Formale	Fisiologica	**Come fa la gente a prendersi il raffreddore?**<br>*Credo che sia causato da dei virus.*<br>*Altra gente ha questi virus e loro raggiungono il tuo sangue e ti causano il raffreddore.*<br>**Tu sei mai stato malato? Che problema avevi?**<br>*Il livello delle mie piastrine era troppo basso.*<br>**E cioè?**<br>*Nel sangue, sono un po' come i globuli bianchi, ti aiutano ad uccidere i microbi.*<br>**Perché ti sei ammalato?**<br>*C'erano più microbi che piastrine e le piastrine stavano morendo.*<br>**Come hai fatto ad ammalarti?** *Per via dei microbi. Stavano uccidendo le piastrine.*
		Psico-fisiologica	**Che cos'è un attacco di cuore?**<br>*È quando il tuo cuore non funziona più bene.*<br>*Qualche volta batte troppo piano, altre volte troppo forte.*<br>**Come fa la gente ad avere un attacco di cuore?**<br>*Può succedere perché i tuoi nervi sono troppo tesi. Uno si preoccupa troppo e la tensione può far male al tuo cuore.*

2.1.2 Alcuni esempi delle spiegazioni date dai bambini

I bambini tra i due/tre e i sette-otto anni che appartengono, secondo la teoria di Piaget, allo stadio preoperatorio dello sviluppo cognitivo, raccolgono le informazioni attraverso i sensi e le comunicazioni verbali e non verbali, sono consapevoli principalmente dell'esperienza presente: per loro la causa delle malattie è un evento fenomenico, cioè riconoscibile tramite la propria esperienza sensoriale ed è riconducibile ad elementi di natura magica o naturali (Figura 2.1).

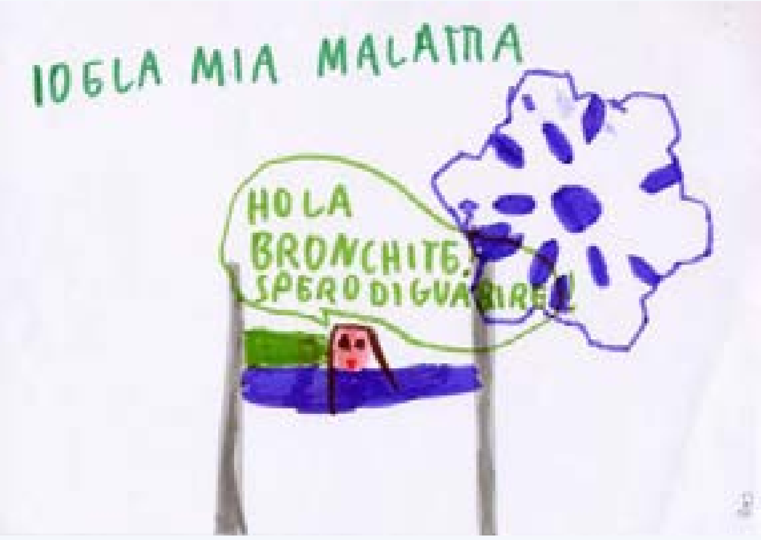

Figura 2.1 *Un esempio di rappresentazione preoperatoria della malattia.*

Maria Regina, 5 anni, rappresenta la sua malattia con una forma simmetrica, di colore viola, che ricorda quelle fotografie o disegni di virus che si possono vedere nei cartoni animati o su qualche poster o libro. Questo "virus" è chiaramente collocato al di fuori del suo corpo (in una zona del foglio dove spesso i bambini riproducono il sole), anche se giunge a toccare la spalliera del letto, arrivando quindi a trasmettere, in qualche modo, un suo flusso malefico. Maria Regina si rappresenta sdraiata a letto, con un corpo visibile e senza braccia, ma con un viso ben colorato e curato nei suoi tratti femminili. Si direbbe dunque che la malattia non sia giunta a compromettere l'Io del paziente, ma ne abbia alterato la percezione della capacità di muoversi e agire sul mondo.
Fonte: (Capurso, 2007, p. 36)

Quando il bambino raggiunge i 7-8 anni, è in grado di passare alle operazioni concrete del pensiero con la maturazione della capacità di differenziare sé dagli altri e di distinguere chiaramente tra l'interno e l'esterno della propria persona. Pur non essendo ancora in grado di esprimere ipotesi formali ha la consapevolezza che la malattia è localizzata all'interno del corpo, mentre la causa può essere esterna (Figura 2.2). Compare il concetto di contaminazione e si sviluppa un pensiero di tipo logico, che però è fortemente legato ad aspetti concreti (si veda anche il box 2.1).

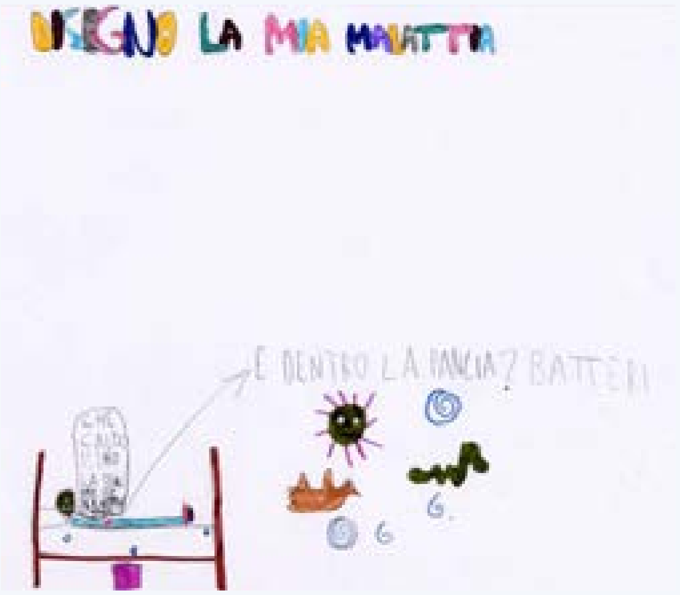

Figura 2.2 *Un esempio di rappresentazione concreta della malattia. Luca, 8 anni, collega chiaramente il suo stato di malessere ai batteri presenti all'interno della sua pancia.*
Fonte: (Capurso, 2007, p. 37)

Box 2.1 I dubbi di Andrea.

Un bambino di 7 anni, il cui fratello è morto a causa di un neuroblastoma addominale, era convinto di aver causato la malattia del fratello, perché un giorno aveva accarezzato un cane randagio e poi aveva toccato il fratello.

(Kreitler & Arush, 2004)

Nel periodo compreso tra gli 11 e i 15 anni i ragazzi acquistano una conoscenza sempre più esatta sulle specifiche strutture e funzioni fisiologiche degli organi interni. Si intuisce il complesso legame tra corpo e psiche: si identifica chiaramente l'organo malato, anche se si comprende che la causa può essere esterna ad esempio collegata ad un virus o ad una infezione (Box 2.2).

Box 2.2. Un esempio di rappresentazione logico-formale della malattia.

La leucemia è una malattia del sangue che ha origine dal midollo che quindi produce globuli bianchi malati e le piastrine sono basse. Fisicamente ho risentito della chemio perché uccideva i globuli bianchi e mi ammosciava i muscoli: infatti non ammazzava solo i bianchi malati, ma anche quelli buoni. Poi quando vedevo che le piastrine salivano ero più sollevato perché è segno che il midollo produce cellule buone. Io devo fare spesso il Micostatin, una medicina che devi mettere in bocca, tenere per cinque minuti e poi ingoiarlo. Serve per non avere micosi in bocca, è liquido e dolciastro, una vera schifezza per me. Ora sto prendendo il Purinetal che è una chemio orale che serve a distruggere le altre cellule tumorali.
Io sono all'inizio del secondo blocco, dopo devo fare il terzo e a metà del terzo devo fare il trapianto di midollo per evitare la ricaduta. Il trapianto consiste nel trovare un donatore che abbia il midollo uguale al tuo (nel mio caso Alessandra, mia sorella) e poi fare un prelievo di midollo al donatore e quindi la trasfusione. Il donatore dovrà essere ricoverato solo pochi giorni, mentre la trasfusione si verificherà per due giorni circa in una camera sterile e poi sarò ricoverato normalmente (Stefano, 10 anni).
Fonte: (Capurso, 2007, pp. 40-41)

Secondo Bibace e Walsh le conoscenze dei bambini sono quindi qualitativamente diverse da quelle degli adulti, perché si basano su strutture cognitive e forme di pensiero differenti che cambiano il modo di percepire e concettualizzare la realtà esterna. Solo un processo maturativo potrà portare il bambino al ragionamento operatorio formale caratteristico degli adulti.

Da questi studi iniziali sono poi stati sviluppati altri modelli che hanno ulteriormente specificato e analizzato i legami tra stati evolutivi e compressione della malattia. Ad esempio, Perrin and Gerrity (1981) hanno analizzato le cause per cui ci si ammala, gli scopi delle terapie e i sentimenti del personale sanitario (Brewster, 1982). Più recentemente, Koopman, Baars, Chaplin, and Zwinderman (2004) hanno ripreso i diversi stadi di comprensione della malattia già definiti da Bibace e Walsh e li hanno ridefiniti secondo una sequenza evolutiva che prende in esame la percezione della distanza tra agente patogeno e soggetto che si ammala, secondo una direzione che va dall'esterno all'interno della persona. Questo modello viene definito TEC (Through the Eyes of the Child) ed è presentato con l'ausilio di una immagine che ne riassume il significato nei diversi stadi (Tabella 2.4).

Tabella 2.4 *Le diverse fasce d'età e i corrispondenti livelli di comprensione della malattia secondo il modello TEC (Koopman et al., 2004) in (Capurso, 2017). Riproduzione delle immagini autorizzata.*

**Età**	**Categoria secondo il Modello TEC**	**Visualizzazione**
0-2	Invisibile	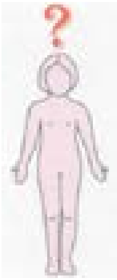
2-6	Distanza	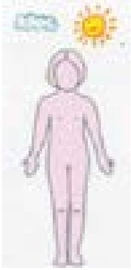
	Prossimità	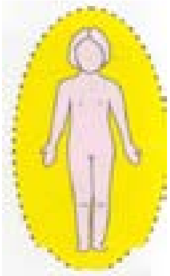
7-11	Contatto	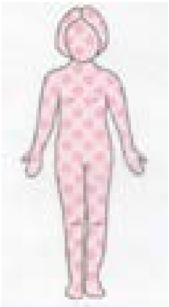
	Internalizzazione	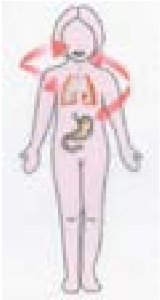

11 +	Interno del corpo	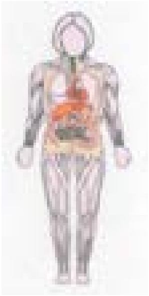
	Interno del corpo e della mente	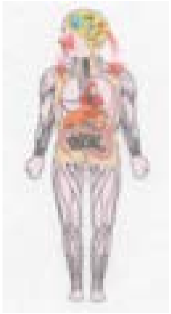

2.1.3 Le caratteristiche principali della comprensione della malattia secondo il modello piagetiano

Questo gruppo di lavori di matrice piagetiana presenta alcune caratteristiche comuni che possiamo così riassumere:

- Il modo di percepire e di rappresentare la realtà di malattia e le terapie, nei bambini è qualitativamente diverso da quello degli adulti (Bibace & Walsh, 1980; Crisp, Ungerer, & Goodnow, 1996; Eiser, 1985c).
- Anche se vi sono diverse eccezioni e numerose variabili da prendere in considerazione (ad esempio, il tipo di malattia), la capacità di comprendere la malattia e il significato delle cure mediche nei bambini tende a svilupparsi attraverso alcune fasi che sono abbastanza costanti per tutti (McQuaid, Howard, Kopel, Rosenblum, & Bibace, 2002; Walsh & Bibace, 1991; Wilkinson, 2006).
- La comprensione della malattia progredisce da uno stadio più infantile, immaturo, presente nei più piccoli, verso forme più complete e vicine al pensiero logico-scientifico degli adulti (Brewster, 1982; Perrin & Gerrity, 1981). Nei più piccoli questa comprensione non raggiunge ancora un livello scientifico ma è spesso collegata a eventi fantastici o magici oppure deriva da esperienze occasionali del soggetto (Bibace & Walsh, 1980; Brewster, 1982; Eiser, 1985c; Myant & Williams, 2005; Redpath & Rogers, 1984).
- Per consentire ai bambini di intendere concetti di natura medica è necessario presentarli in maniera rispondente allo stadio di sviluppo mentale che essi vivono. Non è possibile per un bambino comprendere spiegazioni che si basano su idee che richiedono strutture mentali non ancora possedute. Più nello specifico, una

comprensione della malattia o della cura che richiami il funzionamento di organi interni, la loro interazione con altri sistemi fisiologici e psicologici, secondo i modelli di matrice piagetiana risulta plausibile solo dopo gli 11-12 anni (Banks, 1990; Bombi & Cannoni, 2014; Brewster, 1982; Eiser, 1985c).

- Per questi motivi la discussione di aspetti che riguardano la malattia, le scelte terapeutiche e le conseguenze fisiche, sociali e psicologiche di queste è molto difficoltosa con bambini più piccoli di 10-11 anni e potrebbe avere effetti negativi perché non compresa in modo adeguato (Bibace & Walsh, 1980; Perrin & Gerrity, 1981).

Nella Tabella 2.6 riassumiamo le principali caratteristiche della comprensione dei bambini in base al loro stadio di sviluppo.

Tabella 2.6 *Alcuni concetti di derivazione piagetiana relativi alla comprensione della malattia nei bambini, per fasi dello sviluppo*

**Fase Preoperatoria (2-6 anni circa)**	**Fase Operatorio-concreta (7-11 anni circa)**	**Fase Operatorio-formale (11+)**
**Contagio per cause fenomeniche, causazione per vicinanza fisica.** Se il bambino si avvicina o entra in contatto con un determinato oggetto o fenomeno, può pensare che la malattia sia stata causata da questo (es. il vento tocca il tuo naso o la fronte e ti causa il raffreddore).	**Contaminazione, internalizzazione.** Se un bambino entra in contatto diretto con un dato oggetto, persona o animale può pensare che la malattia sia causata da questo contatto (es. se si accarezza un cane, poi si tocca una persona e questa sia ammala, si potrebbe pensare che la malattia sia causata da questo contatto indiretto con l'animale).	**Fisiologico e psicofisiologico.** Si comprendono cause e conseguenze della malattia, sia sul piano fisico che su quello psicologico ed emotivo.
**Causazione per vicinanza temporale.** Se la malattia compare poco dopo il verificarsi di un determinato fenomeno, il bambino potrebbe pensare che la malattia sia stata causata da quel fenomeno.	**Permane il concetto di causazione per vicinanza temporale**	**Fisicalismo.** Diverse variabili arrivano a causare la malattia attraverso interazioni che seguono una base logica.
**Egocentrismo, onnipotenza** Il bambino ritiene di essere in qualche modo lui la causa della malattia.	**Forme di distorsione egocentrica restano possibili.** Il bambino può colpevolizzarsi per essersi ammalato o per aver causato la malattia ad altri.	**Cambiamenti di ruolo.** La malattia comporta un cambiamento di ruolo e di percezione delle proprie possibilità.

**Giustizia immanente e auto colpevolizzazione.** La malattia è la conseguenza logica di una cattiva azione svolta dal bambino. Il bambino tende a colpevolizzarsi sia per le malattie venute a lui, sia per quelle che possono essere venute ad altri familiari.	**Germi:** Si tende a pensare che tutte le malattie siano causate da germi, che si trasferiscono e infettano altre persone.	**Germi:** Malattie diverse sono causate da patogeni diversi; non tutte le malattie hanno origine batterica o virale. Si comprende il fatto che gli agenti infettivi sono essi stessi esseri viventi che possono quindi diffondersi nell'organismo e riprodursi

2.2 Verso il superamento delle teorie stadiali

Le ricerche che abbiamo qui elencato, e che hanno assunto la teoria stadiale di Piaget come modello teorico di riferimento, non hanno in realtà indagato le potenzialità di comprensione e di apprendimento dei bambini malati, ma si sono limitate a documentare il loro stadio evolutivo-concettuale rispetto a determinati problemi connessi alla malattia. Esse inoltre si concentrano in modo quasi esclusivo su aspetti cognitivi. Così facendo queste ricerche ricalcano però un modo di pensare adulto-centrico: si basano sull'assunto che una comprensione più completa della malattia debba necessariamente rispondere a criteri scientifici, logici e formali. L'aspetto cruciale della cultura e della vita infantile, cioè il mondo dei vissuti emotivi affettivi, della capacità di giocare, di vedere amici e fare cose assieme, appare del tutto trascurato da questo tipo di studi (Capurso, Bianchi di Castelbianco, & Di Renzo, 2020; Hill & Tisdall, 2014; James, Jenks, & Prout, 1998). Come vedremo più avanti, questi elementi giocano un ruolo fondamentale anche nei confronti delle possibilità di comprendere ed elaborare adeguatamente i vissuti di malattia nei bambini.

Nel campo della psicologia dell'educazione ci si muove verso un graduale superamento delle idee piagetiane. La stessa teoria stadiale di Piaget, pur presentando un caposaldo della psicologia dello sviluppo del XX secolo, ha comunque subito, nel corso del tempo, numerose critiche e revisioni (Carugati & Selleri, 2005; Ligorio & Cacciamani, 2013). Oggi si crede sempre di meno che la capacità di comprensione di un bambino sia limitata da una struttura cognitiva che appare rigidamente legata alla sua età. In effetti, non è dimostrato che all'interno di uno stesso stadio le capacità di comprendere qualsiasi tipo di fenomeno siano analoghe, indipendentemente dal contesto e dall'esperienza. Al contrario, si hanno prove che contesti e situazioni diverse possano condurre il soggetto ad esprimere abilità differenti anche nello stesso tipo di compito (Cole, 1998).

Molti autori hanno provato come le situazioni sperimentali che Piaget presentava ai bambini fossero in realtà troppo lontane dalla loro esperienza quotidiana e troppo complesse. Esperimenti analoghi a quelli piagetiani, proposti in contesti diversi

e più familiari, proverebbero che in realtà i bambini sarebbero capaci di giungere a comprendere correttamente situazioni molto più complesse di quanto lo stesso Piaget credesse. Questo avviene se si predispongono determinate condizioni facilitanti (per esempio, una guida verbale fornita da un adulto) o se si propongono prove più vicine alla esperienza quotidiana dei soggetti coinvolti (Carey, 1987; Donaldson, 1979; Meadows, 2012; Sharp, 1982; Siegler, 1976; Sperber, Premack, & Premack, 1995; D. Wood, 1998).

Tutto questo ha ripercussioni anche dal punto di vista della comprensione della malattia. Gli studi di matrice piagetiana, infatti, sembrano evidenziare ciò che il bambino sa e non sa ad un dato stadio del proprio sviluppo (Rushforth, 1999), e si assume che ciò che il bambino sa rappresenti anche il *limite* dei suoi potenziali di apprendimento e comprensione. Ne consegue che questo approccio porta a credere che un bambino di una certa età non possieda determinate strutture concettuali e pertanto non possa comprendere adeguatamente alcuni aspetti legati alla sua malattia. Come nota Rushforth (1999), invece, anche i bambini più piccoli possono raggiungere livelli più avanzati di comprensione della malattia, purché ci si preoccupi di comunicare con loro in un modo calibrato in base alle loro potenzialità di apprendimento e, aggiungiamo noi, con una modalità coerente con il loro sistema socio-culturale di riferimento. Sulla base di queste considerazioni, l'aspetto che più dovrebbe interessare l'educatore è ciò che il bambino *può arrivare a capire*, quali traguardi conoscitivi egli può raggiungere, come partecipare e sostenerlo in questo suo percorso di ricerca di significato.

Nelle prossime pagine presentiamo alcune delle teorie e linee di pensiero che ci consentono di superare l'ipotesi maturativa di Piaget, per analizzare poi come queste possano applicarsi nella comunicazione e spiegazione della malattia. Parliamo dunque di Costruttivismo sociale, Zona di sviluppo prossimale, Scaffolding e Pensiero narrativo.

2.2.1 Il Socio-costruttivismo

A partire dagli anni Ottanta del secolo scorso è emerso un modello di sviluppo cognitivo più dinamico e flessibile di quello piagetiano, maggiormente ancorato alle dimensioni sociali e culturali del contesto di vita di bambino. Questo modello non nega alcuni aspetti cruciali della teoria di Piaget, ma li reinterpreta in modo meno rigido e assoluto, collegando le esperienze personali del bambino alle opportunità di sostegno e alle facilitazioni nella realizzazione dei potenziali evolutivi che l'ambiente sociale e culturale possono offrire (Carugati & Selleri, 2005). Autori come Vygotskij (1978), Michael Cole (1998), Bruner (1991), e Bronfenbrenner (2005) hanno fornito importanti contributi teorici e di ricerca che dimostrano come il forte collegamento che esiste tra le dimensioni sociali, culturali, emotive del bambino e le sue esperienze cognitive, contribuisca a rendere il soggetto in via di sviluppo un costruttore attivo di conoscenze e di significati della realtà. La caratteristica principale di queste teorie è quella di enfatizzare il bambino-nel-contesto, evidenziando il fatto che i bambini sono attivi costruttori

di conoscenza grazie alla loro relazione attiva con la cultura di apparenza e il contesto sociale e ambientale ove si trovano a crescere.

L'apprendimento non viene più visto solo come un processo individuale, ma viene anzitutto costruito attraverso le interazioni con gli altri, che avvengono sempre in uno specifico contesto socio-culturale (Carugati & Selleri, 2005). Il focus dell'attenzione si sposta quindi dall'interazione individuo- ambiente fisico e naturale all'interazione sociale all'interno di un gruppo. La costruzione dei significati avviene ora grazie all'interazione con altri e alla condivisione e negoziazione delle proprie idee, piuttosto che tramite un processo di interiorizzazione di conoscenze che esistono esternamente all'allievo (Ligorio & Cacciamani, 2013).

2.2.2 La Zona di sviluppo prossimale

Un altro contributo importante al costruttivismo è rintracciabile nel concetto di zona di sviluppo prossimale di Vygotskij (1934). Secondo l'autore è necessario distinguere tra due tipi di apprendimento. Un primo tipo è identificabile con lo sviluppo effettivo. Esso è misurabile con test, esami e prove individuali. Si tratta di vedere, in un dato momento, cosa un bambino sa di un dato argomento, quali problemi è in grado di risolvere da solo. Lo sviluppo effettivo è indice di un percorso pregresso che quella persona ha svolto, rappresenta il bagaglio di competenze che un bambino ha accumulato e interiorizzato in modo stabile nel corso del tempo.

Tuttavia, per Vygotskij l'apprendimento segue una strada che va dall'esterno verso l'interno. Ogni competenza, ogni capacità, prima di venire interiorizzata è espressa da chi apprende come forma socializzata di sapere. In altre parole, prima di poter fare delle cose da solo, un bambino è in grado di farle assieme ad altri, soprattutto se sostenuto dall'interazione con una persona più competente.

Nasce così il concetto di zona di sviluppo prossimale, che costituisce il secondo tipo di apprendimento. Essa rappresenta quello spazio di competenze e abilità che un bambino può mettere in atto grazie all'interazione con altre persone più competenti di lui. Si tratta di uno spazio potenziale, che può concretizzarsi solo se si presentano le condizioni socio-relazionali adatte. Secondo Vygotskij l'apprendimento è massimamente efficace se si opera all'interno della zona di sviluppo prossimale, perché questo consentirà al bambino di esprimere socialmente nuove competenze così da poterle poi interiorizzare in maniera stabile.

In sintesi, mentre lo sviluppo effettivo misura ciò che il bambino già sa, lo sviluppo prossimale opera nell'area di quelle nuove conoscenze e abilità che il bambino può arrivare a dominare ed esercitare.

2.2.3 Lo Scaffolding

Lo scaffolding, cioè l'"impalcatura" (David Wood, Bruner, & Ross, 1976), è una metafora usata da Bruner e Wood per indicare le diverse funzioni del tutoring nella soluzione di problemi. Nel caso specifico gli autori esaminano l'interazione all'interno

della zona di sviluppo prossimale di un tutor adulto con bambini di 3, 4 e 5 anni cui viene chiesto di costruire una piramide con dei cubetti di legno ad incastro. Al tutor è stato richiesto di mettere in grado ogni bambino di fare il massimo possibile da solo. L'interazione tra tutor e bambino è stata videoregistrata e analizzata in termini di iniziativa dei singoli, tipologia dei suggerimenti forniti dal tutor e comprensione delle consegne da parte dei bambini. Gli autori trovano che le interazioni tutor-allievo si configurano in modo diverso a seconda delle diverse fasce d'età, e concludono indicando sei funzioni dello scaffolding (coinvolgimento inziale del soggetto nel compito; riduzione del grado di libertà di scelte possibili, al fine di semplificare le scelte da compiere; mantenimento della direzione per orientare il bambino verso il raggiungimento dell'obiettivo; segnalazione dei momenti critici per richiamare la massima attenzione dell'allievo; controllo della frustrazione davanti agli errori; Dimostrazione o modellamento; David Wood et al., 1976).

Attraverso lo scaffolding un bambino che apprende concetti nuovi o che risolve un problema a lui sconosciuto viene sostenuto e accompagnato attraverso la sua zona di sviluppo prossimale verso la soluzione, attraverso un appoggio offerto dalla persona più competente. Questo sostegno si renderà via via più tenue, fino alla completa autonomia.

2.2.4 Il Pensiero narrativo

Il pensiero narrativo, nell'accezione di Bruner (2009), si differenzia dal pensiero paradigmatico o logico-scientifico in quanto si basa su un diverso modo di ordinare e di elaborare i dati dell'esperienza. I due tipi di pensiero sono tra loro irriducibili e si pongono obiettivi differenti utilizzando principi operativi e procedure di verifica totalmente diverse. Un'argomentazione, frutto del pensiero paradigmatico, tende a "convincere" della verità attraverso categorizzazioni e concettualizzazioni, mentre il racconto, prodotto del pensiero narrativo, si basa fondamentalmente sulla legge della verosimiglianza. "Il pensiero narrativo" dice Bruner "si occupa delle intenzioni e delle azioni proprie dell'uomo o a lui affini, nonché delle vicissitudini e dei risultati che ne contrassegnano il corso. Il suo intento è quello di calare i propri prodigi atemporali entro le particolarità dell'esperienza e di situare l'esperienza nel tempo e nello spazio" (Bruner, 2009). Una delle caratteristiche della narrazione è la sua parziale indeterminazione. Spesso nei racconti le cose sono sfumate e proprio queste sfumature aprono gli spazi dell'immaginazione della fantasia del lettore. È proprio per questo motivo che il racconto può suscitare nell'ascoltatore reazioni differenti ed è per questo che può, in alcuni contesti, risultare molto più efficace di una buona argomentazione. "Un atto linguistico narrativo" dice ancora Bruner "sia esso *concluso* o *in corso*, produce un mondo al congiuntivo". (Bruner, 2009). Un mondo al congiuntivo è, in questa accezione, un luogo dove non esistono necessariamente certezze, ma dove possono convivere possibilità differenti e dove possono esprimersi desideri, previsioni e ipotesi.

Il pensiero narrativo produce quindi rappresentazioni più che descrizioni della re-

altà e si occupa fondamentalmente della dimensione psichica di colui che racconta e di colui che ascolta evocando, grazie all'uso di analogie, immagini e sentimenti in entrambi.

2.2.5 La costruzione sociale di significati nel sistema bioecologico

Anche le teorie sistemiche contribuiscono con alcune idee al concetto di co-costruzione dei significati da parte dei soggetti in via di sviluppo. Ad esempio, secondo il modello bioecologico di Bronfenbrenner (1986, 2010), le capacità umane e la loro progressiva evoluzione dipendono in modo significativo dal più ampio contesto sociale e istituzionale in cui si svolge l'attività individuale e pertanto non sono riconducibili a singoli elementi secondo un rapporto lineare di causa ed effetto. Nell'ipotesi dell'autore ambiente, gruppo, sviluppo individuale e apprendimento sono strettamente correlati e interdipendenti e si configurano come "...un insieme di strutture incluse l'una nell'altra, simili ad una serie di bambole russe" (Bronfenbrenner, 1986, p.31). Dunque l'influenza della maturazione biologica rappresenta solo uno dei fattori che rendono possibile al bambino la comprensione degli eventi legati alla malattia. Altri elementi determinati, secondo Bronfenbrenner, sono la cultura, le relazioni interpersonali, i ruoli sociali, i processi di costruzione di senso.

Un secondo concetto importante riguarda l'interazione dinamica (e non una causalità lineare) che collega questi elementi (Ford & Lerner, 1992). Questo significa che qualsiasi parte dell'ecosistema umano risulta reciprocamente legata alle altre e ogni sua modificazione richiede l'aggiustamento di altri elementi. Questo vale anche per il soggetto in via di sviluppo, che, proprio mentre cresce per l'effetto degli eventi che avvengono nel proprio ambiente di vita, modifica l'ambiente stesso, causando a sua volta dei mutamenti ambientali. Se pensiamo, ad esempio, ad una classe, è vero che le azioni dell'insegnante avranno conseguenze sull'apprendimento degli studenti; ma, allo stesso tempo, anche le azioni degli studenti creeranno degli apprendimenti nell'insegnante. Inoltre ogni cambiamento di un singolo bambino potrà avere dei riflessi sui suoi compagni. Se applichiamo tutto questo alla comprensione della malattia, possiamo facilmente comprendere come tale comprensione sia legata ad un'ampia rete relazionale che parte dal livello di sviluppo del soggetto ma si estende, influenza ed è influenzata da un'ampia rete di fattori biologici, ecologici, socio-culturali.

2.2.6 Esempi di costruzione sociale e di narrazione connessi alla malattia nei bambini

La zona di sviluppo prossimale e lo scaffolding operano anche nel caso della comprensione della malattia. Capurso (2007, pp. 55-57) riporta l'esempio di due bambini di età diversa, Federico di 11 anni e Alessandro di 8 (Fig. 2.3), che hanno disegnato la leucemia lavorando nella stessa stanza e nello stesso momento, essendo ricoverati nello stesso reparto.

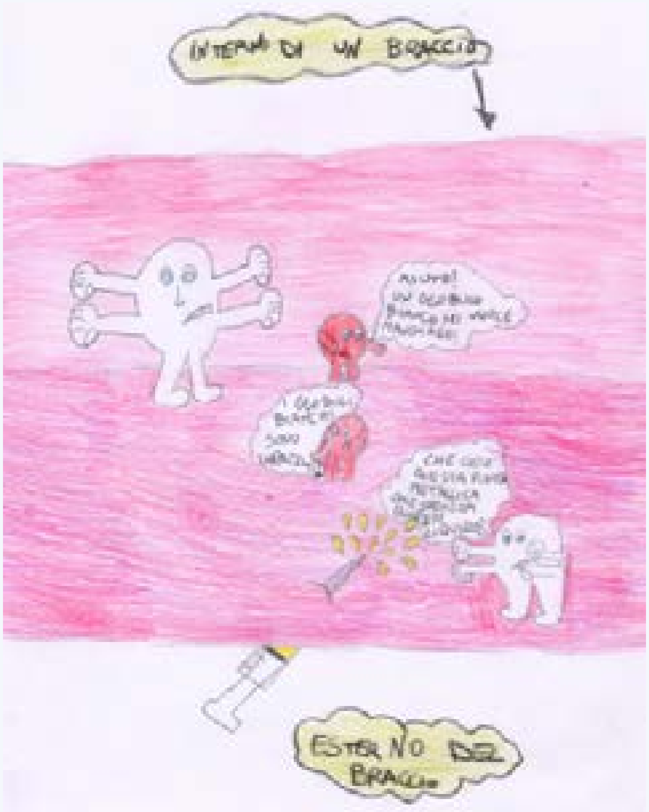

Federico, 11 anni

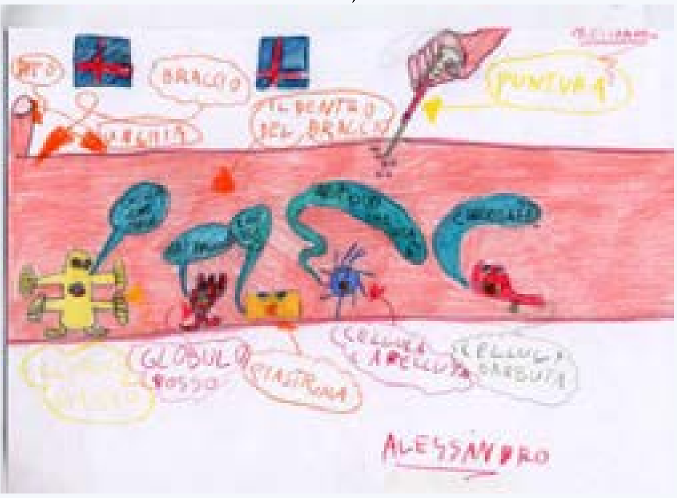

Alessandro, 8 anni

Figura 2.2 *Un esempio di comprensione della malattia basato sulla zona di sviluppo prossimale. Il bambino più piccolo (Alessandro, 8 anni) elabora una comprensione più avanzata grazie all'interazione con Federico (11 anni).*

I disegni prodotti dai due bambini sono molto simili e anche quello del bambino più piccolo arriva ad identificare elementi interni al sangue, invisibili, che sono però collegati alla sua malattia e indica l'interazione di questi elementi con il medicinale come possibile cura. Capurso conclude che questa dimensione di scambio sociale della co-

noscenza ha probabilmente condotto anche il più piccolo ad esprimere idee e concetti che molti autori piagetiani non avrebbero ritenuto possibili a 8 anni di età.

In termini pedagogici, il significato profondo del concetto di zona di sviluppo prossimale è questo: per un bambino la possibilità di apprendere e di capire non dipenderebbe soltanto da elementi maturativi interni e dalle sue esperienze pregresse, quanto piuttosto dalla capacità dell'ambiente sociale di fornirgli quegli strumenti simbolici-culturali di cui egli ha bisogno per progredire (Dixon-Krauss, 1998). Questo avviene quando a quel bambino viene data la possibilità di entrare attivamente in relazione con qualcuno più capace o più informato di lui, che gli consenta, appunto, di operare nella sua zona di sviluppo prossimale, proprio come è avvenuto nell'esempio di Federico.

Attraverso l'utilizzazione del pensiero narrativo il bambino può invece entrare in rapporto con quella complessità psichica altrimenti inesprimibile e può crearsi un'immagine della realtà, pur in assenza di logiche concatenazioni di elementi. In questo senso il pensiero narrativo costituisce una grande risorsa promotrice sia di nuovi apprendimenti sia di comportamenti adattavi e come tale può risultare molto utile a bambini malati.

Giovanna (Figura 2.4) ci propone un chiaro esempio delle funzioni di rielaborazione rese possibili dall'espressione del proprio pensiero narrativo. La bambina deve operarsi, è in ospedale in attesa dell'intervento e ha tanta paura. Rappresenta la sala operatoria come una grande stanza nella quale prevale un freddo colore blu. La bambina si immagina già sdraiata sul tavolo e comunica la propria paura di provare dolore il giorno dopo. La parte più interessante del lavoro è la nota con cui Giovanna ha spiegato il proprio lavoro. Questa si conclude con la consapevolezza che la bambina ci comunica con l'ultima frase: aver parlato dei propri timori è stato di aiuto per vincere la paura.

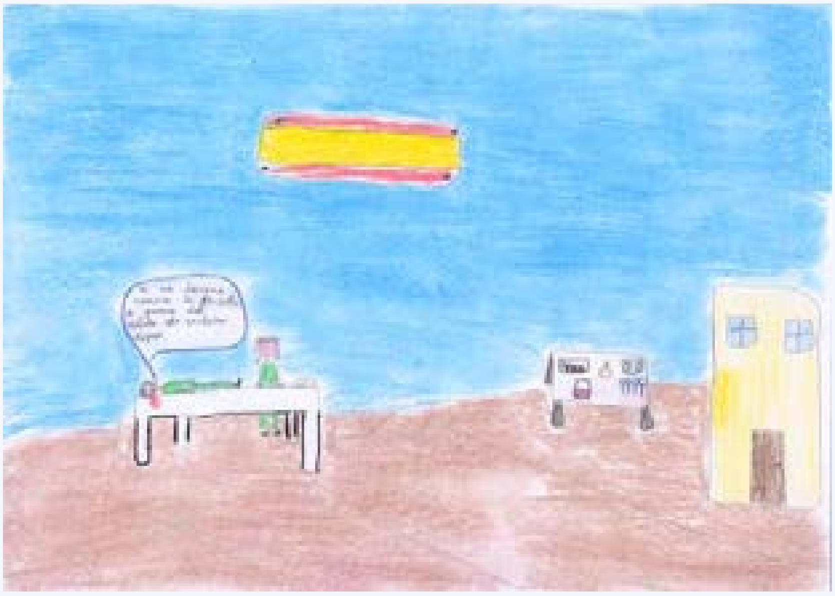

Figura 2.4. *Un esempio di pensiero narrativo riferito al vissuto di malattia*

Io ho tanto paura di operarmi ma lo devo fare.

Io mi devo operare le tonsille e una parte del naso.

Ho immaginato l'operazione disegnando la sala operatoria con il disegno.

Ho disegnato il letto, la luce, la porta e il tavolo dove stanno i ferri e le cose per operare.

Ho immaginato come dovranno operarmi e adesso visto che ne ho parlato mi è passata la paura (Giovanna, 8 Anni).

Fonte: (Capurso, 2007, p. 37)

Grazie alla sua possibilità di muoversi su un doppio scenario il racconto produce infatti una polisemia che rimanda costantemente ad altri mondi, pur ancorandosi ai dati oggettivi del reale (Sarmento, 2006). Quando un bambino disegna, produce un racconto che da una parte lo colloca nello spazio del foglio; dall'altro lo collega alle condizioni culturali e al contesto specifico della realtà esterna rappresentata; infine lo proietta nel mondo interno con l'intensità delle emozioni che lo contraddistinguono. Il racconto che ne emerge è quindi solo in parte descrittivo dell'avvenimento disegnato, perché l'elemento profondamente soggettivo dà il via a una trama più articolata che non era prevedibile all'inizio della rappresentazione grafica (Di Renzo, Marini, & di Castelbianco, 2013).

Nella proposta didattica che proponiamo a supporto del rientro dei bambini in classe, abbiamo inserito diverse attività che facilitano i bambini nella produzione di contenuti narrativi, sia verbali che pittorici. È dunque importante affiancare sempre al lavoro sulle schede momenti di condivisione dei contenuti prodotti a coppie, piccoli gruppi o di classe.

Un altro modo per indagare, tramite il pensiero narrativo, i vissuti di malattia nei bambini può essere quello di invitarli a creare delle metafore (Cameron, 1996). Creare metafore non è facile per un bambino, ma quando lo fa i risultati sono molto evocativi e profondi.

Giulio (Fig. 2.5) rappresenta la sua malattia oncologica come "un mostro orribile" e molto possente.

L'animale viene tratteggiato con colori forti, aggressivi e appare come la figura predominante nel foglio. È dotato di braccia tentacolari e di un ghigno minaccioso. Di fronte ad una figura di questo tipo chiunque si sentirebbe intimorito. Eppure Giulio vince la sua paura. Non fugge in preda al panico o alla disperazione, ma, pur rappresentandosi senza capelli e con la mascherina, aspetta il mostro per affrontarlo a spada tratta con il corpo piegato verso di lui in attesa del combattimento.

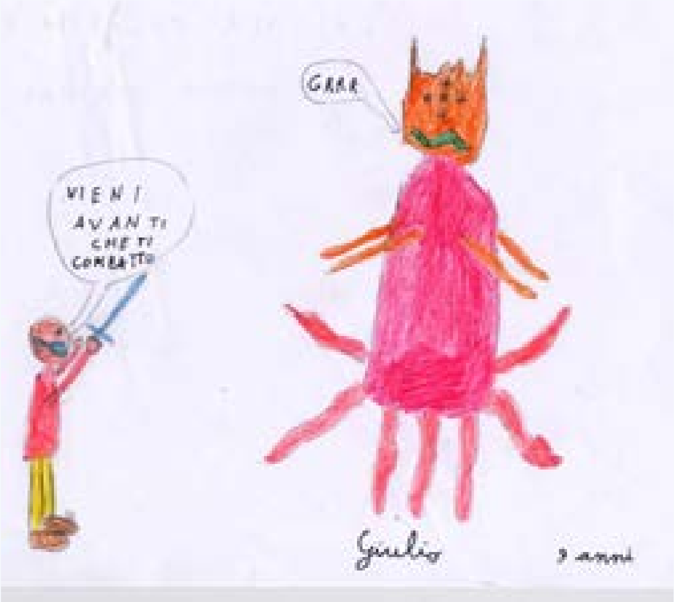

Figura 2.5 *Un esempio di rappresentazione della malattia come metafora. Il mostro.*

La mia malattia è come un mostro orribile. Giulio, 9 anni.
Fonte: (Capurso, 2007, p. 37)

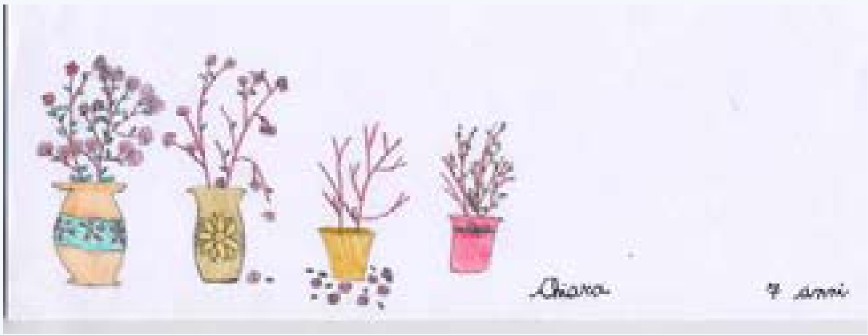

Figura 2.6 *Un esempio di rappresentazione della malattia come metafora. La pianta fiorita.*

La mia malattia è come un fiore che sfiorisce e poi rinasce. Chiara, 7 anni.
Fonte: (Capurso, 2007, p. 37)

La metafora di Chiara (Fig. 2.6) è invece meno cruenta e molto più aperta al futuro. La bambina vede la sua malattia (ma è più probabile che stia parlando di se stessa) come "un fiore che sfiorisce e poi rinasce". Il disegno è ricco di elementi cromatici e di speranza, visto che l'ultima pianta sta mettendo dei germogli che rimandano alla vita che ritorna. Il futuro è ancora incerto, dato che la parte destra del foglio è bianca, ma è proprio lì che la bimba ha messo la sua firma, quasi a rassicurarci (e rassicurarsi) sulla sua presenza in futuro.

2.3 Il bambino competente e la comprensione della malattia

Abbiamo visto come diverse teorie della psicologia dell'educazione e dello sviluppo indichino come il bambino sia in grado di esprimere competenze e comprendere concetti nuovi ben al di là del suo livello di sviluppo cognitivo. È quindi il caso di chiedersi se e come questa immagine di "bambino competente" possa applicarsi anche al campo della comunicazione e comprensione della malattia (Hart & Rollins, 2011).

In effetti questa tendenza ad attribuire al bambino maggiori competenze rispetto a quelle assegnategli dai modelli piagetiani ha anche influenzato un ampio corpus di ricerche relative alla comprensione della malattia nei bambini (per una review completa, si veda Bianchi di Castelbianco et al., 2007).

Uno dei primi ricercatori a mettere in discussione l'ipotesi maturazionista di Bibace e Walsh è stato Michael Siegal (Siegal, 1988; Siegal & Peterson, 2005). Nel suo studio del 1988 l'autore ha creato un set di tre situazioni sperimentali per verificare la capacità dei bambini di comprendere il concetto di contagio e contaminazione quali cause di alcune malattie, studiando 120 bambini divisi in diverse fasce d'età, dalla scuola dell'infanzia alla terza elementare. Ai bambini sono stati mostrati filmati e riferiti racconti e poi veniva loro chiesto se le affermazioni dei vari protagonisti fossero vere o sbagliate

e che cosa nella realtà si dovesse fare per evitare di ammalarsi. Le storie riguardavano personaggi con il raffreddore, con il mal di denti, con un ginocchio ferito. Altre storie erano invece riferite al concetto di contaminazione e mostravano un bicchiere di latte nel quale cadeva un pettine, un insetto o un cucchiaino. Ai bambini si chiedeva se fosse più o meno pericoloso bere quel latte.

Anche i bambini di scuola materna hanno saputo indicare, nella maggioranza dei casi, le risposte corrette, distinguendo il contagio dalla giustizia immanente ed escludendo comunque che il mal di denti fosse contagioso. I risultati ottenuti da Siegal indicano che i modelli piagetiani possono portare a sottostimare la capacità di comprensione dei bambini. Siegal conclude che anche bambini di scuola dell'infanzia possiedono conoscenze relative al contagio quale possibile causa di alcune malattie, specialmente se essi hanno avuto esperienza diretta di quella malattia. Anche i più piccoli sono quindi in grado di comprendere spiegazioni e di attuare comportamenti di auto-tutela, se questi vengono loro spiegati e se mettono in evidenza le cause di una malattia.

Anche Hergenrather and Rabinowitz (1991) muovono alcune critiche all'applicazione della teoria stadiale alla comprensione della malattia. Analizzando le spiegazioni e i racconti di un gruppo di bambini in età scolare, gli autori ritengono che il concetto di malattia si evolva attraverso alcuni schemi che cambiano nel corso del processo di sviluppo in base alle esperienze individuali.

Nei bambini più piccoli e quindi con meno esperienza la malattia viene compresa in termini di azioni umane (si è malati perché la mamma ti prova la febbre). Essi riconoscono la malattia in base alle conseguenze che questa ha sui comportamenti e sugli eventi che si verificano intorno a loro. Anche la terapia viene compresa in termini comportamentali e la guarigione sarebbe una sorta di processo spontaneo. Con l'aumento delle conoscenze e delle esperienze dirette emerge il concetto di contagio. Questo concetto si sviluppa e differenzia fino ad includere le infezioni microbiche, i comportamenti imprudenti, la genetica e i processi interni di natura psicobiologica. La comprensione del processo di cura comprende le funzioni delle medicine e il ruolo attivo dell'organismo che riesce a reagire agli agenti patogeni. Nella loro ricerca gli autori indagano l'idea di cura e malattia creando alcune prove di organizzazione e di classificazione di carte e vignette e scoprono che bambini della stessa età possono ricorrere a schemi e criteri diversi. Essi concludono che la comprensione della malattia non dipende tanto dall'età quanto dal sistema di conoscenze precedentemente accumulate e dunque dalla natura degli schemi che si sono sviluppati. I bambini più piccoli possiedono una conoscenza della malattia, delle sue conseguenze e delle terapie che, per quanto incompleta, è molto più avanzata di quanto supponessero gli studi piagetiani.

Anche il lavoro di Kalish (1996) si pone in analogia con questa linea di pensiero. L'autore ipotizza che il concetto di malattia si costruisca sulla base di un cumulo di proprietà (*property cluster*). Non esiste quindi una definizione di malattia, ma, piuttosto, un insieme di caratteristiche, azioni, comportamenti che i bambini, sulla base della propria esperienza, collegano all'essere malati (es. misurare la febbre, stare a let-

to, prendere medicine, avere determinati sintomi, ecc.). È la presenza o assenza delle proprietà del concetto di "essere malati" a definire lo stato di salute o malattia del soggetto. Per i bambini le proprietà principali dello stato di malattia sono l'avere la febbre (o altri sintomi), il prendere medicine, dover stare a letto e non poter giocare.

In Tabella 2.7 riportiamo alcuni esempi degli studi post piagetiani più significativi nel campo della comprensione della malattia nei bambini.

Tabella 2.7 *Alcuni studi sulla comprensione della malattia basati sul concetto di bambino competente*

Autore	Argomento della ricerca	Indicazioni
(Siegal, 1988)	Analisi dei concetti di contagio e contaminazione in bambini di diverse età.	Viene contraddetta l'ipotesi che bambini più piccoli non sono in grado di comprendere le cause di una malattia (Bibace e Walsh, 1981). Si suggerisce, al contrario, che la comprensione delle cause di una malattia contagiosa è alla portata dei bambini.
(Eiser, 1989)	Lo sviluppo del concetto di malattia nei bambini: verso un superamento delle teorie stadiali.	Rivede la propria posizione piagetiana e riconosce che interventi educativi (e non la pura maturazione fisiologica) possono modificare la capacità di comprensione dei bambini
(Hergenrather & Rabinowitz, 1991)	Analisi dell'organizzazione del pensiero e dello sviluppo dei concetti di salute e malattia in bambini di diversa età.	Ricerca chiave che dimostra come le capacita di comprensione della salute e della malattia dei bambini più piccoli siano maggiori di quanto non si credesse in passato. Gli autori ritengono che sia scorretto riferirsi agli stadi maturazionali di Piaget in quanto questi si riferiscono a specifiche forme logiche di pensiero causale, ma non alla capacità di comprensione dei bambini.
(Hauck, 1991)	Analisi delle abilità cognitive di bambini di scuola dell'infanzia.	Evidenzia come la tendenza del personale medico di basarsi sulle teorie stadiali di Piaget nello spiegare la malattia ai bambini sia insufficiente alla luce di nuove teorie di psicologia dello sviluppo. Propone che un modello cognitivo basato sull'information processing possa consentire una comunicazione più adeguata.
(Springer & Ruckel, 1992)	Le credenze dei bambini sullo sviluppo delle malattie: evidenze contrarie al concetto della giustizia immanente.	Gli autori contrastano l'idea che la giustizia immanente costituisca una spiegazione molto presente nei bambini di scuola dell'infanzia, provando che anche bambini più piccoli possono ricorrere al concetto di contaminazione e all'idea che siano i germi a causare determinate malattie.
(Yoos, 1993)	Nuovi e vecchi paradigmi nello sviluppo dei concetti di malattia nei bambini.	L'articolo passa in rassegna e critica le teorie piagetiane e propone modelli alternativi per spiegare la malattia ai bambini.

(Kalish, 1996)	Cause e sintomi nei concetti di malattia in bambini di età prescolare.	Il concetto di malattia è costruito sulla base di un cumulo di proprietà. Il concetto di essere malati sarebbe costituito da un gran numero di elementi, azioni, comportamenti associati alla malattia.
(Raman & Winer, 2002)	Analisi della coesistenza di forme di pensiero diverse in bambini e adulti.	È possibile che diverse forme di pensiero coesistano. Le tipologie di spiegazione della malattia tendono a variare con l'età, ma sono possibili forme di mobilità stadiale. Il contesto e il modo di porre quesiti possono determinare risposte molto diverse. Persino negli adulti permangono forme di giustizia immanente.
(Cannoni & Norcia, 2008)	L'immagine di sé nella salute e nella malattia: uno studio empirico in bambini dai 6 agli 11 anni	Lo studio esplora l'immagine di sé quando si è sani e quando si è malati in 130 bambini di 6-11 anni d'età. Il "sé malato" viene rappresentato prevalentemente in una stanza, immobile e triste, mente il "sé sano", è stato più spesso disegnato all'aria aperta, mentre gioca o cammina, sorridente. Il contrasto tra le due situazioni aumenta con l'età. I bambini che hanno avuto esperienze di ospedalizzazione valorizzano maggiormente il sé malato rispetto a bambini che non hanno avuto tale esperienza.
(Varkula, Resler, Schulze, & McCue, 2009)	Analisi dell'influenza delle comunicazioni genitoriali e delle esperienze di vita nella comprensione di malattie tumorali nei bambini.	Questo studio che ha coinvolto 25 bambini di 4-5 anni e i rispettivi genitori, rivela che già a questa età i bambini sanno associare il cancro ad una malattia. I genitori che avevano avuto modo di parlare con i propri figli del cancro avevano figli che risultavano più esperti della malattia rispetto ai coetanei i cui genitori non ne avevano mai parlato.
(Zhu, Liu, & Tardif, 2009)	La spiegazione della malattia in bambini cinesi.	Questo studio ha analizzato le spiegazioni spontanee dei concetti legati alla malattia in un campione di 60 bambini cinesi di età compresa tra i 3 e i 5 anni e di diverso status socioeconomico. Le risposte dei partecipanti alla domanda "perché la gente si ammala?" sono state codificate in base a 5 categorie: psico-biologica, biologica, comportamentale, sintomatica, altro. La maggior parte delle risposte dei bambini ha riguardato la categoria comportamentale e sintomatica. Le risposte di tipo biologico tendevano ad aumentare al crescere dell'età. Secondo gli autori alcune delle riposte fornite dai bambini sembravano inoltre ricollegarsi ad esperienze culturali e alla medicina tradizionale cinese.
(McIntosh & Stephens, 2012; McIntosh, Stephens, & Lyons, 2013)	Analisi dello sviluppo del concetto di causalità della malattia e dell'influenza del contesto familiare nella creazione di tale concetto.	La comprensione della malattia nei bambini viene analizzata partendo dal contesto socio-culturale della famiglia. I risultati di queste ricerche confermano l'importanza dell'influenza di questo contesto nel determinare le capacità dei bambini di comprendere i meccanismi di malattia e nell'apprendere comportamenti di prevenzione.

(Leonhardt, Margraf-Stiksrud, Badners, Szerencsi, & Maier, 2014)	Effetti del progetto "ospedale dei pupazzi" sulla comprensione delle azioni di cura in bambini prescolari.	Questo studio analizza gli effetti del progetto internazionale denominato "Ospedale dei Pupazzi" (per l'Italia si veda: http://www.nazionale.sism.org/ospedale-dei-pupazzi/) sulle conoscenze mediche dei bambini che vi hanno partecipato. I risultai dimostrano che i bambini che hanno preso parte al progetto hanno significativamente migliorato le loro conoscenze per quanto riguarda il funzionamento corporeo, la salute e la malattia.
(Capurso et al., 2016)	Costruire la spiegazione dell'Artrite idiopatica giovanile partendo da vissuti e narrazioni di bambini già malati.	Adottando un approccio socio-costruttivista e coinvolgendo bambini di età diversa è possibile creare una spiegazione della malattia a partire dalle narrazioni dei bambini malati.

2.4 Cosa possiamo concludere

Quando parlano della malattia i bambini sanno esprimere conoscenze e competenze molto diverse. Se si creano le condizioni cognitive, sociali e relazionali più opportune, la loro comprensione può raggiungere livelli anche molto complessi. Una delle chiavi per favorire questo processo maturativo è la creazione di un sistema di narrazioni condivise in gruppo. La narrazione non è solo un momento "passivo", nel quale si riferisce ad altri un pensiero, ma è, al contrario, momento fondamentale in cui questo pensiero si *costruisce attivamente*, anche grazie all'interazione di altri e di un adulto più competente. Nel caso della recente emergenza causata dal nuovo coronavirus in Italia, è chiaro che la scuola possa e debba proporsi come luogo di contenimento emotivo, contesto nel quale sia possibile raccontare quello che si è provato, ascoltare le narrazioni degli altri, e costruire dunque assieme un senso degli eventi passati e futuri che consenta ad alunni e insegnanti di riprendere e il percorso didattico e di re-indirizzare le loro risorse emotive e cognitive verso la ripresa di quei processi evolutivi che in questi mesi sono stati interrotti. Nel capitolo terzo di questo testo illustriamo quindi le dinamiche psichiche che si attivano nei bambini e ragazzi quando essi si trovano a confrontarsi con situazioni stressanti, per poi passare, con le due appendici, a suggerire percorsi didattici da far svolgere in classe.

Bibliografia del capitolo

ANSA. (2020). Coronavirus: Lenzuola #andratuttobene con l'arcobaleno sui balconi [Press release]. Retrieved from http://www.ansa.it/sito/notizie/cronaca/2020/03/11/andratuttobene-arcobaleno-sui-balconi_ac60cbc4-38cb-4bd1-87acba36b6c750fd.html

Aptekar, L., & Boore, J. A. (1990). The emotional effects of disaster on children: A review of the literature. *International Journal of Mental Health, 19*(2), 77-90.

Banks, E. (1990). Concepts of Health and Sickness of Preschool and School-Aged Children. *Children's Health Care, 19*(1), 43-48. doi:10.1207/s15326888chc1901_6

Bianchi di Castelbianco, F., Capurso, M., & Di Renzo, M. (2007). *Ti racconto il mio ospedale: esprimere e comprendere il vissuto della malattia*. Roma: Magi.

Bibace, R., & Walsh, M. E. (1980). Development of children's concepts of illness. *Pediatrics, 66*(6), 912-917.

Bibace, R., & Walsh, M. E. (1981). *Children's conceptions of health, illness, and bodily functions*. San Francisco: Jossey-Bass.

Bombi, A. S., & Cannoni, E. (2014). *Bambini e salute. Psicologia dello sviluppo per le professioni sanitarie*: Il Mulino.

Brewster, A. B. (1982). Chronically III Hospitalized Children's Concepts of Their Illness. *Pediatrics, 69*(3), 355-362. Retrieved from http://pediatrics.aappublications.org/content/69/3/355.abstract

Bronfenbrenner, U. (1986). *Ecologia dello sviluppo umano*. Bologna: Il mulino.

Bronfenbrenner, U. (2005). *Making human beings human: bioecological perspectives on human development*. Thousand Oaks: Sage Publications.

Bronfenbrenner, U. (2010). *Rendere umani gli esseri umani: bioecologia dello sviluppo* (M. Capurso, Trans.). Trento: Erickson.

Bruner, J. S. (1991). The Narrative Construction of Reality. *Critical Inquiry, 18*(1), 1-21. doi:10.2307/1343711

Bruner, J. S. (2009). *La mente a più dimensioni* (2 ed.). Roma; Bari: GLF editori Laterza.

Cameron, L. (1996). Discourse Context and the Development of Metaphor in Children. *Current Issues In Language and Society, 3*(1), 49-64. doi:10.1080/13520529609615452

Cannoni, E., & Norcia, A. D. (2008). L'immagine di sé nella salute e nella malattia: uno studio empirico in bambini dai 6 agli 11 anni. *Rassegna di Psicologia, 25*(3), 67-60.

Capurso, M. (2001). *Gioco e studio in ospedale: creare e gestire un servizio ludico-educativo in un reparto pediatrico*. Spini di Gardolo: Erickson.

Capurso, M. (2007). Io e la mia malattia. In F. Bianchi di Castelbianco, M. Capurso, &

M. Di Renzo (Eds.), *Ti racconto il mio ospedale: esprimere e comprendere il vissuto della malattia* (pp. 19-85). Roma: Magi.

Capurso, M. (2017). *Facilitare la comprensione della malattia nel bambino: Aspetti teorici e indicazioni pratiche per medici, infermieri, psicologi e assistenti sociali*. Milano: Franco Angeli Edizioni.

Capurso, M. (2019). Communicating diagnoses and therapies to children. [Comunicare ai bambini diagnosi e terapie]. *Quaderni acp, 26*(4), 174-178.

Capurso, M., Bianchi di Castelbianco, F., & Di Renzo, M. (2020). "My life in the hospital". Narratives of children with a medical condition. *Continuity in Education, (in press)*. doi:10.5334/cie.12

Capurso, M., Lo Bianco, M., Cortis, E., & Rossetti, C. (2016). Constructing an Explanation of Illness with Children: A Sample Case Study of Juvenile Arthritis. *Child Care in Practice, 22*(3), 247-256. doi:10.1080/13575279.2015.1054788

Carey, S. (1987). *Conceptual Change in Childhood*: MIT Press.

Carugati, F., & Selleri, P. (2005). *Psicologia dell'educazione*. Bologna: Il Mulino.

Cole, M. (1998). *Cultural psychology: A once and future discipline*: Harvard University Press.

Crisp, J., Ungerer, J. A., & Goodnow, J. J. (1996). The Impact of Experience on Children's Understanding of Illness. *Journal of Pediatric Psychology, 21*(1), 57-72. doi:10.1093/jpepsy/21.1.57

Dempsey, A. G. (2019). *Pediatric Health Conditions in Schools: A Clinician's Guide for Working with Children, Families, and Educators*. New York: Oxford University Press.

Di Renzo, M., Marini, C., & di Castelbianco, F. B. (2013). *Il processo grafico nel bambino autistico*: Magi Edizioni.

Dixon-Krauss, L. (1998). Vygotskij nella classe. *Erickson, Trento*.

Donaldson, M. C. (1979). *Children's Minds*: Norton.

Eiser, C. (1985a). Children's Knowledge about Their Bodies and Illness *The Psychology of Childhood Illness* (pp. 18-40): Springer.

Eiser, C. (1985b). Effects of Chronic Illness on the Child, Family, and Siblings *The Psychology of Childhood Illness* (pp. 61-80): Springer.

Eiser, C. (1985c). *The psychology of childhood illness*: Springer-Verlag.

Eiser, C. (1989). Children's concepts of illness: Towards an alternative to the "stage" approach. *Psychology and health, 3*(2), 93-101.

Engel, G. L. (1977). The need for a new medical model: a challenge for biomedicine. *Science, 196*(4286), 129-136.

Ford, D. H., & Lerner, R. M. (1992). *Developmental systems theory: An integrative approach*: Sage Publications, Inc.

Freud, S. (1920/2013). *Al di là del principio di piacere* (2013 ed.): Bollati Boringhieri.

Friedman, H. S. (2013). *The Oxford Handbook of Health Psychology*: Oxford University Press.

Hart, R., & Rollins, J. (2011). *Therapeutic Activities for Children and Teens Coping with Health Issues*: Wiley.

Hauck, M. (1991). Cognitive abilities of preschool children: Implications for nurses working with young children. *Journal of Pediatric Nursing, 6*(4), 230-235.

Hergenrather, J. R., & Rabinowitz, M. (1991). Age-related differences in the organization of children's knowledge of illness. *Developmental Psychology, 27*(6), 952-959. doi:10.1037/0012-1649.27.6.952

Hill, M., & Tisdall, K. (2014). *Children and Society*: Taylor & Francis.

James, A., Jenks, C., & Prout, A. (1998). *Theorizing Childhood*: Teachers College Press.

Kalish, C. (1996). Causes and Symptoms in Preschoolers' Conceptions of Illness. *Child Development, 67*(4), 1647-1670. doi:10.2307/1131723

Koopman, H. M., Baars, R. M., Chaplin, J., & Zwinderman, K. H. (2004). Illness through the eyes of the child: the development of children's understanding of the causes of illness. *Patient Education and Counseling, 55*(3), 363-370. doi:http://dx.doi.org/10.1016/j.pec.2004.02.020

Kreitler, S., & Arush, M. W. B. (2004). *Psychosocial Aspects of Pediatric Oncology*: Wiley.

Leonhardt, C., Margraf-Stiksrud, J., Badners, L., Szerencsi, A., & Maier, R. F. (2014). Does the 'Teddy Bear Hospital' enhance preschool children's knowledge? A pilot study with a pre/post-case control design in Germany. *Journal of Health Psychology, 19*(10), 1250-1260. doi:10.1177/1359105313488975

Ligorio, B., & Cacciamani, S. (2013). *Psicologia dell'educazione*: Carocci.

Maslow, A. H. (1954/2010). *Motivazione e personalità*: Armando Editore.

McIntosh, C., & Stephens, C. (2012). A storybook method for exploring young children's views of illness causality in relation to the familial context. *Early Child Development and Care, 182*(1), 23-32. doi:10.1080/03004430.2010.534161

McIntosh, C., Stephens, C., & Lyons, A. (2013). Remember the bubbles hurt you when you cook in the pan: Young children's views of illness causality. *Psychology, Health and Medicine, 18*(1), 21-29. doi:10.1080/13548506.2012.687829

McQuaid, E. L., Howard, K., Kopel, S. J., Rosenblum, K., & Bibace, R. (2002). Developmental concepts of asthma: Reasoning about illness and strategies for prevention. *Journal of Applied Developmental Psychology, 23*(2), 179-194. doi:http://dx.doi.org/10.1016/S0193-3973(02)00103-X

Meadows, S. (2012). *The Child as Thinker: The Development and Acquisition of Cognition in Childhood*: Taylor & Francis.

Myant, K. A., & Williams, J. M. (2005). Children's concepts of health and illness: understanding of contagious illnesses, non-contagious illnesses and injuries. *Journal of Health Psychology, 10*(6), 805-819.

Nolte, T., Guiney, J., Fonagy, P., Mayes, L. C., & Luyten, P. (2011). Interpersonal stress regulation and the development of anxiety disorders: an attachment-based developmental framework. *Frontiers in Behavioral Neuroscience, 5*, 55-55. doi:10.3389/fnbeh.2011.00055

Perrin, E. C., & Gerrity, P. S. (1981). There's a Demon in Your Belly: Children's Understanding of Illness. *Pediatrics, 67*(6), 841-849. Retrieved from http://pediatrics.aappublications.org/content/67/6/841.abstract

Piaget, J. (1953). *The origin of intelligence in the child*. London,: Routledge & Paul.

Raman, L., & Winer, G. A. (2002). Children's and adults' understanding of illness: Evidence in support of a coexistence model. *Genetic, Social, and General Psychology Monographs, 128*(4), 325.

Redpath, C. C., & Rogers, C. S. (1984). Healthy Young Children's Concepts of Hospitals, Medical Personnel, Operations, and Illness. *Journal of Pediatric Psychology, 9*(1), 29-40. doi:10.1093/jpepsy/9.1.29

Rogers, C. R., Kinget, G. M., & Galli, R. (1970). *Psicoterapia e relazioni umane*: Bollati Boringhieri.

Rushforth, H. (1999). Practitioner Review: Communicating with Hospitalised Children: Review and Application of Research Pertaining to Children's Understanding of Health and Illness. *The Journal of Child Psychology and Psychiatry and Allied Disciplines, 40*(05), 683-691. doi:doi:null

Sarmento, M. J. (2006). Conoscere l'infanzia: I disegni dei bambini come produzioni simboliche. *Infanzia e società, 2*(1-2), 21.

Shaffer, D. R., & Kipp, K. (2013). *Developmental psychology: Childhood and adolescence*: Cengage Learning.

Sharp, K. C. (1982). Preschoolers' Understanding of Temporal and Causal Relations. *Merrill-Palmer Quarterly, 28*(3), 427-436. Retrieved from http://www.jstor.org/stable/23086126

Shorter, E. (2005). The history of the biopsychosocial approach in medicine: before and after Engel. In W. Peter (Ed.), *Biopsychosocial medicine: An integrated approach to understanding illness* (pp. 1-19). New York: Oxford University Press.

Siegal, M. (1988). Children's Knowledge of Contagion and Contamination as Causes of Illness. *Child Development, 59*(5), 1353-1359. doi:10.2307/1130497

Siegal, M., & Peterson, C. (2005). *Children's Understanding of Biology and Health*: Cambridge University Press.

Siegler, R. S. (1976). Three aspects of cognitive development. *Cognitive Psychology, 8*(4), 481-520. doi:http://dx.doi.org/10.1016/0010-0285(76)90016-5

Sperber, D., Premack, D., & Premack, A. J. (1995). *Causal Cognition: A Multidisciplinary Debate*: Clarendon Press.

Springer, K., & Ruckel, J. (1992). Early beliefs about the cause of illness: Evidence against immanent justice. *Cognitive Development, 7*(4), 429-442. doi:http://dx.doi.org/10.1016/0885-2014(92)80002-W

Varkula, L. C., Resler, R. M., Schulze, P. A., & McCue, K. (2009). Pre-school children's understanding of cancer: the impact of parental teaching and life experience. *Journal of Child Health Care*. doi:10.1177/1367493509347115

Vygotskij, L. S. (1934). *Pensiero e linguaggio* (1990 ed.): Giunti Editore.

Vygotskij, L. S. (1978). *Mind in society: the development of higher psychological processes* (M. Cole Ed.). Cambridge, Mass.; London: Harvard University Press.

Walsh, M. E., & Bibace, R. (1991). Children's Conceptions of AIDS: A Developmental Analysis. *Journal of Pediatric Psychology, 16*(3), 273-285. doi:10.1093/jpepsy/16.2.273

Wilkinson, S. R. (2006). *The Child's World of Illness: The Development of Health and Illness Behaviour*: Cambridge University Press.

Wood, D. (1998). *How Children Think and Learn*: Wiley.

Wood, D., Bruner, J. S., & Ross, G. (1976). The role of tutoring in problem solving*. *Journal of Child Psychology and Psychiatry, 17*(2), 89-100.

Yoos, H. L. (1993). Children's illness concepts: old and new paradigms. *Pediatric Nursing, 20*(2), 134-140, 145.

Zhu, L., Liu, G., & Tardif, T. (2009). Chinese children's explanations for illness. *International Journal of Behavioral Development, 33*(6), 516-519. doi:10.1177/0165025409343748

Capitolo 3

Aiutare bambini e ragazzi a rielaborare vissuti stressogeni a scuola

Giulia Cenci, Giulia Gizzi, Livia Buratta, Elisa Delvecchio, Claudia Mazzeschi

3.1 Bambini e ragazzi tra sfide quotidiane e stress

Come affermano Skinner e Zimmer-Gembeck (2016) nonostante l'infanzia e la preadolescenza siano spesso dipinte nei libri e nei film come una fase felice e spensierata della vita, durante questo periodo bambini e ragazzi affrontano numerose sfide e ostacoli. Alcuni di questi problemi hanno una natura temporanea e sono tipici del vivere quotidiano, come discutere tra fratelli sul possesso di un giocattolo, cadere per imparare ad andare in bicicletta, affrontare la delusione legata alla sconfitta della squadra del cuore, impegnarsi per prendere un bel voto ad una interrogazione a scuola; altre sfide invece derivano da problemi più complessi e si configurano come dei veri e propri potenziali *stressor* (o fattori di stress o anche, fattori stressanti) quali, ad esempio, affrontare la separazione dei genitori, la nascita di un fratellino o l'ingresso in una nuova scuola. Vi sono poi degli *stressor* cronici connessi a situazioni più serie, come gravi deprivazioni familiari, vivere in situazioni di guerra, affrontare gravi e prolungate malattie, personali e/o del contesto. Infine, a livello ancora più alto su una scala della gravità degli eventi stressanti, troviamo gli eventi traumatici veri e propri, quali gli abusi, i maltrattamenti, gli attentati terroristici e i disastri naturali come terremoti o alluvioni che colpiscono la propria abitazione. Durante il loro percorso di crescita, dunque, bambini e ragazzi possono trovarsi ad affrontare un'ampia gamma di eventi stressanti. In questa prospettiva anche l'improvvisa pandemia di Covid-19, causata dal SARS-CoV-2, rappresenta una di quelle situazioni che mettono alla prova la vita di chi sta crescendo. È per questo che Garmezy (1986) afferma: "i bambini non sono estranei agli eventi stressanti!". Eventi di questo tipo possono diventare traumatici se giungono a provocare una ferita o un danno al benessere emotivo o fisico della persona (Koplewicz & Cloitre, 2006).

Le risposte dei bambini agli eventi stressanti, paurosi e più tragicamente traumatici sono legate ad un'ampia gamma di fattori esterni e interni alla persona. In particolare, i modi in cui bambini e ragazzi sapranno comprendere e reagire all'evento stressante dipendono dal livello di esposizione all'evento, dall'età e dalla capacità di comprensione della situazione, dal genere, dal funzionamento psicologico della persona prima dell'evento, dallo stile di personalità, dai cambiamenti che l'evento ha imposto nelle situazioni di vita, dalla rete di supporto, da precedenti perdite o traumi. Inoltre, specialmente durante il verificarsi dell'evento, le reazioni dei bambini sono largamente influenzate dagli adulti prossimi i quali, a loro volta, devono far fronte alle loro personali reazioni. Bisogna inoltre tenere presente che in tutto questo bambini e ragazzi non sono soggetti passivi, ma, come vedremo, essi stessi possono contribuire attivamente a modificare se stessi e l'ambiente circostante per contribuire alla creazione di caratteristiche favorevoli al proprio sviluppo e adattamento (Bronfenbrenner, 2010).

Scopo di questo breve capitolo è analizzare i principali processi psicologici che consentono a bambini e ragazzi di affrontare gli eventi potenzialmente stressanti e di attivare le proprie risorse mentali, sociali e relazionali per farvi fronte in modo efficace. Tutto questo sarà di aiuto agli insegnanti che dovranno riaccogliere gli alunni in classe dopo l'epidemia di Covid-19. Le appendici di questo testo contengono poi una serie di schede operative utili a strutturare, in classe, alcune attività di supporto per gli alunni.

3.2 Alcune definizioni generali

3.2.1 Lo stress

Stress è oggi spesso usato come un termine ombrello e come tale esprime un'ampia gamma di concetti e costrutti dai significati piuttosto diversi (Argentieri e Gosio, 2015). In termini etimologici, la parola deriva dal latino *strictus*, stretto; ed è presente in diverse lingue (in inglese *stress*, sforzo; in francese *estrece*, strettezza, oppressione). Sovente, nell'uso corrente, il termine indica una "tensione, un logorio, un affaticamento" ma anche "…il fatto, le situazioni che ne costituiscono la causa" (Vocabolario Treccani). Una delle ambiguità della parola risiede proprio nel fatto che a volte con "stress" si indica lo stato interno di tensione dell'individuo, mentre in altre situazioni si fa riferimento alla situazione, al fatto o all'evento che ne costituisce la causa e che produce tale stato di tensione interna alla persona.

Una classica e interessante teoria dello stress e del modo di affrontarlo, definita teoria cognitivo-transazionale, viene proposta da Lazarus negli anni Ottanta (Lazarus, 1966; Lazarus & Folkman, 1984) come ampliamento di una precedente teorizzazione di Seyle (1976) (considerato il padre della ricerca sullo stress). Secondo questo modello, un evento è definito stressante non in base ad una sua gravità o difficoltà oggettiva, bensì in base alla percezione che il soggetto ne ha. Più precisamente, l'individuo valuta

cognitivamente l'evento/fatto esterno in due fasi: la valutazione *primaria,* in cui il soggetto giudica la situazione in base alle caratteristiche dell'evento; e la valutazione *secondaria* in cui l'individuo valuta le risorse personali e ambientali necessarie per affrontare la situazione. In altre parole la definizione e comprensione dello stress derivano dalla valutazione che la persona fa delle proprie capacità di interagire con l'ambiente; si valutano, cioè, le diverse possibilità di interazione persona-ambiente nel momento in cui ci si trova ad affrontare un dato compito o difficoltà. In tutto questo la persona sperimenta (o anticipa) che i suoi bisogni, obiettivi, valori o benessere sono (o saranno) sfidati, minacciati, o danneggiati (Lazarus & Folkman, 1984). Lo stress diventa quindi legato alla valutazione soggettiva delle capacità dell'organismo di rispondere a stimoli derivati da cambiamenti dell'ambiente, i cosiddetti "stressors" (Ammaniti, 2010). Facciamo un esempio. Un pilota di linea alle prime armi, che deve far decollare per la prima volta un Airbus A380 carico di 800 passeggeri, affronterà con molto stress questo compito. Ma se al comando dello stesso aereo sedesse un pilota esperto, che da 20 anni vola su quel medesimo aeroporto e con lo stesso tipo di aereo, possiamo ipotizzare che lo stress vissuto dal pilota sarebbe molto minore. Dunque il compito da affrontare è lo stesso, ma il livello di stress cambia perché nei due piloti cambia la percezione del rapporto compito/capacità del soggetto.

Lo stress, secondo il modello di Lazarus e Folkman (1984), va quindi considerato come una funzione essenziale per il nostro organismo, in quanto ci permette di far fronte alle richieste, ma anche alle pressioni e alle minacce esterne, ossia offre il vantaggio di adattarci all'ambiente circostante; tutto ciò è fondamentale per la sopravvivenza dell'essere umano.

Tuttavia, come ciascuno di noi avrà avuto modo di sperimentare più volte, lo stress non assume sempre una valenza positiva per la nostra persona. Lo stress che consente l'attivazione di funzioni adattive rispetto al problema, che ci permette per esempio di affrontarlo dedicando ad esso una giusta dose di attenzione ed energia, viene definito *eustress.* Quando la situazione stressante richiede uno sforzo di adattamento superiore alle possibilità percepite dall'individuo, si parla invece di *distress. Il distress* comporta un logorio progressivo che può portare al deterioramento delle difese psicofisiche della persona, anche e soprattutto nel soggetto in età evolutiva.

Alla luce di questa distinzione tra distress ed eustress, nel seguito di questo lavoro vedremo di dare delle risposte più articolate alle possibili domande e questioni che possono coinvolgere il lavoro dell'insegnante. Per quanto riguarda gli eventi collegati alla recente epidemia di coronavirus e ai vissuti di stress ad esso collegati, dobbiamo fare alcune considerazioni. La prima è che l'emergenza che abbiamo vissuto in questo periodo va considerata come uno stressor collettivo e dunque assume determinate caratteristiche che oltrepassano la dimensione individuale (Barton, 1970; Mawson, 2005), avendo coinvolto l'intera popolazione italiana e gran parte di quella mondiale. Si tratta di un vero e proprio evento di vita, una situazione nuova e diversa, un potenziale fatto-

re di discontinuità che ha modificato la continuità nella vita dei bambini nonché delle loro famiglie e delle comunità intere.

Per quanto riguarda bambini e ragazzi in età scolare, una prima domanda che appare necessario porsi è se quello che i bambini e i ragazzi percepiscono, conoscono e vivono o hanno percepito, conosciuto e vissuto sul coronavirus può essere definito come un evento che ha provocato stress (e quindi conseguente adattamento) o distress (e quindi con conseguenze di disadattamento). Si tratta di un quesito fondamentale perché, come abbiamo visto, la distinzione tra eustress e distress evidenzia le capacità *versus* incapacità di adattamento, di coping. Naturalmente la risposta a questo quesito non è universale ma sarà diversa da persona a persona. Inoltre, anche nei casi in cui l'emergenza legata agli eventi del coronavirus sia stata vissuta come distress, oltre a possibili interventi di professionisti del settore psicologico, esistono possibili spazi di intervento che possono coinvolgere anche la scuola e la vita di classe.

3.2.2 Lo stress in età evolutiva

La psicologia dello sviluppo, nei suoi diversi orientamenti, si è da tempo interessata ad indagare le molteplici condizioni stressanti vissute da bambini e adolescenti. In particolare, si indaga come lo stress possa incidere sullo sviluppo e sulla vita di bambini e adolescenti (Skinner & Zimmer-Gembeck, 2016). Infatti, l'esposizione acuta o cronica allo stress in differenti fasi della vita può produrre effetti diversi (Lazzari, 2019), mantenendosi allo stato di "eustress" o divenendo quello che abbiamo sopra definito come "distress".

Esiste una tradizione di studi sullo stress in età evolutiva e sulle relative risorse che bambini e ragazzi sono in grado di mobilitare per far fronte a quelle che vengono definite situazioni stressanti acute e/o croniche. In particolare è possibile identificare diversi livelli di analisi: un livello individuale, che si concentra sulle caratteristiche specifiche "interne" di un soggetto e una prospettiva multi-sistemica che include un'analisi delle caratteristiche dell'intero sistema in cui il bambino vive. A partire dagli anni Sessanta, gli studi sui fattori di resilienza ad eventi stressogeni hanno ampliato la prospettiva di studio oltre le caratteristiche individuali, ponendo attenzione anche ad altri aspetti, seguendo un approccio multi-livello e multi-sistemico volto a comprendere la famiglia, la scuola, il gruppo dei pari, i fattori connessi allo status socioeconomico e la stessa cultura (Masten, 2006; Werner, 1993).

3.2.3 La funzione dello stress in età evolutiva

In una cornice generale, durante l'infanzia, la funzione dello stress ha un andamento che segue una curva a campana piuttosto che lineare rispetto alle possibili conseguenze evolutive future. Ad una estremità della curva, la completa mancanza di fattori di stress anche lievi può non stimolare il bambino a sviluppare risorse utili in futuro; all'altra estremità, invece, fattori di distress più gravi possono sopraffare le capacità del bambino di rispondere e gestire la situazione (Liu, 2015). Relativamente alle situazioni inter-

medie, studi recenti hanno parlato di "effetto steeling" (Garmezy, 1986; Rutter, 2012), di "inoculazione da stress" (Gunnar, Frenn, Wewerka & Van Ryzin, 2009) e di "anti-fragility" (Taleb, 2012), cioè di come l'esposizione a fattori di eustress moderati nelle prime fasi della vita possa rendere la persona più resiliente verso potenziali esperienze negative e fattori di stress successivi. Si tratta di una sorta di "vaccinazione" allo stress, in cui eustress moderati rappresentano delle opportunità per lo sviluppo di risorse sociali e interne al bambino che gli consentiranno di fronteggiare in modo più efficace le difficoltà future. Questo dato si applica, probabilmente, anche alle risposte relative all'emergenza prodotta dalla recente epidemia causata dal nuovo SARS-Cov-2. Dobbiamo cioè chiederci se, e in quale misura, i soggetti che frequentano le nostre scuole sono cresciuti in un ambiente che li ha sufficientemente "vaccinati" contro un distress eccessivo, rendendoli capaci di affrontare questo nuovo evento di crisi. L'andamento a campana di cui parlavamo prima indica che la maggior parte dei soggetti si situa all'interno delle situazioni intermedie e quindi, per così dire, tra i "vaccinati". Quindi è probabile che i soggetti più fragili rispetto agli effetti negativi dello stress saranno presenti ma in minoranza. Sicuramente caratteristiche individuali di ogni soggetto possono influire su questa fragilità. Ad esempio studi recenti hanno comunque riportato l'attenzione su fattori genetici individuali (Luthar, 2006; Rutter, 2002).

Come si diceva poc'anzi, il livello di analisi individuale non è da solo sufficiente a spiegare le ragioni dello stress e le capacità di gestirlo. Secondo una prospettiva sistemica, l'ambiente di vita, da quello strettamente familiare fino a quello socio-culturale più ampio, influenza la funzione adattiva della persona allo stress come pure quelle disadattive generate dal distress.

Per comprendere queste dinamiche prendiamo in esame il fondamentale contributo di Bronfenbrenner (1986). L'autore ha concettualizzato un approccio multi-sistemico che organizza i diversi livelli dell'ambiente di sviluppo del bambino, come una serie di sotto-sistemi concentrici, in interazione tra loro, che influenzano direttamente e indirettamente lo sviluppo del bambino, e che possono anche essere classificati, secondo la terminologia di Bornstein, in fattori *prossimali* e *distali* (Bornstein, 2002). I sistemi di cui ci parla Bronfenbrenner sono organizzati a livelli concentrici e hanno una diversa vicinanza rispetto al soggetto "bambino", definendo per così dire un gradiente di influenza basato sulla maggiore o minore distanza. Seguendo Bronfenbrenner, il "mondo" in cui il bambino vive è organizzabile (Bronfenbrenner, 1986) in:

- Microsistema: il contesto con cui il bambino ha esperienza diretta come la famiglia, il gruppo dei pari, la scuola, la squadra sportiva, ecc. Questi sono, nei termini di Bornstein, i fattori prossimali, in quanto immediatamente vicini al bambino, attraverso le sue interazioni e le relazioni
- Mesosistema: l'insieme delle relazioni che si instaurano tra i vari microsistemi. Anche questo è un livello quasi prossimale, che passa dagli scambi relazionali del mondo di mezzo in cui il bambino è immerso

- Esosistema: tutti i contesti in cui il bambino non è direttamente coinvolto ma che influenzano le sue relazioni il suo sviluppo indirettamente (il lavoro dei genitori, vicinato). In questo sistema il bambino può non essere direttamente coinvolto (ad esempio, un bambino può non conoscere il lavoro dei genitori o non essere mai andato nel loro luogo di lavoro) ma può risentire in maniera diretta e/o indiretta della sua influenza (es: pensiamo, ad esempio, al licenziamento di un genitore)
- Macrosistema: la cultura, i valori sociali, ideologie, istituzioni politiche, mass media. Questo è un livello distale che non ha un effetto diretto sul bambino ma che ha effetti sulla sua crescita attraverso gli effetti, ad esempio, delle scelte politiche-amministrative (comprese quelle sanitarie) che il governo (a sua volta votato, nei sistemi democratici) mette in atto.

Tale prospettiva ci aiuta a comprendere come un evento che susciti eustress o distress, agendo a diversi livelli del sistema, possa influenzare vissuti, comportamenti e sviluppo del bambino, in modo diretto (per così dire da vicino, prossimale) e indiretto (ovvero da lontano, distale). Ci permette anche di identificare i diversi livelli di intervento che possono essere attuati per potenziare le risorse a disposizione del bambino e/o del suo sistema per fronteggiare le conseguenze di questo evento critico.

La crisi causata dal nuovo coronavirus ha investito tutti i livelli, dal microsistema (si pensi, ad esempio, a come è cambiata la vita in famiglia) al macrosistema (si pensi ai media); tuttavia gli elementi più distali toccano più direttamente gli adulti che circondano il bambino ed è attraverso di loro che le conoscenze, gli atteggiamenti, gli affetti vengono poi trasmessi ai figli, ai nipoti e… agli stessi insegnanti. Gli aspetti più distali del multisistema non toccano direttamente il bambino ma vengono indirettamente comunicati a lui tramite i sistemi più prossimali: la famiglia, la scuola, ecc. Bambini e ragazzi, inoltre, vivono anche l'influsso di altri mezzi di comunicazione quali la televisione e i social network.

Relativamente al coronavirus ci sono e ci sono state regole, decreti legge, norme di regolazione di vita, tutto un "rumore" di fondo che circonda il soggetto in età evolutiva. Quanto di questo livello di fondo è arrivato al bambino e con quanto eustress e/o distress da parte dei sistemi più prossimali? Il bambino ha bisogno in primo luogo di sentire che le regole e i limiti imposti sono introiettati dal suo ambiente prossimale. Più le persone che gli sono vicine sono sentite come "poco autorevoli", più il bambino si sente stressato e in distress. Autorevolezza – come insegna la letteratura sulla genitorialità – implica capacità di mettere delle regole che hanno significato, anche se non sempre cognitivamente comprensibili.

Considerando la grande estensione temporale e territoriale dell'epidemia di Covid-19, sappiamo quanto questo abbia causato problemi di interruzione delle normali routine di vita e di costruzione di senso in tutta la famiglia, fino ad arrivare anche a gravi perdite e lutti, e di conseguenza che tutto questo sia passato anche ai bambini e

ai ragazzi sotto forma di incertezza e difficoltà a pensare ad un futuro ricco di opportunità evolutive.

Un ulteriore fattore da tenere presente per analizzare le capacità di bambini e ragazzi di comprendere situazioni stressanti è dato dal loro livello di sviluppo cognitivo e affettivo.

I bambini più piccoli sono maggiormente abituati ad affidarsi agli adulti e ad altre figure autorevoli quando si tratta di prendersi cura dei loro bisogni primari. Essi processano la realtà affidandosi, in parte, alle emozioni trasmesse dagli adulti che si prendono cura di loro (Bowlby, 1973).

Ma i bambini più grandi e poi i preadolescenti hanno necessità di avere delle spiegazioni più chiare rispetto a quanto accade nel loro ambiente circostante. Come abbiamo visto nel capitolo 2, essi utilizzano sempre di più le proprie capacità di ragionamento per dare un senso alla realtà. Accolgono più criticamente quello che gli viene detto, e usano le loro capacità cognitive e affettive per fare osservazioni, confronti e per mettere alla prova quanto gli viene comunicato in famiglia; questo vale per genitori, nonni, zii, insegnanti ecc. Trovare parole semplici per spiegare fenomeni complessi e apparentemente distanti, come una pandemia e le diverse leggi e decreti che l'accompagnano non sempre è facile, ma un bambino ha il diritto di comprendere un po' alla volta anche questo, ed è compito di chi educa sostenerlo anche lungo questo percorso di crescita.

3.2.4 Il ruolo specifico della famiglia

Relativamente alla famiglia, uno dei compiti principali dell'adulto in un momento particolare di eustress e/o distress quale quello legato all'emergenza del SARS-CoV-2 è quello di aiutare il bambino – parafrasando un po' Winnicott e un po' Vigotsky – a vivere in una zona di "sfida sufficientemente affrontabile". I caregivers devono cioè permettere al bambino di confrontarsi con i problemi che incontra aiutandolo a scoprire le proprie risorse, modulando in modo dinamico e flessibile le sfide, sulla base delle capacità del bambino, che, è bene ricordare, sono in continuo e costante sviluppo (Skinner & Zimmer-Gembeck, 2016; Lansdown, UNICEF & Innocenti Research Centre, 2005).

L'approccio multi-sistemico relativo all'elemento "prossimale" rappresentato dalla famiglia ci permette di meglio definire e comprendere la molteplicità dei fattori che possono intervenire nelle risposte dei bambini all'eustress e al distress alla luce del fatto che

- come sostiene Winnicott (1965), "il bambino non esiste da solo", ma nasce, vive e cresce in un contesto di relazioni (e di significati).
- l'esito dell'impatto di un evento stressante dipende da una serie di fattori che compongono il quadro dinamico e complesso dei fattori di rischio (es. una epidemia)

e di protezione (es. il ruolo dell'ambiente, familiare e scolastico) a valere sullo sviluppo, in un'ottica di sistema.

- i bambini e ragazzi sono persone attive e in via di costante sviluppo, ragione per cui – più che in un organismo adulto – un evento stressante può impattare in maniera diversa sulle fasi evolutive, sulla strutturazione dei meccanismi e dei processi di risposta allo stress, e nel caso di un distress che diventi acuto, condizionandone anche le traiettorie evolutive.

3.3. Reazioni emotive dei bambini di fronte ad eventi stressanti

Per comprendere le reazioni dei bambini di fronte ad eventi critici è importante adottare una prospettiva evolutiva e dinamica: il bambino è un soggetto attivo, in continua trasformazione (Lingiardi & McWilliams, 2017; Codispoti & Bastianoni, 2002) e le sue manifestazioni di disagio di fronte a eventi di vita negativi devono essere lette alla luce del momento evolutivo in cui si verificano.

Per comprendere i diversi sistemi di risposte a tali eventi è necessario considerare l'età, il livello di sviluppo cognitivo e affettivo raggiunto, la presenza di fattori di rischio e di protezione (come la resilienza e il sostegno che i *caregiver* riescono a fornire), che agiscono sulla vulnerabilità all'evento. Ad esempio, alcune manifestazioni che possono sembrare atipiche in bambini in età scolare, come la paura di dormire da soli, possono invece essere del tutto adeguate in bambini più piccoli (Codispoti & Bastianoni, 2002). Bisogna poi tenere sempre presente che non tutti i bambini reagiscono allo stesso modo.

Da recenti colloqui con genitori di bambini svolti presso il nostro Laboratorio di Psicologia dell'Università degli Studi di Perugia relativi al modo di vivere l'emergenza generata dal coronavirus, è emerso come in alcuni casi si possa manifestare uno stato di indifferenza e disorientamento per il fatto che la routine della scuola (e della giornata) è stata interrotta e alterata; si vive un tempo "insolito" con le figure genitoriali, senza amici né attività pomeridiane. In altri casi, i bambini appaiono tristi e/o più nervosi, sentendo la mancanza degli amici e dello sport, nonostante le videochiamate; possono essere altresì presenti tentativi di controllo della situazione, con bambini che fanno molte domande e seguono scrupolosamente le regole, talvolta anche in modo eccessivamente rigoroso.

Queste manifestazioni spesso rappresentano le modalità di comportamento con cui bambini e ragazzi tentano di fronteggiare e adattarsi ad un cambiamento significativo nella loro realtà quotidiana che può configurarsi come una vera e propria crisi adattiva (Lingiardi, & McWilliams, 2017). Tali reazioni quindi possono essere molto variabili e comprendere, tra le altre, tristezza, nervosismo, pianto, agitazione, manifestare mal di pancia o disturbi del sonno e dell'appetito. Si tratta, nella maggioranza dei casi, di risposte che tendono a promuovere un adattamento del bambino di fronte ad un fattore

stressogeno e hanno, generalmente, una durata limitata nel tempo. Quando tuttavia le risposte allo stress appaiono prolungate nel tempo e vanno ad incidere sul normale sviluppo e funzionamento del bambino possono dare spazio a disagi più profondi, come ad esempio problemi di socializzazione col gruppo dei pari o difficoltà di apprendimento a scuola (Bremner & Vermetten, 2001).

In relazione allo stress, Greenberg (1999) identifica 4 domini di fattori di rischio per lo sviluppo di disagio psicologico nel bambino che sono strettamente interconnessi tra di loro:

- Caratteristiche interne al bambino (vulnerabilità biologica, temperamento, capacità cognitive)
- Qualità delle relazioni con le figure di attaccamento
- Stile di *parenting* e stile di socializzazione
- Contesto familiare (eventi critici, stress familiari, traumi, rete sociale).

3.4. Le risorse di bambini e ragazzi di fronte allo stress: resilienza, strategie di coping, supporto sociale e identità positiva

Bambini e adulti di fronte a eventi stressanti possono attivare diverse risorse per fronteggiare e riparare gli effetti negativi correlati allo stress, arrivando anche a trasformare un evento critico in un'opportunità di cambiamento (Di Nuovo, 2017). I ricercatori oggi concordano sul fatto che, in condizioni normali, l'infanzia rappresenta un periodo particolarmente "solido" (Compas et al., 2001; Skinner & Zimmer-Gembeck 2007; Skinner & Zimmer-Gembeck, p. 183), nel quale bambini e ragazzi possono resistere a situazioni di stress e talvolta anche maturare (Masten, 2001). In particolare, nella risposta allo stress, i processi di resilienza e le strategie di coping rivestono una fondamentale importanza.

La resilienza è definita da Masten e Reed (2002) come "l'insieme dei fenomeni che caratterizzano un adattamento soddisfacente all'ambiente, pur in condizioni di avversità o rischio elevato. Per essere definito resiliente, pertanto, un individuo deve ottenere buoni risultati nei vari ambiti della vita, pur trovandosi esposto a situazioni altamente problematiche". La resilienza permette all'individuo di allontanarsi dall'idea di trovarsi in un mondo già determinato e aprirsi alla possibilità che esista qualcosa di diverso cui è sempre possibile adattarsi (Malaguti & Cyrulnik, 2005).

I pioneristici lavori di Anna Freud avevano già evidenziato alla fine della seconda guerra mondiale come, nonostante gli effetti traumatizzanti della guerra, i bambini fossero in grado di ripristinare un buon livello di funzionamento psicologico (Freud & Burlingham, 1943). La resilienza può essere sviluppata, infatti, in età infantile nel caso in cui genitori ed educatori promuovano nel bambino fiducia, autonomia, sviluppo dell'identità personale, iniziativa e industriosità (Grotberg, 2003).

Come già anticipato, la ricerca ha evidenziato l'importanza della resilienza, ponendo attenzione ad alcuni fattori predisponenti come i fattori genetici, le risorse persona-

li, la storia individuale, il contesto familiare e le strategie di coping (Williams & Merten, 2009). Tra i fattori appresi, che influenzano la resilienza nei bambini – costituendo pertanto fattori da potenziare – Masten (2007) indica:

- un positivo concetto di sé
- capacità di autoregolazione
- competenze sociali
- flessibilità cognitiva e adattabilità a nuove situazioni
- problem solving
- capacità di comunicazione
- empatia
- assertività
- umorismo
- spiritualità
- capacità di attivare comportamenti di cura.

In particolare, un attaccamento sicuro, positive relazioni con i caregiver e con il gruppo dei pari forniscono una base relazionale ed emotiva sicura per il bambino e questo, a sua volta, facilita la tolleranza alla frustrazione, una maggior regolazione emotiva, comportamenti pro-sociali e una migliore gestione dello stress.

In questo contesto la scuola e gli insegnanti possono promuovere la resilienza nei bambini favorendo la socializzazione, offrendo la possibilità di sperimentare successo e coltivando negli alunni un senso di competenza nel fare le cose (Masten, 2007). La resilienza, infatti, è una capacità che si costruisce nel tempo, che può e deve essere promossa per fronteggiare situazioni di stress e pericolo, per attivare un cambiamento positivo ed efficace nella persona e ancor più in bambini e ragazzi. L'individuo si mostra resiliente nel momento in cui, in seguito ad un evento traumatico, riesce a rientrare in contatto con la sua rete sociale ed evitare così l'isolamento, sentendosi protetto e sostenuto dal suo gruppo di appartenenza.

L'intervento in situazioni di crisi deve quindi essere guidato dal concetto di resilienza (Malaguti & Cyrulnik, 2005; Goldstein & Brooks, 2005) e si suggerisce agli educatori di operare per la valorizzazione delle risorse presenti, piuttosto che concentrarsi unicamente sulle conseguenze dell'evento stressogeno.

Per sostenere i processi di resilienza, l'individuo risponde alle richieste dell'ambiente mettendo in atto una serie di strategie che gli consentono di superare lo stress e gestire e risolvere efficacemente i propri problemi quotidiani. Tale strategie vengono definite in letteratura come strategie di coping (letteralmente, *fronteggiamento;* Folgheraiter, 2003). Con questo termine si indica l'insieme delle azioni (che si attuano attraverso diverse strategie), sia cognitive che comportamentali, che il soggetto mette in atto per cambiare e/o gestire le richieste interne e/o esterne (gli stressor). Si tratta dunque di un processo attivo, una risposta appresa e *consapevole* che il soggetto usa per far fronte alle difficoltà e agli stressors (Folgheraiter, 2003).

L'adozione di strategie di coping adeguate al compito da affrontare permette al soggetto di gestire lo stress riducendo al minimo il rischio di sviluppare patologie, sia fisiche che psicologiche (Delle Fave & Bassi, 2013). Questo avviene perché tramite il coping si lavora sul senso di agency della persona (Bruner, 2009), cioè sulla sua capacità di vedere delle possibilità di azione e percepirsi capace di effettuarle. Lavorando sul coping, l'insegnante aiuta bambini e ragazzi a sostituire alla paura di soccombere davanti al problema il senso di poter fare, tramite un agire concreto. La scheda 2 riportata in appendice a questo testo lavora proprio in tale direzione.

Per quanto riguarda le strategie di coping, in un classico lavoro di classificazione, Folkman e Lazarus (1988) ne individuano otto tipologie: attivazione di confronto, distanziamento, autocontrollo, ricerca di supporto sociale, accettazione della responsabilità, fuga ed evitamento, problem solving programmato, rivalutazione positiva. Tali strategie sono poi state classificate dagli autori in due grandi categorie: da una parte, le forme di coping centrate sul problema (in cui il soggetto analizza il problema per comprenderlo meglio, lo elabora e esegue piani d'azione per la sua risoluzione); dall'altra, le forme di coping centrate sull'emozione (in cui il soggetto cerca di rimuovere il problema o di vederne solo il lato positivo, rivaluta positivamente l'evento e non ci pensa eccessivamente). Tale visione dicotomica è stata tuttavia molto criticata negli anni anche per il ruolo negativo assegnato alle emozioni il cui riconoscimento, comprensione, e condivisione può invece anche essere usato come forma di coping efficace (Stanton, Parsa, & Austenfeld, 2002). Nel 2003 esce un'altra opera fondamentale degli studi sul coping degli adulti. Si tratta di un ampio e dettagliato lavoro di revisione della letteratura svolto da Skinner, Edge, Altman, e Sherwood (2003). Analizzando i lavori del settore, gli autori identificano oltre 400 tipologie di coping diverse e sostengono quindi la necessità di mettere ordine nel campo, anche superando le visioni dicotomiche relative alle diverse forme di coping. Skinner et al. (2003) identificano quindi tredici "famiglie di coping", che servono tre grandi processi adattivi: coordinare le azioni con le contingenze del momento, coordinare la disponibilità delle risorse sociali, coordinare la scelta delle diverse azioni e strategie disponibili.

Per quanto riguarda i bambini e i ragazzi, l'età scolare può essere tuttavia considerata l'"età d'oro" del coping, momento in cui emergono nuove capacità e si consolidano quelle sviluppate durante la prima infanzia (Skinner & Zimmer-Gembeck, 2016, p. 182). Durante il periodo dello sviluppo, bambini e ragazzi apprendono gradualmente a valutare gli eventi stressanti, attribuendo a essi un significato che modellerà le loro reazioni emotive, cognitive e comportamentali (Losoya, Eisenberg, & Fabes, 1998; Spirito, 2003). I soggetti in via di sviluppo dispongono di una vasta gamma di meccanismi di coping con i quali fronteggiare in modo flessibile le diverse situazioni di stress, come ad esempio l'elaborazione delle proprie emozioni, la rivalutazione degli eventi e delle proprie possibilità, la capacità di auto-regolarsi. Non bisogna poi assolutamente dimenticarsi dell'importanza che il gioco, e la stessa possibilità di giocare, assumono

quando bambini e ragazzi si trovano a vivere situazioni stressogene. Come evidenziato da Capurso e Ragni (2016), infatti, il gioco svolge una grande serie di funzioni collegate alle strategie di coping: esso può funzionare come elemento di evasione da una realtà difficile o non sopportabile, oppure può esser usato come mezzo per provare strategie possibili. Giocare è anche un modo per incontrare gli altri, per mettersi alla prova, per stimolare immaginazione e creatività (Mazzeschi, Salcuni, Di Riso, Chessa, Delvecchio, Lis, Russ, 2016).

Tutte queste modalità di coping, se supportate e rafforzate da adulti presenti e sensibili, contribuiscono a definire il Sé del bambino. Infatti, l'auto-percezione di saper affrontare le difficoltà in modo efficace, che si basa sui modelli operativi interni sviluppati durante l'infanzia, è connessa a sentimenti di *mastery* (padronanza) e autoefficacia, di sicurezza e fiducia in sé e verso gli altri.

Tali dimensioni personali permettono al bambino di promuovere un'efficace autosufficienza, la ricerca di supporto in caso di difficoltà e lo sviluppo di un Sé integro ed efficace (Skinner & Zimmer-Gembeck, 2016). La scuola, il contesto sociale allargato e del gruppo dei pari possono operare per rafforzare le strategie di coping (Zani & Cicognani, 2000). In particolare, l'insegnante e la scuola rappresentano delle importanti fonti di sostegno per lo sviluppo di resilienza e strategie di coping, dato che sono in grado di fornire (House, 1981)

- sostegno emotivo (interesse, affetto, cura ed empatia verso l'altro)
- sostegno strumentale (intervento attivo sull'ambiente della persona)
- sostegno informativo (offrire suggerimenti, consigli e informazioni)
- sostegno affiliativo e di appartenenza (fornire stima, apprezzamento e stima per l'altro).

Tramite questo tipo di aiuto, bambini e ragazzi vengono messi nella condizione di rinforzare le abilità e le risorse di resilienza personale e diventano capaci di considerare un evento negativo come un'occasione per sviluppare nuove competenze. In questo modo si rafforza anche l'autoefficacia del soggetto (Bandura, 2004) che rappresenta la chiave per permettere a bambini e ragazzi di mettere in atto comportamenti preventivi e utilizzare strategie di coping adeguate alla situazione stressogena (Merluzzi & Sanchez, 1997).

3.5. Sentimenti di paura e ansia, altre manifestazioni di stress ed elementi traumatici.

La paura è un'emozione primaria fondamentale della nostra vita, che si sviluppa in risposta a situazioni di pericolo o percepite come potenzialmente dannose per l'essere umano (Epstein, 1972; Ohman, 1986; Rosen & Schulkin, 1998; Albano, Causey & Carter, 2001). Di base è un'emozione che svolge un'importante funzione adattiva, indispensabile per la sopravvivenza, diversa, ad esempio, dal terrore e panico. La paura è

spesso la prima risposta che il bambino esprime di fronte ad un evento nuovo e potenzialmente destabilizzante, se lo avverte come potenzialmente pericoloso.

Le paure in età infantili sono piuttosto comuni e variano per frequenza, intensità e durata lungo il periodo di sviluppo (Lis et al., 2013). I bambini si confrontano con la paura fin dai primi mesi di vita, sperimentando un'emozione fisiologica connessa agli adattivi processi di sviluppo, basti pensare alla paura dell'estraneo che il bambino manifesta intorno ai 4 mesi. Durante l'infanzia le paure si focalizzano soprattutto su stimoli di natura concreta presenti nel contesto di vita del bambino, come la paura per gli animali o per il buio. Successivamente, durante gli anni scolastici, emergono paure più astratte come quelle per i fenomeni soprannaturali (i fantasmi o i mostri), ma anche quelle per il fallimento e la critica, la paura per lesioni fisiche, la morte e l'abbandono da parte dei genitori (Gullone, 2000). Le paure connesse a temi più globali, come le preoccupazioni per condizioni economiche, politiche e sociali, sembrano maggiormente connesse alla fase adolescenziale (Angelino & Shedd, 1953). Nonostante non siano ancora completamente chiari i meccanismi che spiegano queste transizioni nelle paure del bambino, si ipotizza che tali modificazioni siano legate alla maturazione cognitiva del bambino, che gli permette di identificare e comprendere i reali pericoli in ogni situazione (Lis et al., 2013; King, Hamilton, & Ollendick, 1988; Ollendick, 1979). Possiamo quindi osservare come siamo predisposti, fin da piccoli, a confrontarci con le nostre paure e che la paura e le sue relative rappresentazioni sono degli aspetti normali e adattivi nello sviluppo durante tutto l'arco di vita.

Le paure inoltre rappresentano una prima forma di risposta protettiva verso quelle situazioni che il bambino non riesce pienamente a comprendere e controllare (Ollendick, Devis, & Muris, 2004). Pertanto, le paure devono essere differenziate da ansia e fobia, risposte più patologiche ad eventi negativi e stress, sulla base di alcune caratteristiche come l'intensità, la persistenza, la durata temporale, la pericolosità effettiva dell'oggetto temuto e l'interferenza con il normale funzionamento della persona (Miller, Barrett, & Hampe, 1974).

L'ansia è una uno stato di attivazione psicologica e organica, che nasce dal sentire un pericolo imminente ma vago e di origine sconosciuta. L'ansia quindi è una reazione emotiva di fronte a un evento, caratterizzata dall'attivazione di meccanismi fisiologici di allarme (Farnè, 2003). In età evolutiva è difficile distinguere tra ansia normale a ansia patologica, la differenza è da ricercare nella gravità, persistenza e incidenza dell'ansia sul funzionamento del bambino (Egger & Angold, 2006). Dunque non bisogna considerare solamente i sintomi ma anche, e soprattutto, come si collocano stress e sofferenza collegati all'esperienza soggettiva e interpersonale all'interno del funzionamento emotivo e adattivo del bambino (Costello, et al., 1993).

Elevati livelli di ansia sono normalmente presenti in soggetti esposti a situazioni stressanti (Lis et al., 2013). L'ansia nei bambini rappresenta un segnale affettivo di timore pervasivo e pericolo, conscio o inconscio, spesso associato a condizioni che minacciano la propria sicurezza come la malattia, la morte, conseguenze di un evento

improvviso (negativo ma anche positivo), la perdita di un *caregiver* o di un amico. L'ansia può esprimersi attraverso giochi ripetitivi circa il pericolo percepito o con un incremento dell'attività motoria che può servire a scaricarla, con sintomi somatici come disturbi gastrointestinali, dermatologici o dell'evacuazione; infine possono essere presenti disturbi del sonno, dell'alimentazione e comportamenti regressivi. In presenza di una sintomatologia d'ansia anche le relazioni sociali e le attività di socializzazione e di apprendimento possono esserne negativamente influenzate (Lingiardi & McWilliams, 2017).

La recente pandemia di Covid-19 ha comportato la perdita di migliaia di persone, ospedalizzazioni, quarantena. La comprensione completa dell'evento nelle sue caratteristiche epidemiche, mediche, socio-economiche e affettive è a carico degli adulti che con la loro maturità possono avere una visione generale della situazione. Bambini e ragazzi dispongono di diversi strumenti affettivi e cognitivi, di una rete relazionale più circoscritta, in gran parte basata sulla scuola e sulle attività pomeridiane che per lungo tempo sono state proibite. Tutto questo può aver provocato nel bambino vissuti di paura, ansia, stress e incertezza nonché di tristezza e depressione. Anche se l'emergenza dovuta al coronavirus è temporalmente delimitata e circoscritta, poiché essa rappresenta uno *stressor* primario, le conseguenze a breve e lungo termine dell'epidemia per i bambini possono essere più prolungate nel tempo e più difficilmente prevedibili. Infatti, la maggior limitazione che bambini e ragazzi hanno vissuto a causa della pandemia riguarda la restrizione di tutti quei luoghi e quelle attività che concorrevano al loro sviluppo: la chiusura delle scuole, dei luoghi di aggregazione e delle strutture sportive, l'isolamento con l'interruzione della normale routine del bambino e la perdita di occasioni di socializzazione formali (in classe, al catechismo, nello sport) e informali (giocare al parco con gli amici, le feste di compleanno, andare al cinema). Tutto questo può aver lasciato in bambini e ragazzi delle ferite psichiche che possono manifestarsi per mezzo di un'ampia serie di sintomatologie, anche nei mesi successivi alla fine dell'emergenza. Tra queste, possiamo elencare manifestazioni di tipo:

- Somatico, quali mal di stomaco e mal di testa. Sebbene siano disturbi tipici dell'infanzia (Jaycox, 2006), possono comparire in maniera più frequente e in specifiche situazioni rivelando così la possibile presenza di un fattore stressante. Il bambino può inoltre mostrarsi ipervigilante, apparire stanco a causa di notti poco riposanti e incorrere in variazioni repentine del peso corporeo (Brown, Brack & Mullis, 2008; NCTSN, 2006b; Openshaw, 2011)
- Comportamentale: i bambini più piccoli possono regredire e modificare il loro modo di giocare e comportarsi, mettendo in atto restrizioni e azioni ripetitive e riproducendo l'evento stressogeno (Lieberman, Chu, Van Horn, & Harris, 2011; NCTSN, 2006b; Terr, 1991). I più grandi invece possono riportare conseguenze più gravi a livello sociale: tendendo ad isolarsi, parlando troppo o troppo poco e adottando maggiori comportamenti a rischio. I bambini di tutte le fasce d'età, in

presenza di un fattore stressogeno, si comportano in modo da attirare l'attenzione dell'adulto e dimostrano un maggior livello di aggressività rispetto al loro standard (NCTSN, 2006b)
- Emotivo: difficoltà nella gestione e regolazione emotiva, con espressioni di rabbia, basso tono dell'umore, eccessiva paura, sentimenti di impotenza, scarsa fiducia in sé e negli altri (Jaycox, 2006; Milot, Ethier, St-Laurent, & Provost, 2010; Openshaw, 2011; Milot et al., 2010; NCTSN, 2006b; Terr, 1991)
- Cognitivo: manifestazioni lievi come difficoltà di concentrazione fino a espressioni più gravi come deficit nello sviluppo di alcune competenze (Jaycox, 2006; NCTSN, 2006b). Inoltre i bambini possono rivivere il trauma, sperimentare episodi di dissociazione e comportarsi in maniera differente nei confronti degli altri, della vita e del futuro (Openshaw, 2011; Terr, 1991).

Un discorso a parte merita quello che viene definito trauma psicologico con le sue conseguenze che vanno sotto la denominazione di PTDS (disturbo da stress post-traumatico). A differenza dello stress, del distress, della paura e dell'ansia e di altre manifestazioni finora esaminate, qui entriamo nell'ambito della psicopatologia e precisamente di quella che viene definita esperienza traumatica. Il trauma psicologico (dal gr. τϱαῦμα (-ατος) "ferita") rappresenta uno stress di gravità estrema, che minaccia la stessa integrità mentale. Si ha come conseguenza di un evento (o una sequenza di eventi) che deve produrre nell'individuo un'esperienza vissuta come "critica", eccedente cioè l'ambito delle esperienze normalmente da lui prevedibili e gestibili. L'aspetto fondamentale da tenere in considerazione è l'influenza che il pensiero o il ricordo dell'evento traumatico può avere sul funzionamento del bambino all'interno di contesti amicali, familiari, scolastici o di altro tipo. Per la natura stressogena di alcuni eventi e per il loro impatto sulla persona, le esperienze traumatiche possono distorcere le aspettative sul mondo, la sicurezza interpersonale e il senso di integrità personale di bambini e ragazzi. Queste aspettative, come ha osservato Bowlby (1973), contribuiscono a creare i modelli operativi interni del bambino, danno forma a concetti di sé e degli altri e conducono a previsioni rispetto al futuro che possono avere una profonda influenza sul comportamento attuale e futuro (Pynoos, Steinberg & Goenjian, 2006).

Il PTSD e le altre problematiche connesse ad eventi traumatici possono manifestarsi fin dal primo anno di vita e, quindi, ad una qualunque età (Breslau, Davis, & Peterson, 2000; Yule, Udwin, & Murdoch, 1990) ed esprimersi differentemente da bambino a bambino, manifestandosi anche anni dopo la fine dell'evento. Inoltre la sintomatologia post-traumatica sviluppata in età evolutiva può persistere anche a distanza di oltre 30 anni (Nicolais, Speranza, Bacigalupi & Gentile, 2005; Morgan, Scourfield, Williams, Jasper & Lewis, 2003). È stato addirittura evidenziato come situazioni di stress grave e ripetuto possano avere delle importanti ripercussioni sullo sviluppo cerebrale del bambino con significative ricadute in età adulta (ad es. Teicher, 2000; Perry, 2002). Per questo è importante saper osservare e riconoscere potenziali spie d'allarme, sin dal

loro esordio, cercando l'aiuto di un professionista se si sospetta che possano esservi segni di PTDS nei bambini o nei ragazzi con cui si lavora.

Le manifestazioni legate al PTDS possono essere rappresentate da sintomi intrusivi relativi all'evento come, ad esempio, il ricordo ricorrente, involontario e intrusivo; sogni spiacevoli senza un contenuto riconoscibile; flashback; sofferenza psicologica o reattività fisiologica. È possibile che il bambino riattualizzi l'esperienza dell'epidemia di Covid-19 e della quarantena tramite forme di ripetizione ossessiva veicolate dal gioco ripetitivo o da disegni ricorrenti. Inoltre bambini e ragazzi potrebbero non riuscire a evitare pensieri, sentimenti o sensazioni fisiche così come il ricordo di persone, luoghi o oggetti che riguardano l'evento, nonostante possano non ricordarne alcuni aspetti importanti. Quest'esperienza potrebbe generare in bambini e ragazzi pensieri negativi relativi alla propria persona, agli altri o al mondo accompagnati da sentimenti come tristezza, rabbia e spavento; chi cresce potrebbe inoltre non mostrarsi più interessato ad attività per lui fino ad allora stimolanti e tendere a isolarsi dal mondo esterno. Dal punto di vista comportamentale, potrebbe mostrarsi aggressivo, spericolato, ipervigilante, facilmente allarmabile, incapace di concentrarsi e avere maggiore difficoltà legate al sonno (DSM-5, APA, 2013).

3.6. Perché è importante offrire spazi per la rielaborazione socio-emotiva dei vissuti stressogeni

Poiché i soggetti in via di sviluppo non possono essere considerati al di fuori del loro contesto di vita diretto e indiretto, è importante lavorare su più piani del sistema di vita, tra cui quello scolastico, al fine di ripristinare le condizioni di vita che permettono un armonioso sviluppo e che possono essere state compromesse dal recente evento stressogeno (Codispoti & Bastianoni, 2002). È quindi molto importante il ruolo che l'insegnante e la scuola possono svolgere nel favorire il riadattamento di bambini e ragazzi al proprio ambiente di vita, sostenendoli nell'elaborazione dei propri vissuti.

Una delle prime ragioni per cui l'insegnante rappresenta una figura centrale nel processo di riadattamento del bambino nel periodo successivo all'epidemia di Covid-19 afferisce a responsabilità di natura etica e professionale. Se la scuola è il luogo nel quale si promuove uno sviluppo sano della persona, è necessario anche aprire in classe spazi di comunicazione e relazione che consentano in chi la frequenta anche la rielaborazione di vissuti potenzialmente stressogeni (Bell, Limberg, & Robinson, 2013).

Un secondo motivo è da ricercare nel ruolo che la figura dell'insegnante riveste nei confronti del bambino. L'insegnante, infatti, rappresenta per il bambino un *Altro significativo*, termine introdotto dallo psicoanalista Sullivan (1953) per indicare quelle persone che rappresentano dei punti di riferimento affettivi e dei modelli per l'apprendimento in diverse aree di funzionamento della persona. Attraverso la sua relazione con i bambini e i ragazzi, l'insegnante è in grado di influenzare l'immagine che questi hanno di sé e il loro stesso benessere (Belacchi & Gobbo, 2004). Infatti, la letteratu-

ra (Winnicott, 1965; Erikson, 1968) ha enfatizzato da tempo l'importante ruolo svolto dall'insegnante nella sua duplice funzione di soddisfazione e contenimento verso i bisogni cognitivi ed emotivi di chi cresce (Belacchi & Gobbo, 2004).

3.7. Cosa può fare la scuola?

La scuola rappresenta la situazione di normalità più comune, dove bambini e ragazzi trascorrono la maggior parte del proprio tempo. La scuola rappresenta anche un importante agente di socializzazione, dove chi cresce inizia a confrontarsi con gli altri e con il mondo esterno (Ziegler, 2014); è dunque naturale che essa possa svolgere un ruolo fondamentale per aiutare i soggetti in via di sviluppo a ritrovare un equilibrio e una serenità quotidiana, creando un ambiente sicuro, promuovendo relazioni sane, facilitando lo sviluppo di competenze emotive e capacità di coping (Bath, 2008). Attraverso il confronto con il gruppo dei pari e con gli insegnanti, l'ambiente scolastico rappresenta un prezioso spazio supportivo per il bambino, fondamentale anche per il recupero dei soggetti in situazioni difficili (Ellis, Nixon & Williamson, 2009).

Bambini e ragazzi possono sentirsi più a loro agio se ricevono aiuto da volti e ambienti familiari; pertanto, quando ci si deve confrontare con situazioni difficili, gli educatori possono svolgere un ruolo determinante nel sostenere chi cresce a confrontarsi con situazioni difficili, promuovendo così un sano sviluppo personale, sociale e accademico (Bell et al., 2013). Inoltre all'interno del contesto scolastico possono essere attuati interventi capaci di sostenere gli studenti a diversi livelli: individuale, attraverso un servizio di counseling psicologico; nel contesto di classe, attraverso interventi psicoeducativi come quelli che presentiamo in appendice, e a livello di istituto, attraverso un lavoro di rete con i servizi territoriali, le famiglie, le altre scuole e la comunità.

Nel periodo successivo al rientro a scuola dopo l'esperienza dell'isolamento causato dalla pandemia di Covid-19, gli insegnanti possono rappresentare degli interlocutori privilegiati per individuare situazioni di difficoltà e offrire supporto a bambini e ragazzi, attivando interventi specialistici che possono aiutarli a fronteggiare vissuti nuovi e difficilmente verbalizzabili. Una delle prime azioni che la scuola può svolgere per aiutare chi ha vissuto eventi stressogeni consiste nell'identificazione precoce di segnali di stress e trauma negli studenti e nella conseguente promozione di interventi di supporto a livello di istituto o in collaborazione con i servizi di competenza territoriale (Cook et al., 2017; Bell et al., 2013).

Per quanto concerne la possibilità di rielaborare esperienze traumatiche, Herman (1997) ha formulato un percorso che si sviluppa in tre momenti: la costruzione di un contesto stabile e sicuro, la fase di rievocazione e di espressione emotiva della situazione, e infine il riconnettersi con il proprio ambiente e con gli altri. Le attività che presentiamo in appendice 1 sono state elaborate proprio seguendo tale metodologia. A differenza di quanto avviene nei contesti terapeutici, dove il clinico propone, all'interno di un setting curativo, percorsi specifici di elaborazione dei vissuti emotivi più

profondi, l'insegnante può riprendere le sue attività di classe offrendo come prima cosa dei percorsi di condivisione socio-emotiva. Uno dei primi compiti per gli insegnanti al rientro in classe dopo l'isolamento vissuto da tutti a causa della Pandemia di Covid-19 sarà quello di ricostruire un clima di serenità e fiducia, in sé e negli altri. Si tratta anzitutto di aiutare bambini e ragazzi a riconnettersi con la comunità scolastica. È importante quindi riprendere la scuola ripartendo dagli aspetti relazionali, amicali, istituzionali che regolano la comunità. Tornare a sperimentare forme di cooperazione, ascolto, comprensione degli altri ed empatia; come pure, se necessario, la riscoperta di regole e limiti per il proprio comportamento. L'insegnante sarà di aiuto a chi rientra a scuola offrendo spazi di ascolto, mostrandosi attento e disponibile verso i bisogni degli studenti, dando informazioni che possano aiutarli a rielaborare l'evento accaduto senza cadere in ossessioni o fobie, strutturando in modo chiaro e accessibile l'insegnamento, fornendo strumenti per riprendere da dove si era lasciato e infine offrendo feedback positivi e in grado di rafforzare l'autostima di bambini e ragazzi. Tutto questo si può fare anche ripristinando in classe e nel contesto educativo le precedenti routine, rendendo la giornata quanto più prevedibile e "normale" per gli studenti, ma senza negare il passato o pretendere di fare come se non fosse successo niente.

È importante dunque lavorare su questi aspetti in un'ottica di prevenzione primaria e secondaria cercando di anticipare (se assenti) o gestire (se già presenti) eventuali sintomi di disagio psicologico.

Anche se molti bambini potrebbero mostrare segni di stress, ansia, o tristezza nelle prime settimane dopo la fine di un periodo complesso, la maggior parte di loro tornerà al proprio tipico stato di funzionamento fisico e emotivo. Tuttavia potrebbe non essere strano osservare reazioni atipiche e inusuali in alcuni di loro con cambiamenti che avvengono molte volte nei giorni o nelle settimane successive alla crisi. Mentre alcune di queste reazioni sono brevi e si risolvono da sé, altre possono durare più a lungo, per mesi o anche anni dopo l'evento. Per quei bambini che hanno più difficoltà a tornare alla normalità della vita, è bene rivolgersi – qualora necessario – a figure specializzate, che possano indirizzare insegnanti e genitori verso una maggiore comprensione della situazione, fornendo mezzi e strumenti utili al raggiungimento del benessere di chi cresce. In generale, nel segnalare un bambino ad un professionaista della salute mentale, i sintomi del disagio debbono essere valutati considerando la loro intesità, la durata, l'appropriatezza del comportamento rispetto all'età nonchè il livello di interferenza che comportano nella vita quotidiana: a scuola, a casa, nei rapporti con i compagni. Una valutazione professionale di una situazione identificata come atipica sarà un importante quanto necessario passo per un eventuale intervento di supporto o terapeurtico successivo.

Particolare attenzione dovrà inoltre essere posta agli alunni con bisogni speciali, alle persone con una disabilità, agli studenti con patologie mediche o psicologiche, a quanti vivono in condizioni di difficoltà o che stanno affrontando importanti momenti di transizione come separazioni, divorzi o lutti. In questi casi si raccomanda un maggior

supporto, anche rivolgendosi a specialisti, per assicurare la loro protezione e per sostenere al meglio chi deve affrontare un evento potenzialmente traumatico (Flynn, 2006).

Infine, dopo una lunga e forzata assenza da scuola, bambini e ragazzi avranno la necessità di tornare a sperimentare il buon funzionamento delle diverse componenti della loro identità. È dunque utile riprendere la scuola con attività che facciano riscoprire sentimenti positivi di gioia, piacere, divertimento, che sostengano un orientamento verso il futuro, che lavorino su immaginazione e creatività, che favoriscano una forma di riconoscimento positivo del sé da parte degli altri compagni e degli insegnanti.

Ringraziamenti

Le autrici del capitolo desiderano ringraziare la professoressa Adriana Lis, già professore ordinario di Psicologia Clinico-Dinamica, membro della Society for Personality Assessment (USA), dell'Association for Child Psychoanalysis (Usa) e dell'Associazione Italiana di Psicologia Scientifica (Italian Society for Scientific Psychology), nonchè autrice di numerosissime pubblicazioni nazionali e internazionali, per il prezioso contributo fornito alle riflessioni sulle dinamiche cliniche ed evolutive di bambini e ragazzi che vivono situazioni stressanti.

Bibliografia del capitolo

Albano, A.M, Causey, D., & Carter, B. (2001). Fear and anxiety in children. In C. E. Walker & M. C. Roberts (Eds.), *Handbook of clinical child psychology* (3rd ed., pp. 291-316). New York: Wiley.

American Psychiatric Association (APA) (2013), DSM-5. Manuale diagnostico e statistico dei disturbi mentali, tr. it. Raffaello Cortina, Milano, 2014.

Ammaniti, M. (2010) (a cura di). Psicopatologia dello sviluppo. Modelli teorici e percorsi a rischio. Raffaello Cortina, Milano.

Angelino, H., & Shedd, C. L. (1953). Shifts in the content of fears and worries relative to chronological age. Proceedings of the Oklahoma Academy of Science, 34, 180–186.

Argentieri, S., & Gosio, N. (2015). Stress e altri equivoci. Giulio Einaudi Editore.

Bandura, A. (2004). Health promotion by social cognitive means. Health education & behavior, 31(2), 143-164.

Barton, A. H. (1970). *Communities in disaster: a sociological analysis of collective stress situations*: Doubleday.

Bath, H. (2008). The three pillars of trauma-informed care. Reclaiming children and youth, 17(3), 17-21.

Belacchi, C., & Gobbo, C. (2004). Parlare con i bambini. L'interazione comunicativa nello sviluppo normale e patologico. Carocci.

Bell, H., Limberg, D., & Robinson III, E. M. (2013). Recognizing trauma in the classroom: A practical guide for educators. Childhood Education, 89(3), 139-145.

Bornstein, M. H. (ed.), Handbook of Parenting, vol. 5, Erlbaum, London 2002.

Bowlby J. (1973) Attachment and loss, vol. II: Separation: Anxiety and anger. London, Hogarth Press [trad. it. Attaccamento e perdita, vol. 2: La separazione dalla madre, Torino, Boringhieri, 1975].

Bremner, J. D., & Vermetten, E. (2001). Stress and development: Behavioral and biological consequences. *Development and Psychopathology, 13*(3), 473-489.

Breslasu N, Davis GC, Peterson EL (2000), A second look at comorbidity in victims of trauma: the posttraumatic stress disorder – major depression connection. Biological Psy- chiatry, 48, 902-909.

Breslasu, N., Davis, G.C., Peterson, E.L. (2000). "A second look at comorbidity in victims of trauma: the posttraumatic stress disorder – major depression connection". In Biological Psychiatry, 48, pp. 902-909.

Bronfenbrenner, U. (2010). *Rendere umani gli esseri umani: bioecologia dello sviluppo*. Trento: Erickson.

Bronfenbrenner, U. (1986). Ecology of the family as a context for human development: Research perspectives. Developmental psychology, 22(6), 723.

Brown, S.D., Brack, G., Mullis, F.Y. (2008). Traumatic symptoms in sexually abused children: Implications for school counselors. Professional School Counseling, 11(6), 368-379.

Bruner, J. S. (2009). *La mente a più dimensioni* (2 ed.). Roma; Bari: GLF editori Laterza.

Capurso, M., & Ragni, B. (2016). Bridge Over Troubled Water: Perspective Connections between Coping and Play in Children. *Frontiers in Psychology, 7*(1953). doi:10.3389/fpsyg.2016.01953

Codispoti, O., & Bastianoni, P. (2002). La diagnosi psicologica in età evolutiva. Carocci.

Compas, B. E., Connor-Smith, J. K., Saltzman, H., Thomsen, A. H., & Wadsworth, M. E. (2001). Coping with stress during childhood and adolescence: Problems, progress, and potential in theory and research. Psychological Bulletin, 127, 87–127. doi:10.1037/0033-2909.127.1.87

Cook, A., Spinazzola, J., Ford, J., Lanktree, C., Blaustein, M., Cloitre, M., DeRosa, R., Hubbard, R., Kagan, R., Liautaud, J., Mallah, K., Olafson, E., & van der Kolk, B. (2005). Complex Trauma in Children and Adolescents. *Psychiatric Annals, 35*(5), 390–398.

Costello, E.J., Burns, B.J., Angold, A.C., Leaf, P.J. (1993). "How can epidemiology improve mental health services for children and adolescents". In Journal of the American Academy of Child and Adolescent Psychiatry, 32, pp. 1106-1117.

Delle Fave, A., Bassi, M. (2013). Psicologia e salute. Esperienze e risorse dei protagonisti della cura. UTET Università, Torino.

Di Nuovo, S. (2017). Trauma e resilienza tra neuroscienze e aspetti psico-sociali. Journal of Applied Ceremonial and Communication in Management, 1, 24-43.

Egger, H.L., Angold, A. (2006). "Anxiety Disorders". In LUBY, J.L. (a cura di). Handbook of Preschool Mental Health. Development, Disorders, and Treatment. The Guilford Press, New York-London, pp. 137-164.

Ellis, A. A., Nixon, R. V., & Williamson, P. (2009). The effects of social support and negative appraisals on acute stress symptoms and depression in children and adolescents. British Journal of Clinical Psychology, 48(4), 347-361. doi:10.1348/014466508X401894

Epstein, S. (1972). The nature of anxiety with emphasis upon its relationship to expectancy. In C. D. Spielberger (Ed.), Anxiety: Current trends in theory and research (Vol.2, pp. 292-337). New York: Academic Press.

Erikson, E. (1968). Identity, youth and crisis. New York: Norton.

Farnè, M. (2008). L'ansia. Il Mulino.

Flynn BW (2006). Meeting the Needs of Special Populations in Disasters and Emergencies: Making It Work in Rural Areas. Presented at: National Association for Rural Mental Health, San Antonio TX, August 22, 2006.

Folgheraiter, F. (2003). Voce del Dizionario: Fronteggiamento (coping). *Lavoro Sociale, 3*(1), 127- 151.

Folkman, S., Lazarus, R.S., (1988). Ways of coping questionnaire. Permission set. Manual, test bookelet, scoring key, Palo Alto, CA, Mind Garden.

Freud A. & Burlingham D. (1943). War and Children, New York, Foster Parents' Plan for War Children.

Garmezy, N. Developmental aspects of children's responses to the stress of separation and loss. In: Rutter, M.; Izard, CE.; Read, PB., editors. Depression in young people: Developmental and clinical perspectives. New York: Guilford Press; 1986. p. 297-323.

Goldstein, S., & Brooks, R. B. (2005). Resilience in children. New York: Springer. Gooding, HC, Milliren, CE, Austin, SB, Sheridan, MA, & McLaughlin, KA (2016). Child abuse, resting blood pressure, and blood pressure reactivity to psychological stress. Journal of Pediatric Psychology, 41, 5-12.

Greenberg, M. (1999). L'attaccamento e la psicopatologia nell'infanzia, in J. Cassidy, P.R. Shaver (a cura di). Manuale dell'attaccamento: teoria, ricerca e applicazioni cliniche, trad. it. Fioriti, Roma 2002, pp. 535-65.

Grotberg, E. H. (Ed.). (2003). Resilience for today: Gaining strength from adversity. Greenwood Publishing Group.

Gullone, E. (2000). The development of normal fear: A century of research. Clinical psychology review, 20(4), 429-451.

Gunnar MR, Frenn K, Wewerka SS, Van Ryzin MJ. Moderate versus severe early life stress: Associations with stress reactivity and regulation in 10–12-year-old children. Psychoneuroendocrinology. 2009; 34:62–75. [PubMed: 18835102]

Herman, J. L. (1997). Trauma and recovery. New York, NY: Basic Books.

House, J. S. (1981). Work stress and social support. Reading, MA: Addison-Wesley.

Koplewicz, H., Cloitre, M. (2006). Caring for kids after trauma, disaster and death: a guide for parents and professionals. NYU Child Center * www.AboutOurKids.org

Jaycox, L.H. (2006). How schools can help students recover from traumatic experiences: A tool-kit for supporting long-term recovery. RAND.

King, N.J., Hamilton, D.I., Ollendick, T.H. (1988), Children's Phobias: A Behavioural Perspective. John Wiley & Sons, Chichester, England.

Lansdown, G., UNICEF, & Innocenti Research Centre. (2005). *The Evolving Capacities of the Child*. Firenze, Italy: United Nations Children's Fund.

Lazarus, R.S. (1966). Psychological Stress and the Coping Process. McGraw-Hill, New York

Lazarus, R.S., Folkman, C. (1984). Stress, Appraisal and Coping. Springer-Verlag, New York.

Lazzari, D. (2019). La psiche tra salute e malattia. Evidenze ed epidemiologia. Ed. Edra

Lieberman, A.F., Chu, A., Van Horn, P., Harris, W.W. (2011). Trauma in early child-

hood: Empirical evidence and clinical implications. Development and Psychopathology, 23(2), 397-410. doi:10.1017/S0954579411000137

Lingiardi, V., & McWilliams, N. (Eds.). (2017). Psychodynamic diagnostic manual: PDM-2. Guilford Publications.

Lis A., Di Riso D., Mazzeschi C., Chessa D. (2013). La valutazione dell'adattamento psicologico del bambino - I self – report. Raffaello Cortina, Milano.

Liu, R. T. (2015). A developmentally informed perspective on the relation between stress and psychopathology: When the problem with stress is that there is not enough. Journal of Abnormal Psychology, 124, 80–92. doi:10.1037/abn0000041

Losoya, S., Eisenberg, N., & Fabes, R. A. (1998). Developmental Issues in the Study of Coping. *International Journal of Behavioral Development, 22*(2), 287-313. doi:10.1080/016502598384388

Luthar, S. S. (2006). Resilience in development: A synthesis of research across five decades. In D. Cicchetti & D. J. Cohen (Eds.), Developmental psychopathology: Risk, disorder, and adaptation (2 ed., Vol. 3, pp. 739–795). New York: Wiley. doi:10.1002/9780470939406.ch20

Malaguti, E., & Cyrulnik, B. (Eds.). (2005). Costruire la resilienza: la riorganizzazione positiva della vita e la creazione di legami significativi. Centro Studi Erickson.

Masten, A. S. (2001). Ordinary magic: Resilience processes in development. American Psychologist, 56, 227–238. doi:10.1037//0003-066X.56.3.227

Masten, A. S. (2006). Developmental psychopathology: Pathways to the future. International Journal of Behavioral Development, 30, 47–54. doi:10.1177/0165025406059974

Masten, A. S. (2007). Resilience in developing systems: Progress and promise as the fourth wave rises. Development and psychopathology, 19(3), 921-930.

Masten, A.S., Reed, M.G.J. (2002). Resilience in development, in Handbook of Positive Psychology, a cura di C.R. Snyder e S.J. Lopez, Oxford University Press, New York, pp. 74-87.

Mazzeschi, C., Salcuni, S., Di Riso, D., Chessa, D., Delvecchio, E., Lis, A., Russ, S. (2016). *E tu giochi? La valutazione del gioco simbolico in età evolutiva: l'Affect in Play Scale.* Casa Editrice: Franco Angeli

Mawson, A. R. (2005). Understanding mass panic and other collective responses to threat and disaster. *Psychiatry: Interpersonal and biological processes, 68*(2), 95-113.

Merluzzi, T.V., Sanchez, M.A. (1997). Assessment of self-efficacy and coping with cancer: Development and validation of the cancer behavior inventory. "Health Psychology", 16, pp. 163-170.

Miller, L.C., Barrett, C.L., Hampe, E. (1974), Phobias of childhood in a prescientific era. In Davids, A. (a cura di), Child Personality and Psychopathology: Current Topics. Wiley, New York, pp. 89-134.

Milot T, Ethier LS, St-Laurent D, Provost MA (2010). The role of trauma symptoms

in the development of behavioral problems in maltreated preschoolers. Child Abuse Negl. 34(4):225-34. Epub 2010 Mar 29.

Morgan L., Scourfield J., Williams D, Jasper A, Lewis G (2003),The Aberfan disaster: 33-year follow-up of survivors. British Journal of Psychiatry, 182, 532-536.

National Child Traumatic Stress Network. (2006b). The effects of trauma on schools and learning. Retrieved December 15, 2011, from www.nctsnet.org/nccts/nav.do?pid=ctr_aud_schl_effects.

Nicolais, G., Speranza, A. M., Bacigalupi, M., & Gentile, L. (2005). Il trauma in età evolutiva: inquadramento diagnostico e correlate neurobiologici. Infanzia e adolescenza, 4, 187-199.

Ohman, A. (1986). Face the beast and fear the face: Animal and social fear as prototypes for evolutionary analyses of emotion. Psychophysiology, 23, 123-145.

Ollendick, T.H. (1979), Fear reduction techniques with children. In Hersen, M., Eisler, R.M., Miller, P.M. (a cura di), Progress in Behavior Modification, vol. 8. Academic Press, New York, pp. 127-168.

Ollendick, T.H., Davis, T.E. Iii, Muris, p. (2004), Treatment of specific phobia in children and adolescents" In Barrett, P., Ollendick, T.H. (a cura di), The Handbook of Interventions That Work with Children and Adolescents: From Prevention to Treatment. Wiley & Sons, West Sussex, UK.

Openshaw, L. (2011). School-based support groups for traumatized students. School Psychology International, 32(2), 163-178.

Perry, B.D. (2002). "Neurodevelopmental Impact of Violence in Childhood." In D.H. Schetky and E.P. Benedek (Eds.), Principles and Practice of Child and Adolescent Forensic Psychiatry (pp. 191–203; 200). Washington, DC: American Psychiatric Publishing, Inc.

Pynoos, R. S., Steinberg, A. M., Schreiber, M. D., & Brymer, M. J. (2006). Children and Families: A New Framework for Preparedness and Response to Danger, Terrorism, and Trauma.

Rosen, J. B., & Schulkin, J. (1998). From normal fear to pathological anxiety. Psychological review, 105(2), 325.

Rutter M. Resilience as a dynamic concept. Development and Psychopathology. 2012; 24:335–344. [PubMed: 22559117]

Rutter, M. (2002). The interplay of nature, nurture, and developmental influences: The challenge ahead for mental health. Archives of General Psychiatry, 59, 996–1000. doi:10.1001/archpsyc. 59.11.996

Seyle, H. (1976). Stress in health and disease. Boston, London: Butterworths

Skinner, E. A., Edge, K., Altman, J., & Sherwood, H. (2003). Searching for the structure of coping: A review and critique of category systems for classifying ways of coping. *Psychological bulletin, 129*(2), 216.

Skinner, E. A., & Zimmer-Gembeck, M. J. (2007). The development of coping. Annu. Rev. Psychol., 58, 119-144.

Skinner, E. A., & Zimmer-Gembeck, M. J. (2016). The development of coping from birth to emerging adulthood: Neurophysiological and social underpinnings, qualitative shifts, and differential pathways towards psychopathology and resilience.

Spirito, A. (2003). Child Coping. In S. Naar-King, D. A. Ellis, & M. A. Frey (Eds.), *Assessing children's well-being: A handbook of measures*. London: Lawrence Erlbaum Associates.

Stanton, A. L., Parsa, A., & Austenfeld, J. L. (2002). The adaptive potential of coping through emotional approach *Handbook of positive psychology* (pp. 148-158). New York: Oxford University Press.

Sullivan, H. S. (1953). The interpersonal theory of psychiatry. New York: Norton.

Taleb, NN. Antifragile: Things that gain from disorder. New York: Random House; 2012.

Teicher MH (2000), Wounds that time won't heal: The neurobiology of child abuse. Cerebrum, 2, 50-67.

Terr, L.C. (1983). "Chowchilla revisited: the effetcs of psychic trauma four years after a school-bus kidnapping." In American Journal of Psychiatry, 140, pp. 1543-1550.

Terr, L.C. (1991). Childhood traumas: An outline and overview. The American Journal of Psychiatry, 148(1), 10-20.

Werner, E. E. (1993). Risk, resilience, and recovery: Perspectives from the Kauai longitudinal study. Development and Psychopathology, 5, 503–515.

Williams, A. L., & Merten, M. J. (2009). Adolescents' online social networking following the death of a peer. Journal of Adolescent Research, 24(1), 67-90.

Winnicott, D. W., (1965) Sviluppo affettivo e ambiente: studi sulla teoria dello sviluppo affettivo. A. Armando, Roma 1974.

Yule W, Udwin O, Murdoch K (1990), The Jupiter sinking: effects on children's fears, depression and anxiety. Journal of Child Psychology and Psychiatry, 31, 1051-1061.

Zani, B., & Cicognani, E. (2000). Psicologia della salute. Il Mulino.

Ziegler, D. (2013). Optimum learning environments for traumatized children: How abused children learn best in school. Jasper Mountain.

Esercitazione 1

La lettura del libro è terminata. Per completare il corso di formazione riconosciuto dal Miur tramite la piattaforma SOFIA, è necessario:

1. Svolgere in classe le attività presentate nelle schede 1-6 dell'appendice 1 (le schede di lavoro per la classe sono scaricabili presso il link https://www.ebookscuola.com/librettorientrocovid)

2. Scaricare il modulo della Esercitazione 1 dalla propria area personale del portale ebookscuola.com e seguire le indicazioni per il suo svolgimento.

3. **Qualora nella propria zona le lezioni in classe non riprendessero nel corrente anno scolastico:** Scaricare ugualmente l'esercitazione 1 e compilare l'autocertificazione presente nell'ultima pagina, impegnandosi a svolgere le schede dell'appendice 1 alla ripresa dell'attività didattica.

Appendice 1

Attività per aiutare gli alunni a rielaborare i vissuti emotivi connessi all'esperienza del Coronavirus

Michele Capurso

Importante: Il gruppo di ricerca di Psicologia del dipartimento FISSUF dell'Università degli studi di Perugia è interessato a svolgere un'indagine basata sui contenuti prodotti da bambini e ragazzi nella loro compilazione delle schede di questo allegato. Gli insegnanti interessati a collaborare sono invitati a prendere visione della pagina https://www.ebookscuola.com/blog/ricercacovid per ricevere le istruzioni per partecipare all'iniziativa.

È necessario prendere contatti con il gruppo di ricerca prima di svolgere le attività in classe.

Le schede che presentiamo in questa sezione servono a svolgere con il gruppo classe un breve percorso di condivisione e rielaborazione dei vissuti emotivi al momento di rientro a scuola dopo il lungo periodo di isolamento causato dall'epidemia di Covid-19.

In tabella 1 vengono riassunti gli obiettivi e le funzioni principali di ogni scheda, assieme alle sezioni del libro forniscono le basi psicologiche che sottostanno ad ogni specifica attività presentata. Per quanto riguarda le attività proposte in questo allegato 1, esse vanno svolte esattamente nell'ordine proposto e secondo le indicazioni fornite, dato che nel loro insieme costituiscono un percorso logicamente strutturato. Spetta invece all'insegnante il compito di modulare le attività in base alle risposte dei propri alunni. Ad esempio, se alcune classi dimostreranno di avere bisogno di raccontare le proprie esperienze e le proprie emozioni, sarà utile assicurare un giusto periodo di tempo per farlo. È utile ricordare che le attività proposte hanno valore propedeutico e servono a garantire quel contenimento emotivo e a ristabilire quel clima di classe

necessario al successivo impegno cognitivo degli alunni. Sarebbe un errore gettarsi a capofitto nelle attività disciplinari di studio e valutazione senza aprire questo altro cruciale spazio comunicativo.

Una copia stampabile in A4 delle schede da svolgere in classe è **scaricabile presso** https://www.ebookscuola.com/librettorientrocovid

Tabella 1. *Sinossi delle attività proposte nell'allegato 1*

Finalità	Nr. Attività, Descrizione	riferimenti teorici per il docente	Dimensione su cui si interviene
Ricostruire un momento saliente del tempo trascorso in famiglia e condividerlo con i compagni.	**1.**Disegnaunmomentodiquestegiornate. Si chiede agli alunni di disegnare un momento che gli è rimasto particolarmente impresso delle giornate trascorse in famiglia a causa dell'emergenza del coronavirus.	**Cap. 3**	Sociale, familiare
Fornire occasioni di rielaborazione emotiva e di riflessione sulle proprie strategie di coping.	**2.** I Pensieri di Marco e Maria. Marco e Maria sono preoccupati ed hanno molti pensieri, che gli alunni scrivono sui fumetti e condividono tra loro.<br>**3.** Quando sono preoccupato. Si chiede ai bambini di descrivere alcune preoccupazioni comuni, collegandole alle proprie strategie di coping e alle persone di riferimento.	**Cap. 3**	Emotiva<br><br>Emotiva e cognitiva
Creare un riscontro concreto alla propria immaginazione e usare il pensiero logico per ricostruire le strategie utili a proteggersi.	**4.** Disegna il Coronavirus. Si chiede ora ai bambini di immaginare il Coronavirus e disegnarlo. Questa scheda serve a capire quale tipo di immagine hanno i bambini della malattia e a creare un riscontro concreto del virus che la causa. Tale scheda prepara dunque il passaggio ad un lavoro cognitivo che si svolge poi con la scheda 5 e con un eventuale, successivo lavoro didattico (si veda l'attività 7).<br>**5.** I consigli per evitare il contagio. Si chiede ai bambini di ricordare ed elencare almeno tre consigli essenziali per evitare il contagio.	**Cap. 2**	Immaginativa<br><br>Cognitiva

Generare pensieri anticipatori positivi e riconnettere i bambini con la comunità scolastica.	**6.** Di nuovo assieme a scuola.<br>Si chiede ai bambini di elencare alcuni amici di scuola e le attività preferite che si riprenderà a svolgere assieme.	**Cap. 3**	Socio-relazionale ed emotiva
Conoscere i microrganismi: usare le proprie risorse cognitive per comprendere le dinamiche della vita microbica.	**7.** Video "Conoscere i microrganismi". Sono disponibili alcune risorse video, da usare nelle settimane successive al rientro, utili a fornire un supporto visivo ai contenuti del capitolo 1 e che possono essere usate dagli insegnanti di scienze per pianificare una lezione in classe.	**Cap. 1**	Cognitiva

Istruzioni didattiche per l'uso delle schede

Scheda 1. ***Disegna un momento di queste giornate.***

L'attività di elaborazione socio-emotiva dei vissuti connessi all'esperienza del coronavirus si apre con la richiesta agli alunni di indicare un momento particolare del tempo che hanno trascorso in famiglia. La richiesta è volutamente neutra (non si dice se il momento deve essere bello o brutto), perchè così facendo il bambino sarà più libero di scegliere l'orientamento da dare al suo lavoro. L'insegnante potrà utilizzare alcuni indizi del disegno (il tipo di scena rappresentata, l'espressione emotiva dei visi, lo stato d'animo dei personaggi) per farsi un'idea di come i bambini avranno percepito il loro tempo in famiglia. Chi volesse avere indicazioni più analitiche su come esaminare il disegno può riferirsi al lavoro di Bombi e colleghi (2007).

Scheda 2. ***I Pensieri di Marco e Maria.***

Questa scheda lavora sugli aspetti emotivi. Serve a esprimere pensieri e preoccupazioni che si sono avute nei giorni di allarme, di interruzione delle attività e/o di isolamento. La scheda usa una metodologia "proiettiva", nel senso che ai bambini non viene chiesto di parlare direttamente di loro in prima persona, ma piuttosto di assegnare preoccupazioni ed emozioni ai personaggi mediatori della scheda (Marco e Maria). Tale scheda può poi essere condivisa in gruppo. È possibile che durante la condivisione i bambini inizino gradualmente a parlare di sé. Se questo avviene in modo spontaneo, è utile lasciare scorrere la conversazione liberamente. È tuttavia importante far seguire subito dopo la scheda 3.

Scheda 3. ***Quando sono preoccupato***

Questa scheda serve a portare l'attenzione da un personaggio "esterno" sulla propria persona. Si chiede ai bambini di elencare alcune loro preoccupazioni (non necessariamente circoscritte all'allarme per l'epidemia di Covid-19), ma soprattutto li si porta a riflettere sulle loro strategie di coping e sulla relazione di aiuto. Questi ultimi

due aspetti sono più importanti della paura stessa. Infatti la letteratura in materia sottolinea come i problemi e le difficoltà del soggetto non siano legate solo all'oggetto che incute paura, ma siano soprattutto collegati al senso di solitudine e inadeguatezza che si può provare quando ci si scopre soli e senza risorse per affrontare i problemi (Binetti, Ferrazzoli, & Flora, 1999). Nella fase di condivisione dei risultati è quindi importante sottolineare soprattutto le indicazioni di coping e quelle relative alle persone che possono essere di aiuto. Come espansione di questa attività si potrebbe anche riprodurre la scheda su di un cartellone riportando, accanto ai problemi più comuni che emergono in classe, le strategie ritenute più utili per affrontarli.

Scheda 4. ***Disegno il coronavirus*** questa scheda sposta il lavoro sul piano cognitivo. Dopo aver creato un contenimento emotivo e una riflessione sul coping, si aprono spazi per consentire un'operatività cognitiva.

La scheda 4 serve a vedere quale immagine mentale abbia il bambino del coronavirus. Potrà poi essere utile confrontare tale immagine con gli stadi evolutivi della comprensione della malattia presentati nel capitolo 2 del testo (La comprensione della malattia nel bambino).

Scheda 5. ***I consigli per evitare il contagio*** La scheda 5 è utile per verificare se la campagna mediatica di prevenzione del contagio ha raggiunto anche i bambini in età scolare e in quale forma. L'insegnante avrà così modo di rafforzare gli eventuali messaggi corretti ed eventualmente correggere informazioni imprecise o inesatte, anche con l'ausilio del video informativo prodotto da ebookscuola (si veda il capitolo Cosa sono i microrganismi e il video informativo prodotto da ebookscuola).

In ogni caso è opportuno mantenere un atteggiamento equilibrato rispetto ai microrganismi e alle misure di prevenzione del contagio. Se è vero che le norme igieniche vanno sempre osservate, bisogna anche ricordare che la maggior parte dei microorganismi che vivono sopra e dentro il nostro corpo hanno un effetto positivo per la nostra salute. La ricerca di un comportamento salutare non può diventare un'ossessione verso la presenza di polvere o per il contatto con gli altri.

Scheda 6. ***Di nuovo insieme a scuola***

Infine, la scheda 6 serve a generare quei pensieri anticipatori positivi e a riconnettere i bambini con la comunità scolastica, con gli amici e i compagni di scuola. Ora che si è a scuola si può iniziare a ripensare alla relazione con gli altri, alle attività di gioco e studio da svolgere assieme nelle prossime settimane.

Attività 7. ***Proiezione e commento del video "Conoscere i microrganismi"***

Il video "Conoscere i microrganismi", la cui traduzione italiana è stata effettuata da ebookscuola su licenza del Cincinnati Children's Hospital Medical Center, può essere usato nelle settimane successive al rientro per rinforzare le informazioni utili a

mantenere comportamenti di tutela. È bene comunque non esagerare con i messaggi minacciosi al fine di non generare nuova ansia nei bambini. Il video può comunque fare da supporto a una lezione sui microrganismi, sulla loro diffusione e sui corretti comportamenti igienici. Il filmato è disponibile al link: https://www.ebookscuola.com/microrganismi.

Bibliografia dell'appendice

Binetti, P., Ferrazzoli, F., & Flora, C. (1999). *Ho paura : che cosa spaventa i bambini : un modo per conoscere e capire le loro paure*. Roma: Ma. Gi.

Bombi, A. S., Pinto, G., & Cannoni, E. (2007). P*ictorial Assessment of Interpersonal Relationships (PAIR)*. Firenze, Italy: Firenze University Press.

Anteprima delle schede

Scaricabile presso: https://www.ebookscuola.com/librettorientrocovid

Disegna un momento di queste giornate

Scheda 1

In questi giorni hai sentito parlare del Coronavirus e sarai dovuto rimanere a casa.
Disegna un momento che ti è rimasto in mente di questo periodo a casa.

La tua classe: ____ Quanti anni hai? ____ Sei un: ☐ Maschio oppure una ☐ Femmina

Adesso mostra il disegno ad un tuo compagno o compagna; ci sono delle somiglianze? E delle differenze?

I pensieri di Marco e Maria

Scheda 2

Marco e Maria hanno sentito parlare di Coronavirus dai loro genitori e in televisione e hanno diversi pensieri.
Cosa stanno pensando? (Puoi mettere parole o immagini)

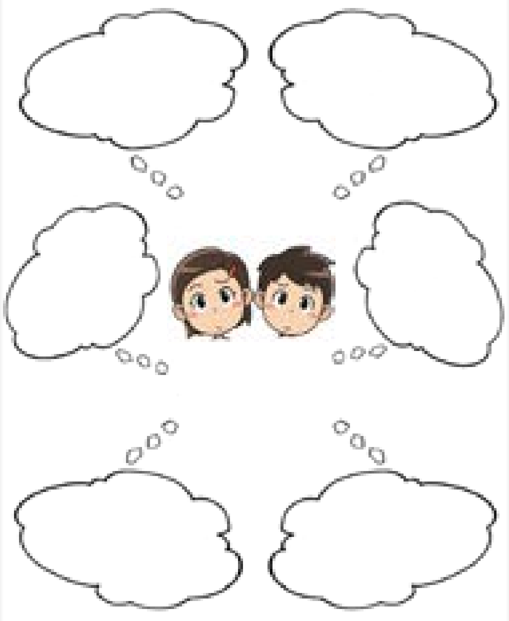

Materiale didattico allegato al testo "Accogliere bambini in classe dopo l'emergenza coronavirus" rilasciato con licenza Creative Common BY-NC-ND

Quando sono preoccupato

Scheda 3

E a te succede di essere preoccupato
o di avere dei problemi? Scrivi:

- Nella prima colonna delle cose che ti preoccupano o dei problemi che ti capita di avere
- nella colonna centrale indica delle cose che fai per farti passare i cattivi pensieri
- nell'ultima scrivi il nome di alcune persone che ti aiutano quando sei preoccupato

QUANDO SONO PREOCCUPATO...

Le mie preoccupazioni o i miei problemi	Cosa faccio per risolverli	Chi mi può aiutare

Quante cose puoi fare per risolvere i tuoi problemi o le tue preoccupazioni?

Contale e scrivi qui il totale: ____

Adesso racconta cosa hai scritto al tuo vicino di banco. Avete delle preoccupazioni in comune? E delle cose che fate per stare meglio che si assomigliano?

Come immagini il coronavirus? Disegnalo!

Scheda 4

In questi giorni hai sentito parlare del Coronavirus. Tu come lo immagini? Disegnalo qui.

La tua classe: ____ Quanti anni hai? ____ Sei un: ☐ Maschio oppure una ☐ Femmina

Adesso mostra il disegno ad un tuo compagno o compagna; ci sono delle somiglianze? E delle differenze?

[illegible]

Che sbadata!

Scheda 5

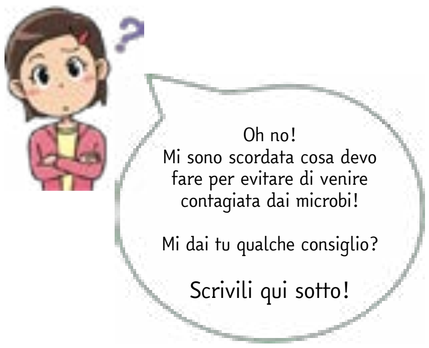

Scrivi tre regole importanti per non venire contagiati dai virus:

1. ____
2. ____
3. ____

Materiale didattico allegato al testo "Accogliere bambini in classe dopo l'emergenza coronavirus" rilasciato con licenza Creative Common BY-NC-ND

Di nuovo insieme a scuola

Scheda 6

È bello ritrovarsi tutti assieme!
Scrivi sulla giostra il nome di alcuni tuoi compagni:

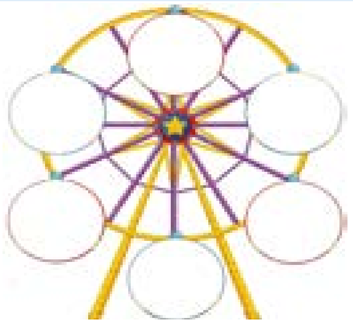

Scrivi sulla mongolfiera le cose che farete assieme a scuola nei prossimi giorni:

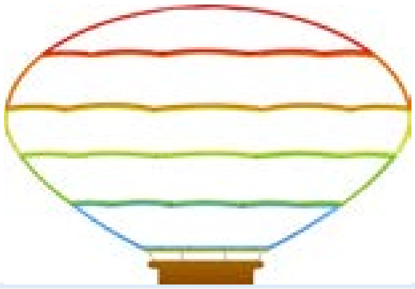

Materiale didattico allegato al testo "Accogliere bambini in classe dopo l'emergenza coronavirus" rilasciato con licenza Creative Common BY-NC-ND

Questionario per la valutazione delle attività (QVA/Covid)

Adesso che hai finito di fare queste schede, ci piacerebbe sapere se le hai gradite.

Ti chiediamo di leggere con attenzione ogni domanda e segnare con una croce la risposta che più si avvicina al tuo modo di pensare di questo momento, cioè quella con cui ti trovi più d'accordo.

	PER NIENTE	POCO	COSÌ-COSÌ	ABBASTANZA	MOLTO
1. Mi è piaciuto fare queste schede	○	○	○	○	○
2. Queste attività mi hanno aiutato a conoscere meglio i miei compagni	○	○	○	○	○
3. Facendo queste attività, ho imparato come affrontare meglio i miei problemi o le mie preoccupazioni	○	○	○	○	○
4. Facendo queste schede, mi sono sentito/a coinvolto/a ed ho ascoltato gli altri	○	○	○	○	○
5. Se ne avessi la possibilità, mi piacerebbe fare altre attività simili a queste.	○	○	○	○	○

Quale è l'attività che ti è piaciuta di più?

Le attività che hai fatto si potrebbero migliorare? Come?

Materiale didattico allegato al testo "Accogliere bambini in classe dopo l'emergenza coronavirus" rilasciato con licenza Creative Common BY-NC-ND

Appendice 2

Energisers: coltivare le relazioni nel gruppo classe

Michele Capurso

Le schede che seguono possono essere usate nei giorni successivi al rientro a scuola per delle piccole pause rigeneranti durante la mattinata. Servono a rafforzare il legame del gruppo classe, riscoprendo lo spirito di gruppo e la complicità tra gli alunni.

Gli energisers sono brevi esercizi che si usano per potenziare e facilitare dinamiche positive all'interno di gruppi di lavoro di qualsiasi tipo. Il loro scopo è proprio quello di creare uno spirito di gruppo, aiutando gli studenti a sentirsi a proprio agio e ad approfondire la conoscenza reciproca. Spesso gli energisers lavorano con la parte creativa della nostra mente, infondendo appunto energia e motivazione senza i vincoli della razionalità e del giudizio.

In questa sezione presentiamo sei esempi di energisers da usare in classe, nei giorni e nelle settimane successive al rientro dal lungo isolamento causato dall'emergenza del nuovo coronavirus. Le attività di questa sezione si possono usare in momenti diversi: all'inizio della giornata, per fare una pausa quando si vede un calo di attenzione, o come esercizio di chiusura al termine di una mattinata o settimana impegnativa. A differenza delle proposte contenute nell'allegato 1, che seguono una precisa logica progressiva, gli esercizi di questa sezione non si basano su di un ordine particolare. Sarà l'insegnante a decidere l'esercizio e il momento più opportuno per proporlo. Quelli che presentiamo sono solo degli esempi, ma ogni docente potrà inventarne di nuovi o cercare sul web altri energisers da fare con la propria classe.

Una copia stampabile in A4 delle schede da svolgere in classe è **scaricabile presso** https://www.ebookscuola.com/energisers

Esercizio 1. Analogie e similitudini

Obbiettivi: usare il pensiero narrativo e immaginativo per creare delle similitudini e delle analogie pensando alla propria classe.

Materiali per ciascun gruppo: una scheda con le carte delle analogie (scaricabile da https://www.ebookscuola.com/energisers). Un foglio di quaderno per ogni alunno per descrivere le motivazioni per ogni analogia.

Istruzioni: dividere la classe in gruppi di 3-4 persone, distribuire ad ogni gruppo una delle schede con le analogie, che vanno ritagliate per formare ciascuna un mazzo di 6 carte.

Svolgimento: a turno, ciascun componente del gruppo pesca una delle carte con le analogie. Il gruppo deve poi trovare una spiegazione condivisa per l'analogia pescata e scriverla sul foglio. Quando tutti i partecipanti hanno pescato la carta, si leggono le analogie e la spiegazione fornita alla classe. Se vi sono carte che sono capitate a più gruppi, la classe sceglie la spiegazione ritenuta più bella. È anche possibile riportare le analogie e le spiegazioni su un cartellone.

Esercizio 2. Due verità, una bugia.

Un esercizio divertente, che unisce la creatività alla conoscenza reciproca.

Obbiettivo: Approfondire la conoscenza reciproca degli alunni, rendere ciascuno protagonista davanti alla classe.

Materiali: un foglio di carta e una penna per ciascuno studente.

Descrizione: ciascuno studente deve raccontare due verità su di sé e una bugia. I compagni devono scoprire quale sia la bugia. L'attività si può svolgere a piccoli gruppi o con tutta la classe in plenaria. È anche possibile trasformarla in una gara a punti (si riceve 1 punto per ogni bugia riconosciuta).

Svolgimento: ogni studente deve pensare a tre racconti che lo riguardano. Possono riguardare eventi che gli sono accaduti, oppure situazioni che riguardano la propria vita attuale o passata. Due di questi racconti devono corrispondere a verità, uno invece deve essere completamente falso.

[Adattato da: https://www.bookwidgets.com/blog/2016/10/15-fun-classroom-energisers-for-students]

Esercizio 3. Tombola musicale con le parole chiave.

Questo gioco consiste nel cercare un testo di una canzone che contenga alcune parole chiave collegate alla lezione che si è appena svolta.

Obiettivi: rinforzare gli apprendimenti della giornata con un'attività piacevole e divertente.

Gli alunni si ricorderanno molto più facilmente i concetti appresi se potranno collegarli a un brano musicale a loro già noto.

Materiali: un set di carte contenenti alcune parole chiave relative alla lezione che si è appena svolta; un dispositivo per l'accesso ad internet per ogni gruppo di alunni.

Descrizione: si dividono gli alunni in piccoli gruppi (anche in base al numero di dispostivi collegati ad internet disponibili in classe). È anche possibile concedere l'uso di smartphone personali per questa attività.

Svolgimento: l'insegnante estrae una carta la parola chiave (ad esempio: "aggettivo" oppure "vulcano"). Gli alunni debbono cercare su internet un testo di una canzone che contenga tale termine. La squadra che trova per prima la canzone, vince.

È poi possibile cercare l'intero brano da far sentire alla classe. Una seconda variante può essere questa: valgono solo canzoni "uniche", che non siano già state trovate da altri. Una variante ulteriore può essere quella di cercare solo termini e canzoni in inglese. In questo caso è utile aggiungere la parola "lyrics" come chiave di ricerca.

[Adattato da: https://www.bookwidgets.com/blog/2016/10/15-fun-classroom-energisers-for-students]

Esercizio 4. In fila per….

Una attività dinamica e divertente da svolgere in palestra

Obiettivi: conoscere in modo più approfondito i propri compagni di classe.

Fare attività fisica.

Materiali: nessuno, ma l'attività si deve svolgere in uno spazio sufficientemente ampio (palestra o giardino).

Descrizione e svolgimento: l'insegnante da un comando che contiene un dato criterio ordinatore, e gli alunni debbono mettersi in riga sulla base di dato criterio.

Es.: "Mettetevi in riga in base all'altezza."; oppure "Dalla A alla Z, in base al vostro cognome". È anche possibile associare al criterio ordinatore un comando motorio "Fate un giro di corsa della palestra e poi …".

Ecco alcune idee simpatiche da usare come criterio ordinatore, anche in base all'età dei partecipanti: "in base alla distanza tra la vostra casa e la scuola"; "in base alla data di nascita"; "in base al peso del vostro zaino"; "in base al nome della vostra squadra del cuore", ecc.

Esercizio 5. Circolo dei complimenti

Obbiettivo: dare e ricevere complimenti con il resto della classe.

Materiali: Una scheda dei complimenti per ogni alunno (scaricabile da https://www.ebookscuola.com/energisers).

Svolgimento: dividere la classe in gruppi di massimo 10 studenti. Ogni alunno scrive il suo nome sulla scheda, poi la passa ad un compagno di banco. Il compagno di banco compila la prima riga del messaggio e la passa al compagno successivo che compila la seconda riga e così via. Quando tutte le schede sono tornate ai singoli proprietari, i bambini leggono i complimenti ricevuti.

Note: l'insegnante può decidere se condividere o mantenere riservati i complimenti, anche in base al clima emotivo che si creerà in classe.

Esercizio 6. Immagina e rispondi

Obiettivo: riscoprire l'immaginazione e la dimensione della condivisione come parti funzionanti della propria persona. Incoraggiare l'espressione del pensiero creativo.

Materiali: è necessario distribuire una copia della scheda “immagina” ad ogni partecipante (scaricabile da https://www.ebookscuola.com/energisers).

Svolgimento: chiedete agli alunni di rispondere individualmente alle domande della scheda. Dopo circa 5/10 minuti riunite gli alunni a piccoli gruppi e chiedete loro di condividere le risposte e spiegarle agli altri.

[Adattato da Ukens, L. L. (1996). Getting Together: Icebreakers and Group Energisers. San Francisco, CA: Wiley]

Anteprima delle schede

Scaricabile presso: https://www.ebookscuola.com/energisers

ALLEGATO 2

ebookscuola.com

scheda per l'energiser Es1

La nostra classe è come UNA FORESTA perché …	La nostra classe è come UN ARCOBALENO perché …
La nostra classe è come UN UOVO DI PASQUA perché …	La nostra classe è come UNA NAVE perché …
La nostra classe è come UN GELATO perché …	La nostra classe è come UN AEROPORTO perché …

Materiale didattico allegato al testo "Accogliere bambini in classe dopo l'emergenza coronavirus" rilasciato con licenza Creative Common BY-NC-ND

ebookscuola.com

scheda per l'energiser Es1

La nostra classe è come UN VULCANO perché …	La nostra classe è come UNA PASTICCERIA perché …
La nostra classe è come UNA PIZZA MARGHERITA perché …	La nostra classe è come UNA SCATOLA DI LEGO perché …
La nostra classe è come IL CIELO perché …	La nostra classe è come UN TRENO perché …

Materiale didattico allegato al testo "Accogliere bambini in classe dopo l'emergenza coronavirus" rilasciato con licenza Creative Common BY-NC-ND

ebookscuola.com

scheda per l'energiser Es5

Complimenti e messaggi positivi per:

(nome del destinatario dei messaggi)

Scrivi il tuo messaggio positivo per la persona indicata qui sopra	La tua firma

ebookscuola.com

scheda per l'energiser Es6

Immagina e rispondi

Usa la tua creatività e la tua fantasia per rispondere a queste domande.
Rilassati e divertiti: non esiste una risposta giusta o sbagliata!

1. Quanto è grande un desiderio?
2. Quale è il colore di questa giornata?
3. Che rumore fa l'abbraccio di chi ti vuole bene?
4. Cosa provi quando ascolti la tua canzone preferita?
5. Quale è il sapore della felicità?
6. Quanto pesa un litigio?
7. Quanto tempo dura un sorriso?
8. Quanto è lunga la solitudine?
9. Che forma ha l'amicizia?
10. Che valore ha stare assieme a scuola?

Microrganismi e azioni di tutela della salute

Presso la pagina https://www.ebookscuola.com/microrganismi sono disponibili risorse video liberamente utilizzabili in classe per rinforzare negli alunni la loro conoscenza dei microrganismi e i loro comportamenti di tutela sanitaria.

Tali risorse vanno utilizzate dopo aver svolto le attività proposte nell'allegato 1 del testo.

Note sugli autori

Livia Buratta, dott.ssa in Psicologia Clinica e della Salute, è dottoranda di ricerca in Scienze Umane presso il Dipartimento di Filosofia, Scienze Sociali, Umane e della Formazione dell'Università degli Studi di Perugia.

Michele Capurso, docente e ricercatore e in Psicologia dello Sviluppo e dell'Educazione, Direttore Scientifico di Ebookscuola.

Michele Capurso, docente di Psicologia dello Sviluppo e dell'Educazione presso l'Università di Perugia, si occupa da tempo di Psicologia Pediatrica e di educazione e comunicazione con bambini malati. Formatosi negli USA con i "Child Life Specialist" e diplomato counsellor a indirizzo rogersiano, è stato presidente di HOPE (Federazione Europea dei Pedagogisti Ospedalieri). È autore di articoli scientifici internazionali e diversi saggi sulle problematiche psicologiche e educative di bambini malati, tra i quali Gioco e studio in ospedale (Erickson, 2001), Facilitare la Comprensione della Malattia nel Bambino (Francoangeli, 2017), e, per i tipi MaGi, La casa delle punture (2005), Ti racconto il mio ospedale (2007), Quando si ammala un bambino (2008). Sul tema delle comunicazione ed educazione del bambino malato dirige la rivista internazionale Continuity in Education, da lui fondata nel 2019.

Giulia Cenci, Phd, psicologa, psicoterapeuta. Docente a contratto di Psicologia dello Sviluppo presso il Dipartimento di Filosofia, Scienze Sociali, Umane e della Formazione dell'Università degli Studi di Perugia.

Giulia Gizzi, psicologa, dottoranda in Etica della Comunicazione, della Ricerca Scientifica e dell'Innovazione Tecnologica presso il Dipartimento di Filosofia, Scienze Sociali, Umane e della Formazione dell'Università di Perugia.

Claudia Mazzeschi, Ph.D., professore ordinario in Psicologia Dinamica, è Direttore del Dipartimento di Filosofia, Scienze Sociali, Umane e della Formazione dell'Univer-

sità degli Studi di Perugia dove insegna Psicologia Dinamica e Modelli e strumenti per la valutazione psicologica presso il CdS in Valutazione del Funzionamento Individuale in Psicologia Clinica e della Salute. Si interessa di ricerca nell'area del parenting, delle relazioni di attaccamento, dei fattori di rischio e di protezione nei processi di sviluppo anche con riferimento agli aspetti di personalità.

Cristina Parrino, medico, specialista in Endocrinologia e Malattie del Metabolismo, Direttore Editoriale di EbookECM.

La dott.ssa Parrino ha esperienza nazionale e internazionale in ambito clinico-assistenziale, nella ricerca e nell'industria farmaceutica. Nel 2012 è stata Visiting Research presso la Temple University di Philadelphia, Stati Uniti, ed è autore di pubblicazioni scientifiche peer-reviewed su riviste internazionali. Si occupa di comunicazione scientifica, ricoprendo il ruolo di Section Editor della rivista online di Springer, Medici Oggi.

Dal 2019 presta la propria attività professionale a Roma come specialista ambulatoriale e collabora con l'Associazione Salutegiovani come medico scolastico presso Istituti Secondari.

Elisa Delvecchio, Ph.D., ricercatrice in Psicologia Dinamica, docente di Psicopatologia Generale e dello Sviluppo presso il CdS di Filosofia e Scienze e Tecniche Psicologiche e di Teorie e strumenti per la valutazione e progettazione dell'intervento in ambito familiare presso il CdS in Valutazione del Funzionamento Individuale in Psicologia Clinica e della Salute, Dipartimento di Filosofia, Scienze Sociali, Umane e della Formazione, Università di Perugia.
