# Supplementary material for: Empowering Children Through School Re-Entry Activities After the COVID-19 Pandemic
Source: Contin Educ. 2020 May 15;1(1):64–82. doi: 10.5334/cie.17 (PMC11104315; doi:10.5334/cie.17)
Supplement: Appendix A. — Training manual for all school teachers (in Italian; Capurso & Mazzeschi, 2020). [file cie-1-1-17-s1.zip › Accogliere i bambini in classe dopo emergenza coronavirus/Esercitazioni/esercitazione_1.pdf]

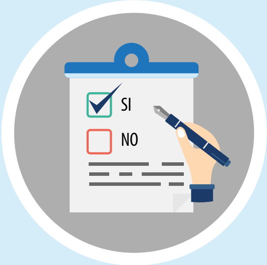

ESERCITAZIONE

DA COMPILARE USANDO ADOBE ACROBAT READER

ebookscuola.com

1

SVOLGIMENTO DELL'UNITÀ DI APPRENDIMENTO PER LA RIELABORAZIONE SOCIO-EMOTIVA DEI VISSUTI RELATIVI ALL'EMERGENZA CAUSATA DAL NUOVO CORONAVIRUS

Aiutare gli alunni a riprendere la scuola con una attività utile a rielaborare i vissuti socio-emotivi connessi all'esperienza relativa alla pandemia causata dal nuovo coronavirus.

COME SVOLGERE QUESTA ESERCITAZIONE:

1. Completa la lettura del libro.
2. Prepara un fascicolo per ogni alunno, stampando tutte le schede dell'unità di apprendimento *aiutare i bambini a rielaborare i vissuti emotivi* presentata nell'allegato 1 del testo e scaricabile in formato A4 fotocopiabile da: https://www.ebookscuola.com/librettorientrocovid.
3. Svolgi l'unità di apprendimento possibilmente il primo e secondo giorno dopo il rientro in classe al termine dell'emergenza causata dal nuovo coronavirus.
4. Compila questa scheda per fare un rapporto della tua attività.
5. Vai su ebookscuola.com e carica il file compilato nel tuo portfolio personale relativo a questo corso.

Attenzione: questa esercitazione prevede la riapertura delle scuole nel corrente anno scolastico. Qualora questa evenienza non si verificasse, il docente può com-pletare solo l'autocertificazione riportata nell'ultima pagina di questo documento e caricare il presente file nel portfolio del corso presente su ebookscuola.com, **impegnandosi comunque a completare il presente corso svolgendo l'attività didattica proposta** nell'allegato 1 del libro **alla prima occasione utile** e comunque nei giorni immediatamente seguenti al rientro a scuola.

SCUOLA OVE SI LAVORA	Seleziona...	CLASSE OVE SI È SVOLTA L'ATTIVITÀ	Seleziona...

PER VALUTARE L’ATTIVITÀ SVOLTA

Indica le attività che hai svolto in classe e la data di svolgimento, se la scuola non riprende prima di giugno, compilare l’auto-certificazione in ultima pagina.

SCHEDA	SVOLTA IN DATA
Scheda 1. Disegna un momento di queste giornate	<inserisci data di svolgimento>
Scheda 2. I Pensieri di Marco e Maria	<inserisci data di svolgimento>
Scheda 3. Quando sono preoccupato	<inserisci data di svolgimento>
Scheda 4. Disegna il coronavirus	<inserisci data di svolgimento>
Scheda 5. I consigli per evitare il contagio	<inserisci data di svolgimento>
Scheda 6. Di nuovo insieme a scuola	<inserisci data di svolgimento>
Attività 7. Proiezione e commento del video “conoscere i microrganismi”	<inserisci data di svolgimento>

RIPENSA ALLO SVOLGIMENTO DELLE DIVERSE ATTIVITÀ PRESENTATE NELL’ALLEGATO 1 DEL LIBRO E RISPONDI.

1. Quali sono gli aspetti che hanno funzionato bene nelle attività svolte?

2. Che voto daresti, globalmente, alla riuscita delle attività?

Seleziona...

ebookscuola.com

3. C'è stato qualcosa che non ha funzionato come ti saresti aspettato/a?

4. Per quale ragione pensi che questo aspetto non abbia funzionato bene?

5. Se dovessi rifare questa attività, cosa cambieresti per renderle più efficaci?

Alcuni ricercatori del dipartimento di scienze umane (FISSUF) dell'università degli studi di Perugia sono interessanti a ricevere le schede compilate dagli alunni nello svolgere la presente attività per scopi di ricerca. Tutte le informazioni verranno usate in forma completamente anonima e nel rispetto dei canoni etici della ricerca scientifica.
Se sei disponibile ad inviare copia di tale schede (anche a mezzo elettronico) ti chiediamo di seguire le indicazioni riportate nel sito: https://www.ebookscuola.com/blog/ricercacovid.

- ◯ Sì, sono disponibile a prendere contatto con i ricercatori dell'università di Perugia per sostenere la loro ricerca e visiterò la pagina web indicata.
- ◯ No, non sono disponibile a collaborare alla ricerca.

AUTOCERTIFICAZIONE DEL LAVORO SVOLTO:

Io **sottoscritto/a** ______________________

◯ dichiaro di aver svolto questa eserctazione e le attività didattiche ad essa collegate nei giorni ______________________

oppure

◯ dichiaro di non aver potuto svolgere questa esercitazione e le attività didattiche ad essa collegate nel corrente anno scolastico a causa della mancata ripresa dell'attività didattica nella mia zona. Mi impegno comunque a svolgere tali attività nei giorni immediatamente successivi alla ripresa delle attività didattiche, presumibilmente nel mese di ________ anno ________ .

COMPUTO NELL'UNITÀ FORMATIVA

MINUTI	TIPOLOGIA
150	Ricerca-azione: realizzazione
30	Ricerca-azione: documentazione

RITENGO CHE QUESTA ESERCITAZIONE SIA STATA:

◯ CHIARA/UTILE ◯ COSÌ COSÌ ◯ INCOMPRENSIBILE/INUTILE
