# Supplementary material for: Empowering Children Through School Re-Entry Activities After the COVID-19 Pandemic
Source: Contin Educ. 2020 May 15;1(1):64–82. doi: 10.5334/cie.17 (PMC11104315; doi:10.5334/cie.17)
Supplement: Appendix A. — Training manual for all school teachers (in Italian; Capurso & Mazzeschi, 2020). [file cie-1-1-17-s1.zip › Accogliere i bambini in classe dopo emergenza coronavirus/Schede didattiche/scheda_1.pdf]

ebookscuola.com

Disegna un momento di queste giornate

Scheda 1

In questi giorni hai sentito parlare del Coronavirus e sarai dovuto rimanere a casa.
Disegna un momento che ti è rimasto in mente di questo periodo.

La tua classe: ______ Quanti anni hai? ______ Sei un: ☐ Maschio oppure una ☐ Femmina

Adesso mostra il disegno ad un tuo compagno o compagna; ci sono delle somiglianze? E delle differenze?

Materiale didattico allegato al testo “Accogliere bambini in classe dopo l’emergenza coronavirus” rilasciato con licenza Creative Common BY-NC-ND

I pensieri di Marco e Maria

Scheda 2

Marco e Maria hanno sentito parlare di Coronavirus dai loro genitori e in televisione e hanno diversi pensieri. Cosa stanno pensando? (Puoi mettere parole o immagini)

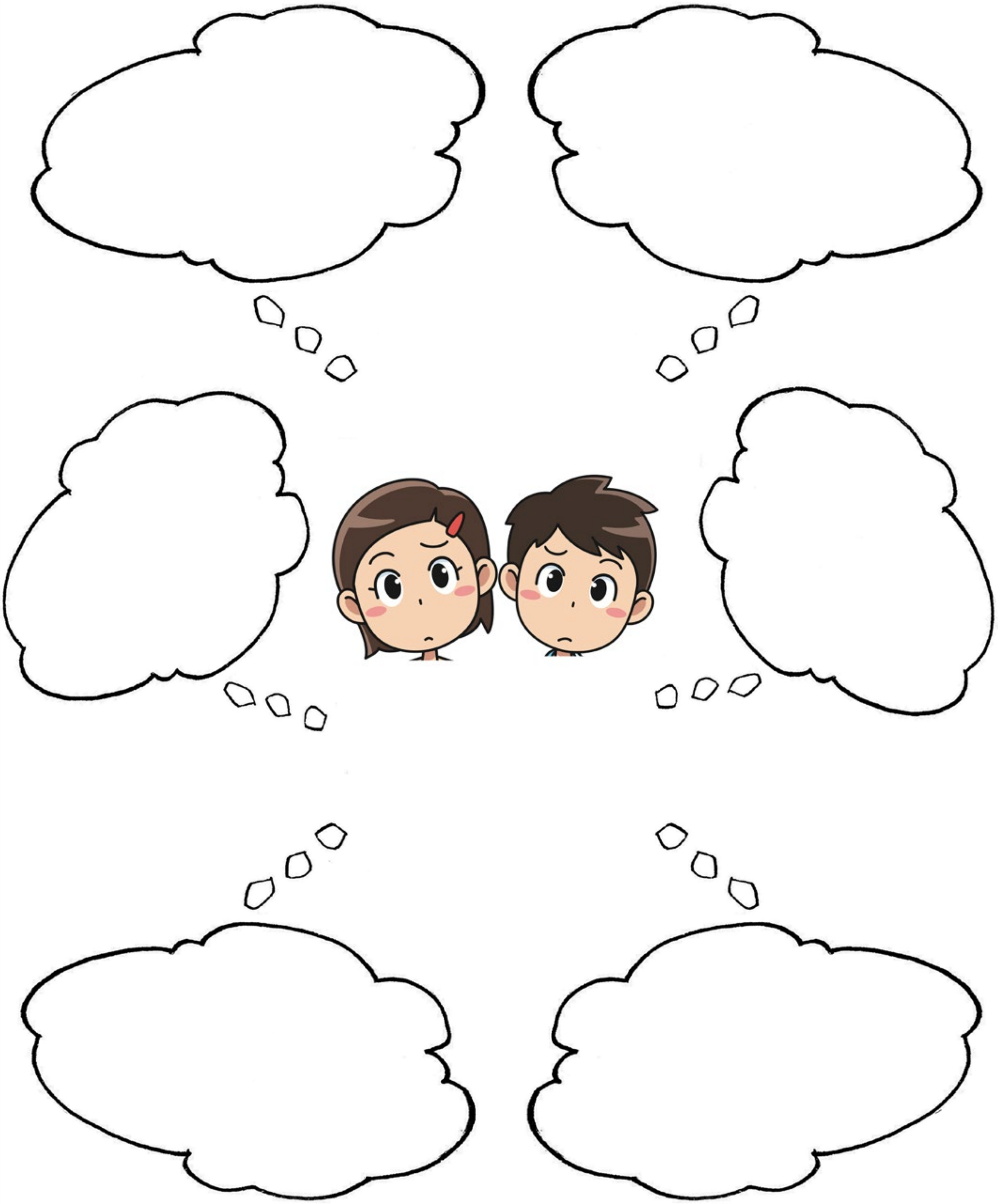

Materiale didattico allegato al testo “Accogliere bambini in classe dopo l’emergenza coronavirus” rilasciato con licenza Creative Common BY-NC-ND

Quando sono preoccupato

ebookscuola.com

Scheda 3

E a te succede di essere preoccupato
o di avere dei problemi? Scrivi:

- Nella prima colonna delle cose che ti preoccupano o dei problemi che ti capita di avere
- nella colonna centrale indica delle cose che fai per farti passare i cattivi pensieri
- nell'ultima scrivi il nome di alcune persone che ti aiutano quando sei preoccupato

QUANDO SONO PREOCCUPATO...		
Le mie preoccupazioni o i miei problemi	Cosa faccio per risolverli	Chi mi può aiutare

Quante cose puoi fare per risolvere i tuoi problemi o le tue preoccupazioni?

Contale e scrivi qui il totale:

Adesso racconta cosa hai scritto al tuo vicino di banco. Avete delle preoccupazioni in comune? E delle cose che fate per stare meglio che si assomigliano?

Materiale didattico allegato al testo "Accogliere bambini in classe dopo l'emergenza coronavirus" rilasciato con licenza Creative Common BY-NC-ND

ebookscuola.com

Come immagini il coronavirus? Disegnalo!

Scheda 4

In questi giorni hai sentito parlare del Coronavirus. Tu come lo immagini? Disegnalo qui.

La tua classe: ☐ Quanti anni hai? ☐ Sei un: ☐ Maschio oppure una ☐ Femmina

Adesso mostra il disegno ad un tuo compagno o compagna; ci sono delle somiglianze? E delle differenze?

Materiale didattico allegato al testo "Accogliere bambini in classe dopo l'emergenza coronavirus" rilasciato con licenza Creative Common BY-NC-ND

Che sbadata!

Scheda 5

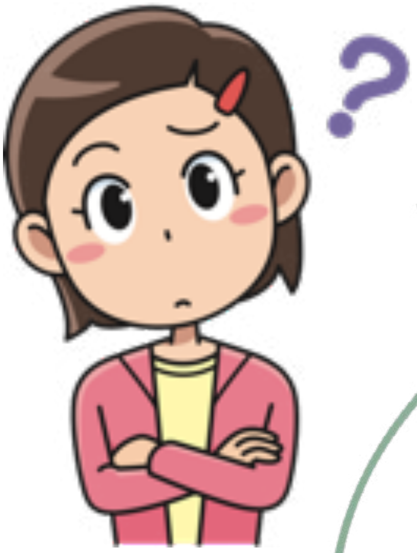

Oh no!
Mi sono scordata cosa devo fare per evitare di venire contagiata dai microbi!

Mi dai tu qualche consiglio?

Scrivili qui sotto!

Scrivi tre regole importanti per non venire contagiati dai virus:

1.

2.

3.

Materiale didattico allegato al testo "Accogliere bambini in classe dopo l'emergenza coronavirus" rilasciato con licenza Creative Common BY-NC-ND

Di nuovo insieme a scuola

Scheda 6

È bello ritrovarsi tutti assieme!
Scrivi sulla giostra il nome di alcuni tuoi compagni:

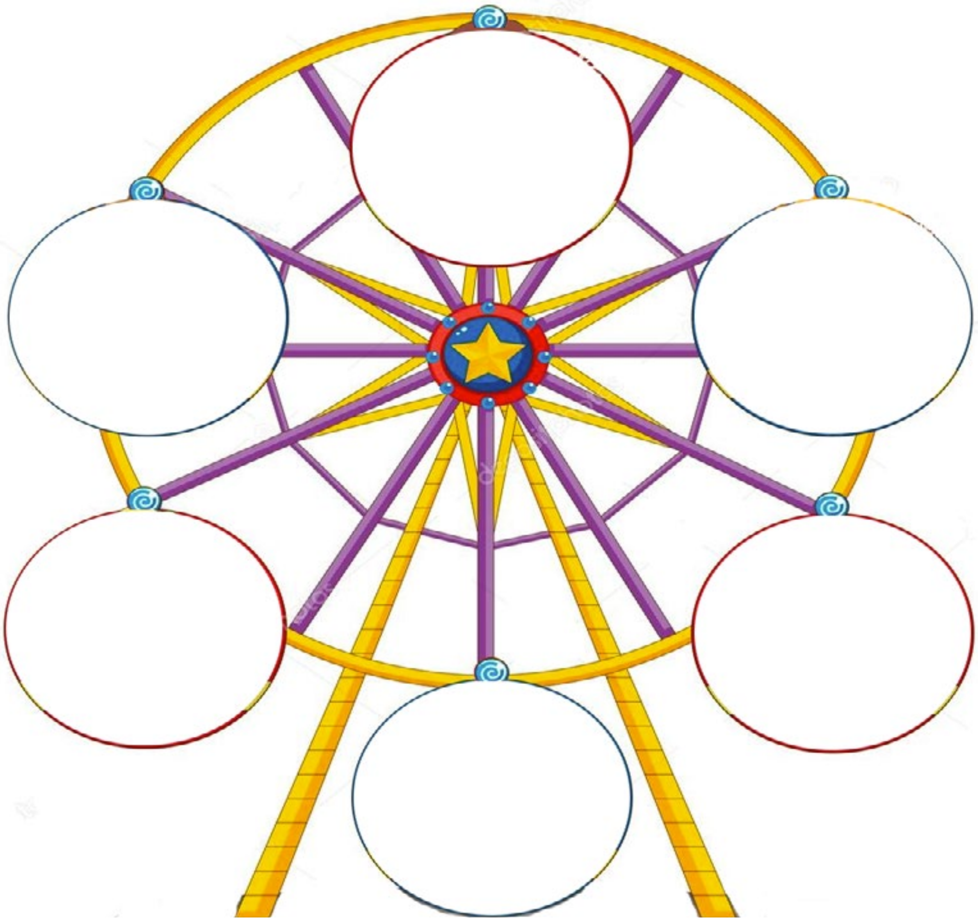

Scrivi sulla mongolfiera le cose che farete assieme a scuola nei prossimi giorni:

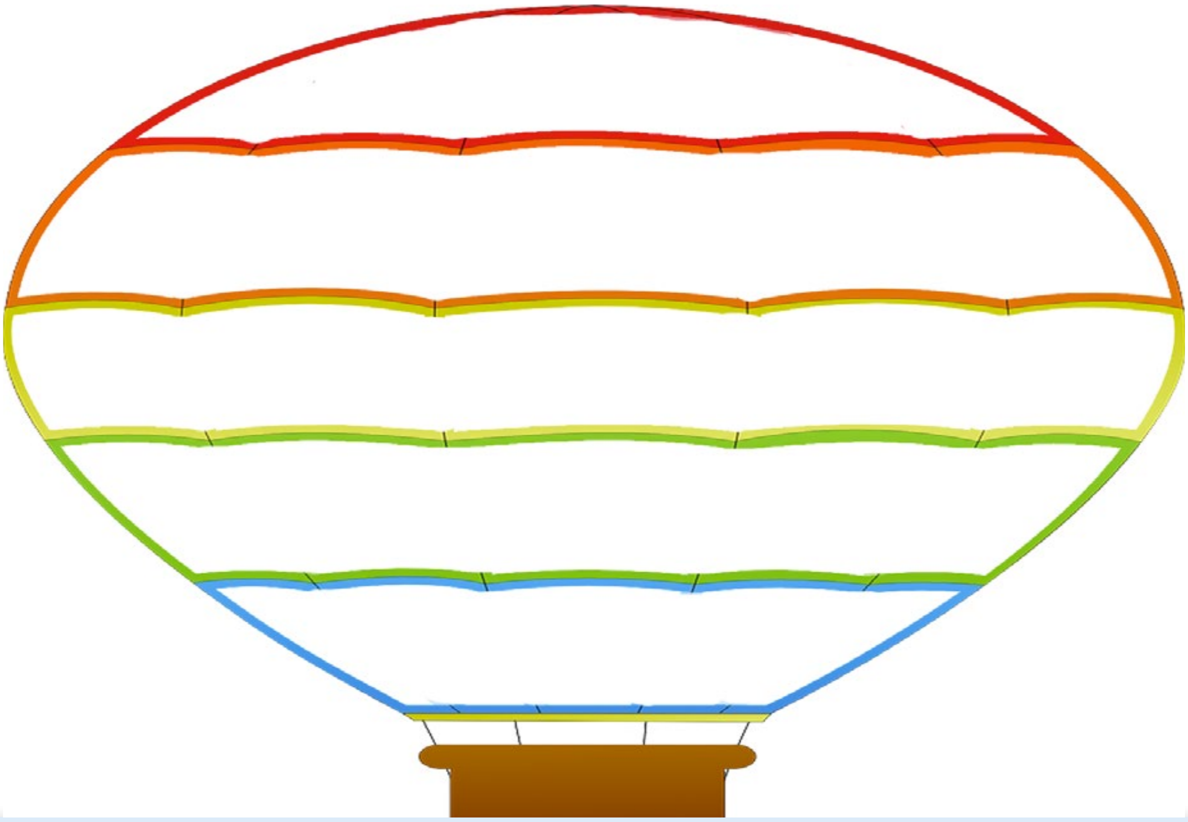

Materiale didattico allegato al testo "Accogliere bambini in classe dopo l'emergenza coronavirus" rilasciato con licenza Creative Common BY-NC-ND

Questionario per la valutazione delle attività (QVA/Covid)

ebookscuola.com

Adesso che hai finito di fare queste schede,
ci piacerebbe sapere se le hai gradite.

Ti chiediamo di leggere con attenzione ogni domanda e segnare con una croce la risposta che più si avvicina al tuo modo di pensare di questo momento, cioè quella con cui ti trovi più d'accordo.

	PER NIENTE	POCO	COSÌ-COSÌ	ABBASTANZA	MOLTO
1. Mi è piaciuto fare queste schede	○	○	○	○	○
2. Queste attività mi hanno aiutato a conoscere meglio i miei compagni	○	○	○	○	○
3. Facendo queste attività, ho imparato come affrontare meglio i miei problemi o le mie preoccupazioni	○	○	○	○	○
4. Facendo queste schede, mi sono sentito/a coinvolto/a ed ho ascoltato gli altri	○	○	○	○	○
5. Se ne avessi la possibilità, mi piacerebbe fare altre attività simili a queste.	○	○	○	○	○

Quale è l'attività che ti è piaciuta di più?

Le attività che hai fatto si potrebbero migliorare? Come?

Materiale didattico allegato al testo "Accogliere bambini in classe dopo l'emergenza coronavirus" rilasciato con licenza Creative Common BY-NC-ND
