# Supplementary material for: Empowering Children Through School Re-Entry Activities After the COVID-19 Pandemic
Source: Contin Educ. 2020 May 15;1(1):64–82. doi: 10.5334/cie.17 (PMC11104315; doi:10.5334/cie.17)
Supplement: Appendix B. — Activity pack for primary and middle school students. [file cie-1-1-17-s2.pdf]

ebookscuola.com

Draw a moment of these days

WS 1

In these days you have heard of the Coronavirus and you had to stay home.
Draw a moment that has remained in your mind from this period at home.

Your class: ______ how old are you? ______ You are: ☐ Male or ☐ Female

Now show the drawing to your partner and discuss. Are there any similarities? And differences?

Educational material connected to the book "Welcoming children in the classroom after the coronavirus emergency" released under the Creative Common license BY-NC by ebookscuola.com

John and Mary's thoughts

WS 2

John and Mary have heard of Coronavirus from their parents and on television and have different thoughts.
What are they thinking? (You can write words or draw pictures)

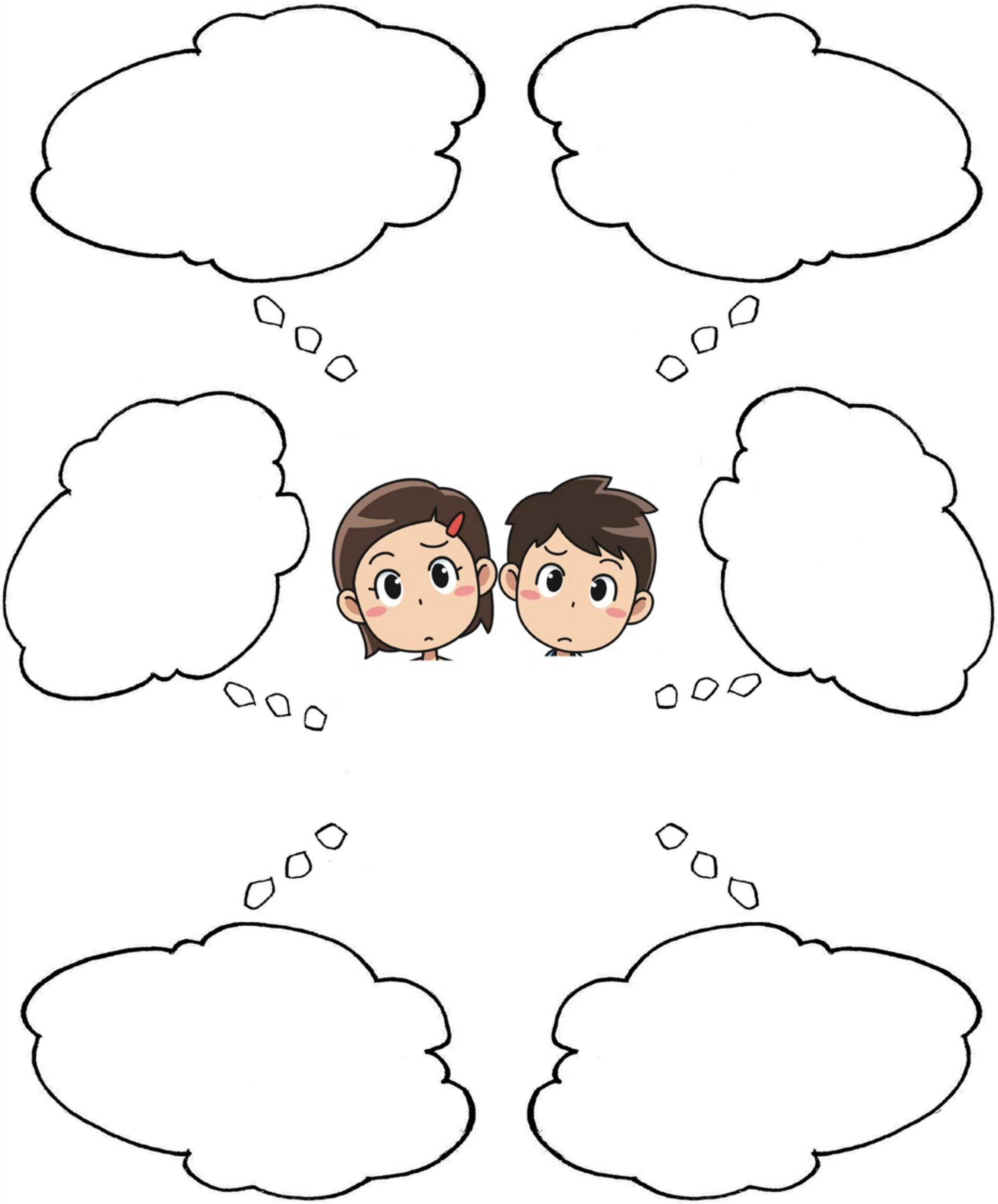

Educational material connected to the book "Welcoming children in the classroom after the coronavirus emergency" released under the Creative Common license BY-NC by ebookscuola.com

When I'm worried

ebookscuola.com

WS 3

Do you happen to be worried or
have problems? Write:

- In the first column, the things that worry you or the problems you happen to have;
- In the central column, indicate the things you do to get past bad thoughts, or to overcome your problem
- In the last column write the name of some people who may help you when you are worried.

WHEN I'M WORRIED ...		
My worries or my problems	What do I do to resolve them	Who can help me

How many things can you do to solve your problems or concerns?

Count them and write the total here: ____________

Now tell a classmate near you what you wrote. Do you have concerns in common? And what do you similarly do to feel better?

Educational material connected to the book "Welcoming children in the classroom after the coronavirus emergency" released under the Creative Common license BY-NC by ebookscuola.com

ebookscuola.com

How do you imagine the coronavirus? Draw it!

You have heard of Coronavirus these days. How do you imagine it? Draw it here.

WS 4

Your class: ____ How old are you? ____ You are: ☐ Male or ☐ Female

Adesso mostra il disegno ad un tuo compagno o compagna; ci sono delle somiglianze? E delle differenze?

Educational material connected to the book "Welcoming children in the classroom after the coronavirus emergency" released under the Creative Common license BY-NC by ebookscuola.com

ebookscuola.com

What a forgetful guy!

WS 5

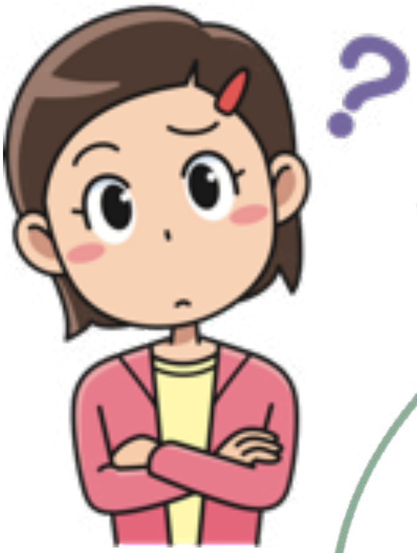

I forgot what to do to avoid getting infected by germs!

Can you give me some advice?

Write them below!

Write down three important rules for not getting infected by viruses:

1.

2.

3.

Educational material connected to the book "Welcoming children in the classroom after the coronavirus emergency" released under the Creative Common license BY-NC by ebookscuola.com

Back to school again

It's nice to be all together!!

Write the name of some of your mates on the carousel:

WS 6

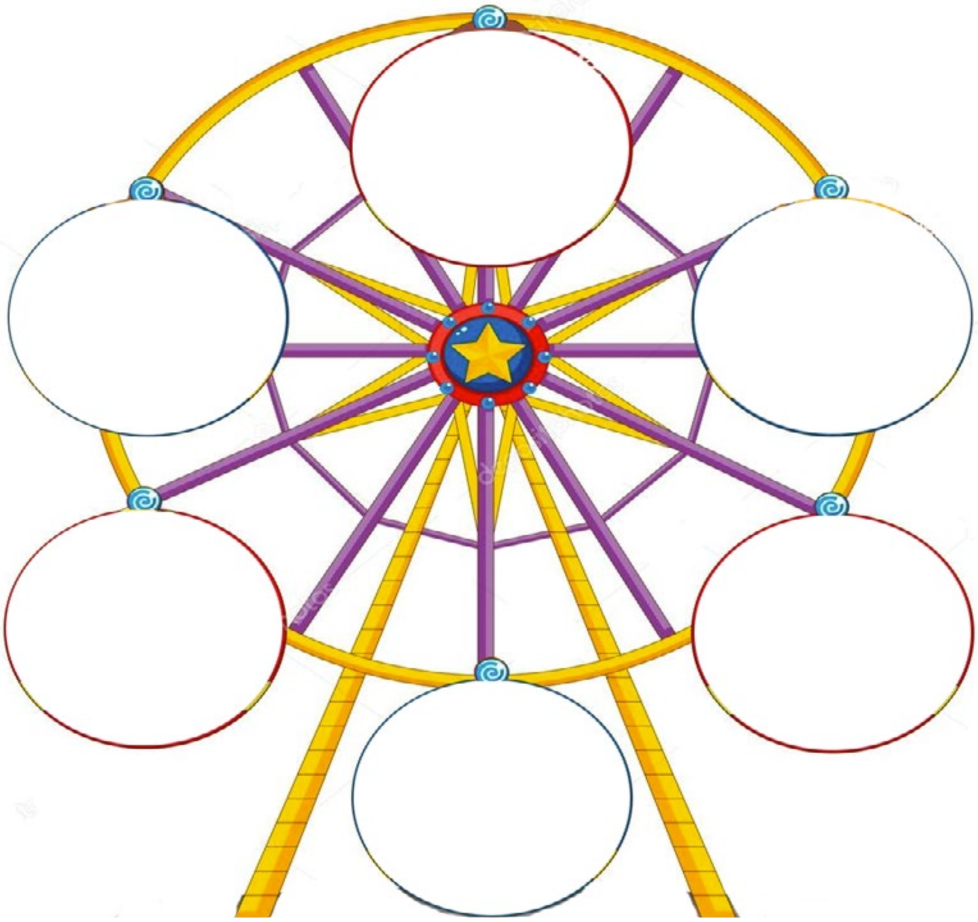

Write on the balloon the things you will do together in school in the next days:

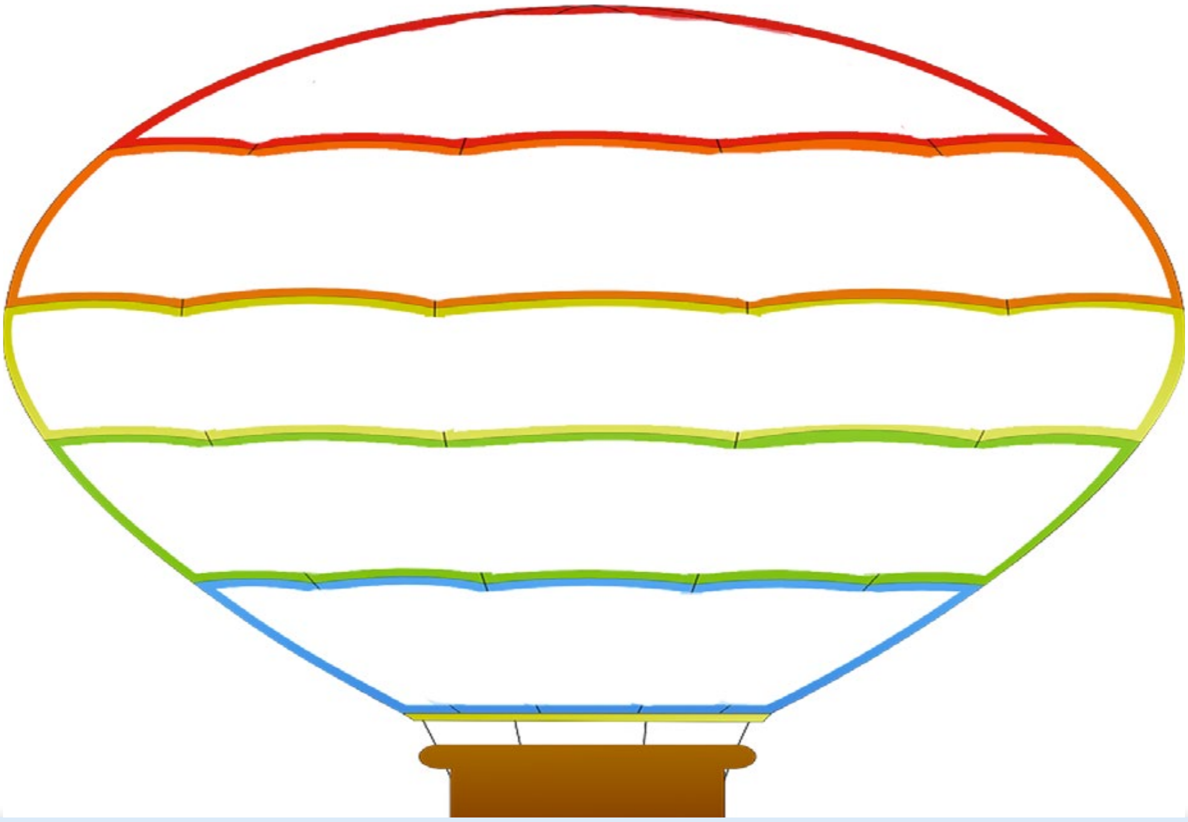

Educational material connected to the book "Welcoming children in the classroom after the coronavirus emergency" released under the Creative Common license BY-NC by ebookscuola.com

Activity Assessment Questionnaire (QVA / Covid)

Now that you are done filling out these sheets, we would like to know if you liked them.

Please carefully read each question and mark with a cross the answer that comes closest to your way of thinking right now, that is, the one with which you are most in agreement.

	NOT AT ALL	A LITTLE	SO-SO	SOME	VERY MUCH
1. I enjoyed completing these activities.	○	○	○	○	○
2. These activities helped me to get know my classmates better.	○	○	○	○	○
3. By doing these activities, I learned how to better deal with my problems or concerns.	○	○	○	○	○
4. I felt involved and listened to the others while doing these activities	○	○	○	○	○
5. If I had the chance, I would like to do other activities similar to these.	○	○	○	○	○

6. What activity do you like the most?

7. The activities that you did could be improved? How?

Educational material connected to the book "Welcoming children in the classroom after the coronavirus emergency" released under the Creative Common license BY-NC by ebookscuola.com
