# Supplementary material for: Empowering Children Through School Re-Entry Activities After the COVID-19 Pandemic
Source: Contin Educ. 2020 May 15;1(1):64–82. doi: 10.5334/cie.17 (PMC11104315; doi:10.5334/cie.17)
Supplement: Appendix C. — Activity pack for kindergarten pupils. [file cie-1-1-17-s3.pdf]

HANDOUT FOR ACTIVITY 3

Recognize these feelings and tell us when you felt that way

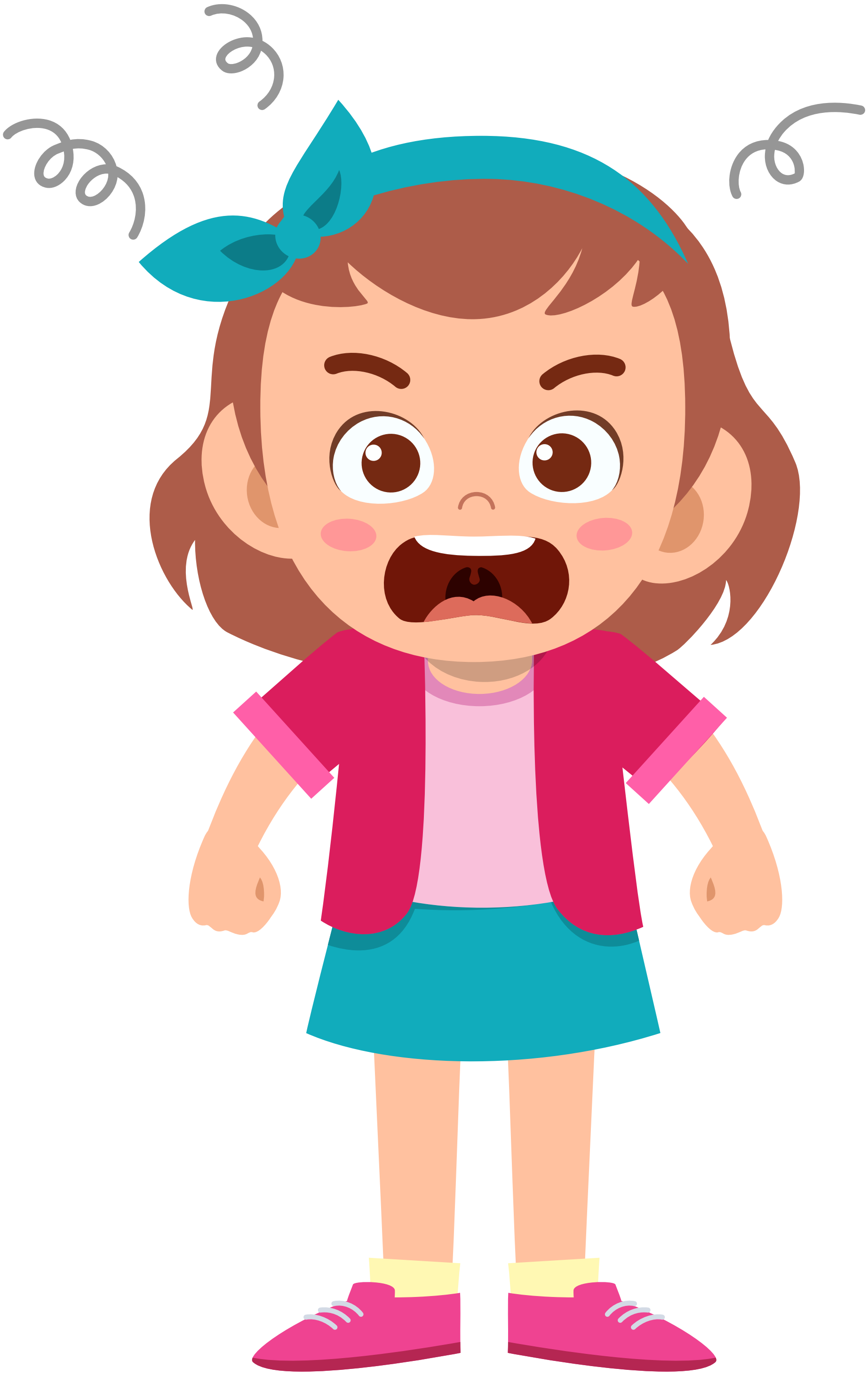

ANGRY

Educational material connected to the book "Welcoming children in the classroom after the coronavirus emergency" released under the Creative Common license BY-NC by ebookscuola.com

HANDOUT FOR ACTIVITY 3

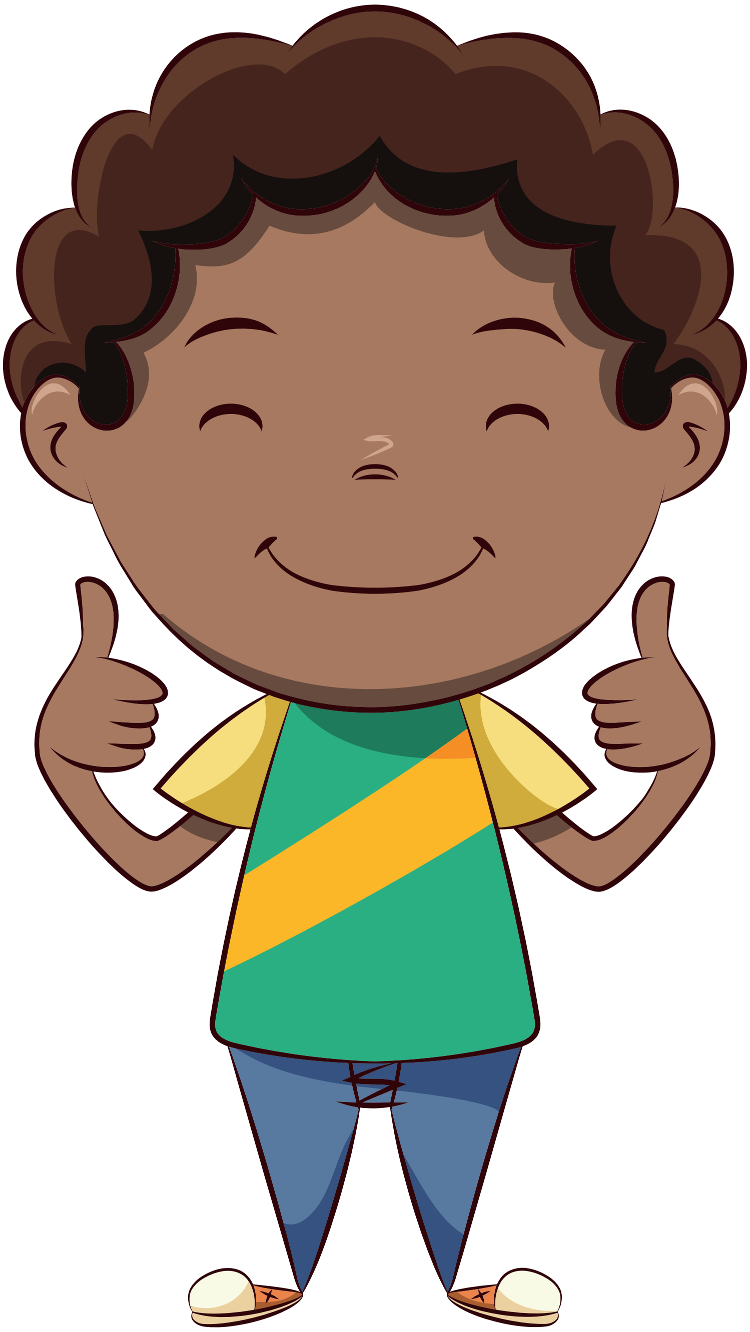

HAPPY

Educational material connected to the book "Welcoming children in the classroom after the coronavirus emergency" released under the Creative Common license BY-NC by ebookscuola.com

ebookscuola.com

HANDOUT FOR ACTIVITY 3

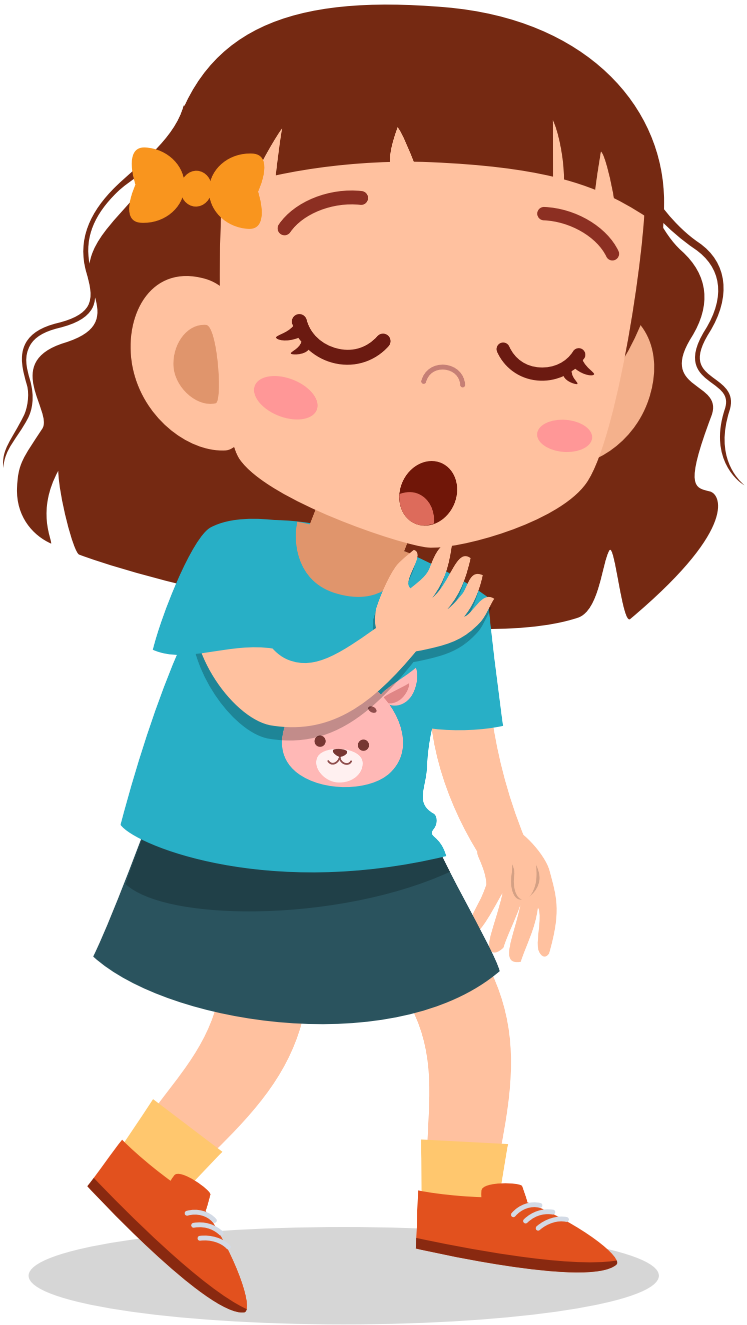

BORED

Educational material connected to the book "Welcoming children in the classroom after the coronavirus emergency" released under the Creative Common license BY-NC by ebookscuola.com

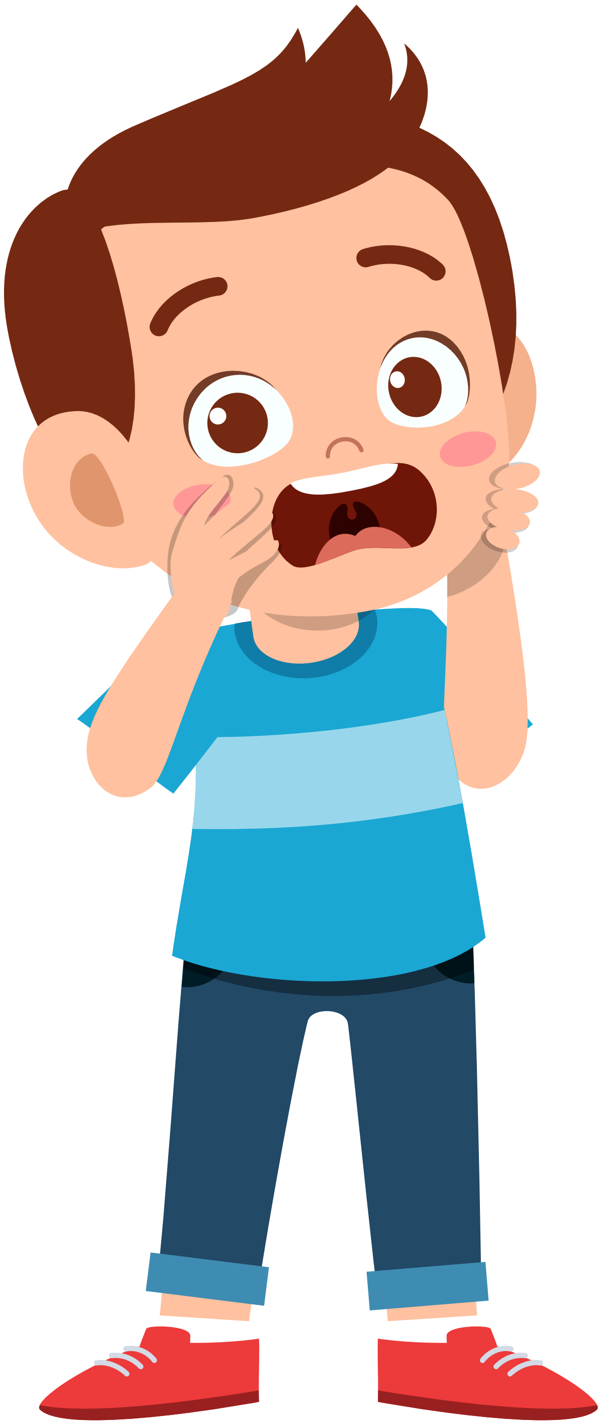

SCARED

Educational material connected to the book "Welcoming children in the classroom after the coronavirus emergency" released under the Creative Common license BY-NC by ebookscuola.com

HANDOUT FOR ACTIVITY 3

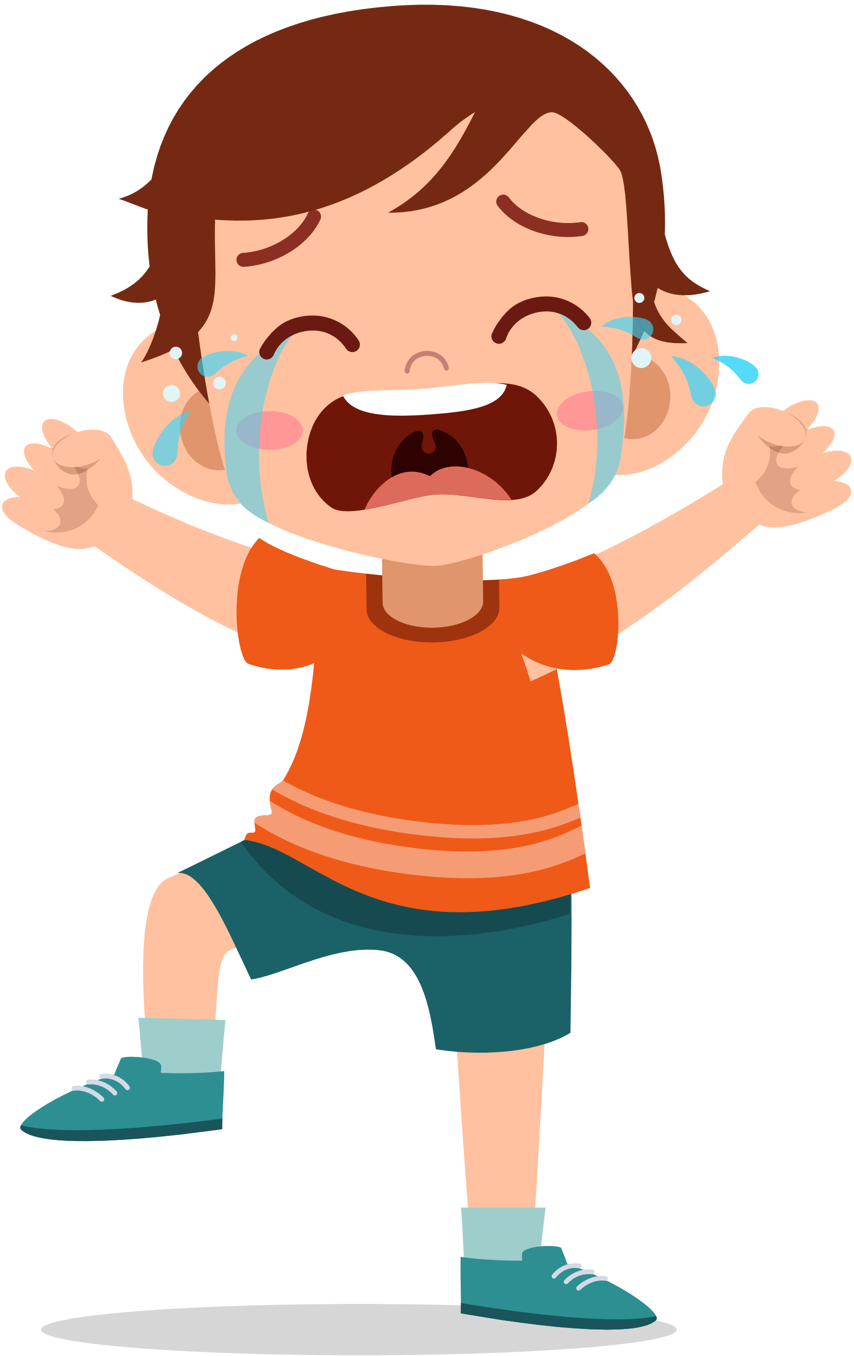

SAD

Educational material connected to the book "Welcoming children in the classroom after the coronavirus emergency" released under the Creative Common license BY-NC by ebookscuola.com

HANDOUT FOR ACTIVITY 5

How do you imagine the coronavirus? Draw it!

You have heard of Coronavirus these days. How do you imagine it? Draw it here.

Educational material connected to the book "Welcoming children in the classroom after the coronavirus emergency" released under the Creative Common license BY-NC by ebookscuola.com

At the School District

by Samantha Bonucci and Michele Capurso

English translation by Luciana Pagano Salmi; Adapted from *La Valle dei Mulini* by Noelia Blanco and Valeria Docampo Ed. Terre di Mezzo.

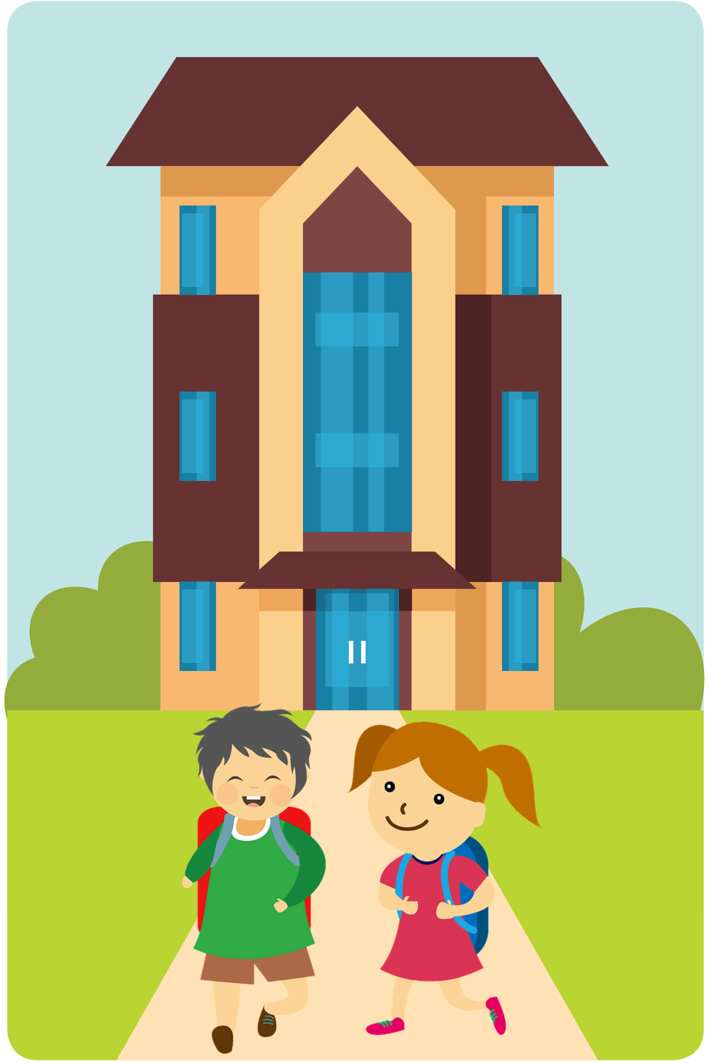

At the school district lived boys and girls, teachers that were always kind, cooks that prepared delicious food, ladies that made the school always shine.
The days went by in harmony while the children played, grew up and did many interesting activities.

Then one day, suddenly, arrived a nasty speckle called Coronavirus. Coronavirus jumped secretly from one person to the other, landing on their hands, nose and mouth, making most of the people it touched ill.

With the arrival of Mr. Corona, everything changed. The school was closed and empty: there was no more the laughter of the children, the embrace of the teachers, the exquisite smell of the juices that Giovanna the cook used to prepare.

To avoid Coronavirus people stayed inside their homes and so, in a moment, the embraces, the handshaking and even the bright smiles that where now hiding behind blue masks all disappeared from the district.

Locked inside the house, people no longer saw the shooting stars and even forgot to ask for their wishes. And so, little by little the inhabitants of the school district stopped dreaming.

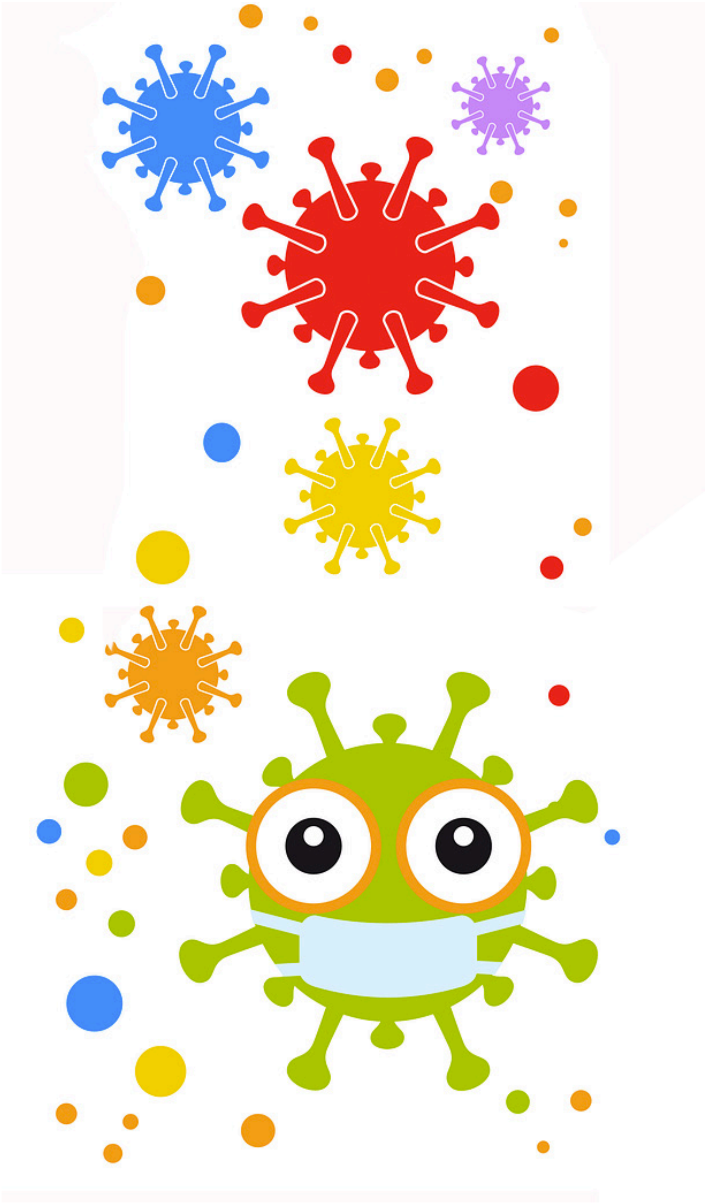

But then, one fine morning even the Wind did not blow because it felt useless. Without the children's voices there were no more messages to carry, without the cook who prepared the pasta with meat sauce there were no delicious smells to carry, without the teachers singing and telling stories there was nothing more to listen to.

The inhabitants of the neighborhood began to really feel the lack of everyone's presence: they could no longer feel happy.

Yet here and there, sleeping inside the heart of the School, you could find some hidden dreams.
One is that of the teacher Agnese, who dreams of embracing all the boys and girls soon. The teacher missed her children so much and in order not to feel alone, every now and then, she began to sing some songs and imagined seeing the children of her school again during roll call, at breakfast or while playing in the garden.

One night Agnese, the teacher, sees a strange silhouette that stands out in the distance where the school sleeps.
It is the silhouette of the Butterfly Girl
"What are you doing there?" Asked Agnese.

"I am trying to fly. I want to dance with the clouds, and see the world backwards ", replied the little butterfly girl.
"To fly?" the teacher replied with wonder.
"Yes! Everyday I try to build wings that can take me to the sky".
Agnese smiled.
"I have also kept my dreams! I will saw you a dress to fly."

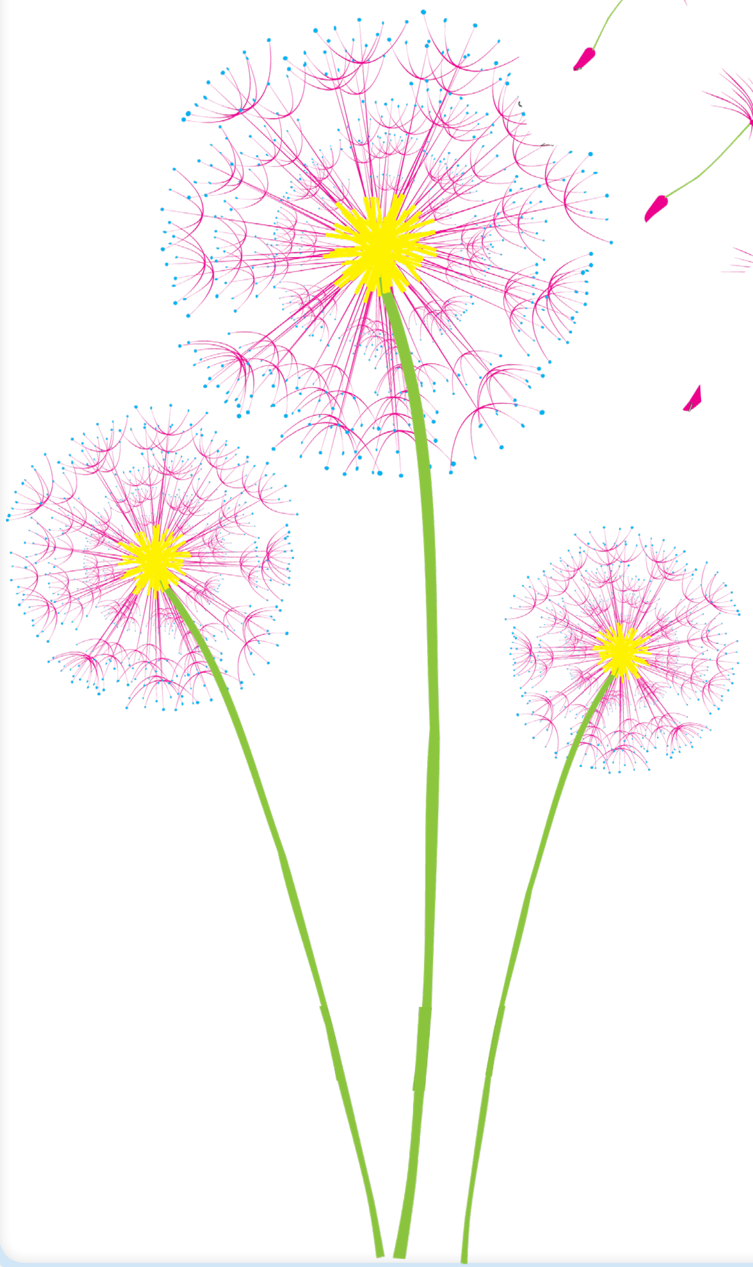

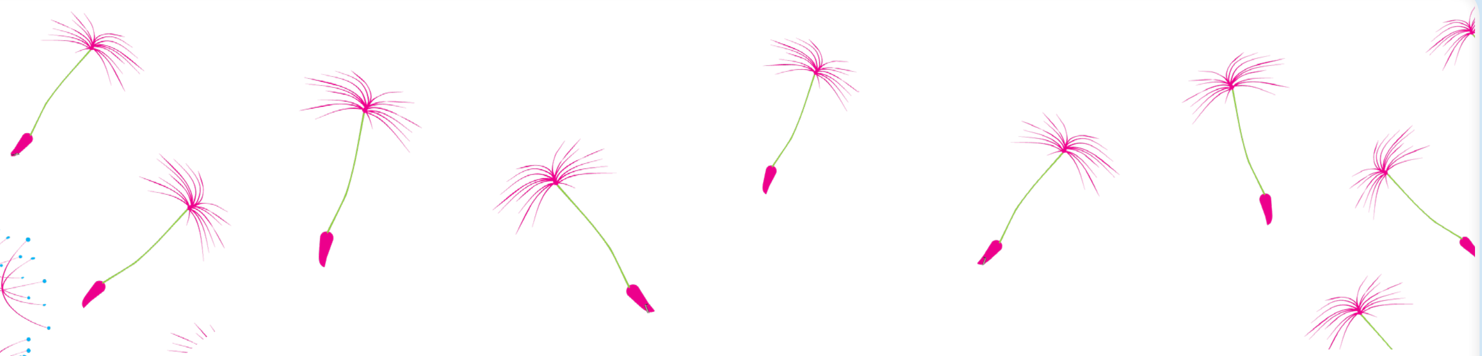

The next day, Agnese read all her manuals and books to build magical wings. She investigates them far and wide, from top to bottom.
She sets in motion her army of needles, threads and thimbles.
She tried it all. In vain ...
It's a big problem to help a girl fly like a feather!

Suddenly a place comes to her mind: the Garden of the Dandelions. It was there that the school dwellers made their wishes before Mr. Corona arrived, when the wind was still there and the words flew happily.
It is over there that the teacher must go. Agnese ran to the garden and grabbed a dandelion.

She closed her eyes tightly and expressed her wish: "I would like to sew a dress that can fly".
Then, she blew on the dandelion ... A light breeze colored her cheeks and stroked her hair: it was the Wind that rose, and took the dandelion away, far away. In its place it left her a magical light thread.
Agnese then understood that her wish had been fulfilled: "This is the hair of the wind! Here is the thread with which I will be able to saw the dress of the Butterfly Girl".

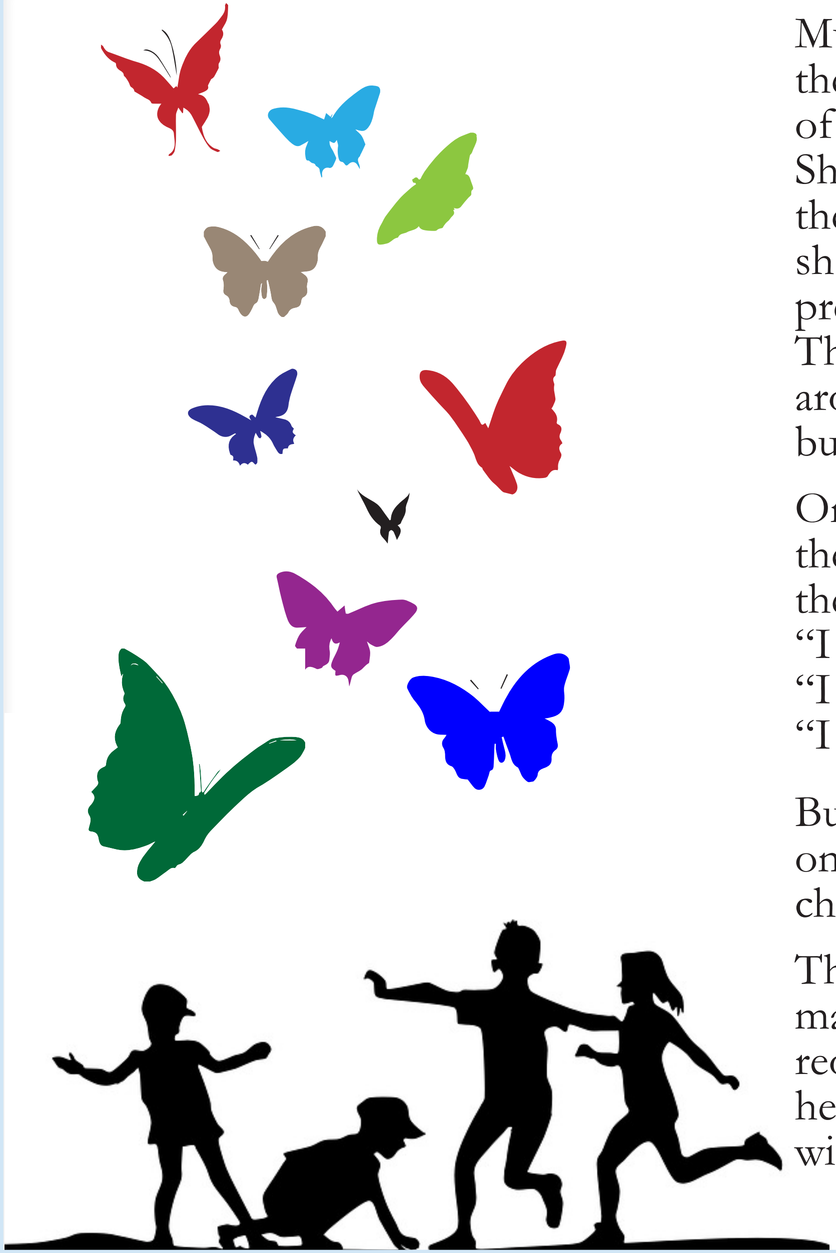

Much later inside her little house, Agnese exhausted, fell asleep on the big ball of yarn from which, during the night, some of the hair of the Wind escaped ...
She managed to keep her promise to the Butterfly Girl and created the dress she had always dreamed of. The Wind slips into the dress she has sewn all night and brings it to the Butterfly Girl as the most precious of gifts.
The girl was delighted with her wings to fly and flapped and fluttered around the town. And here, as if by magic, the good wind of the butterfly made Mr. Corona fly away forever!

Once free from the Coronavirus, mothers and fathers from all over the country opened all the windows and the dreams and wishes of all the children flew free in the sky:
“I want to discover a treasure!”
“I want to travel around the world!”
“I want to go back and eat the ice cream from Peppina!”

But the greatest wish, the one shared by all the children, was only one: to be able to return to school. Finally we can reopen! The wind chased away CoronaVirus!

The next day all the inhabitants of the school district gathered in the main square of the town to celebrate the return of the Wind and the reopening of the School. Many butterflies crossed the sky above their heads: they are their children’s wishes, dancing in the clouds on the wings of their dreams.

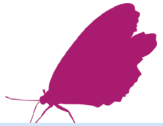

Educational material connected to the book "Welcoming children in the classroom after the coronavirus emergency" released under the Creative Common license BY-NC by ebookscuola.com

HANDOUT FOR ACTIVITY 7.2

Wash your hands!

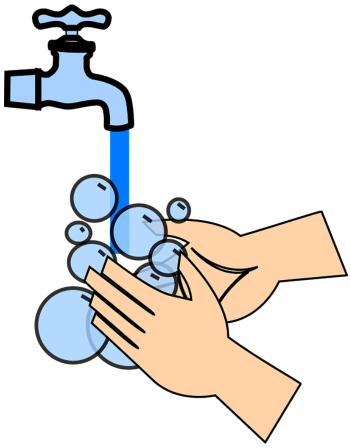

Educational material connected to the book "Welcoming children in the classroom after the coronavirus emergency" released under the Creative Common license BY-NC by ebookscuola.com

HANDOUT FOR ACTIVITY 7.3

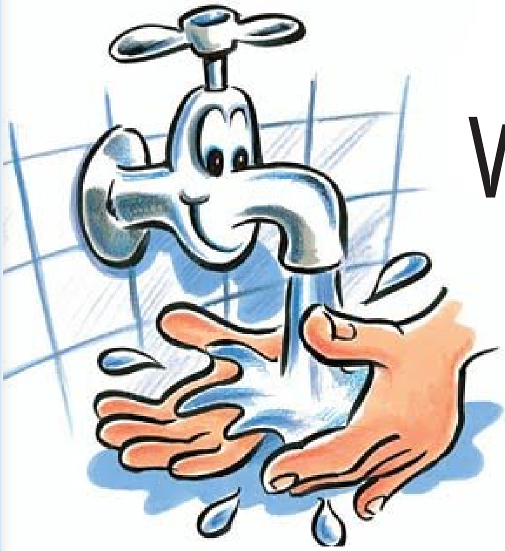

Wet

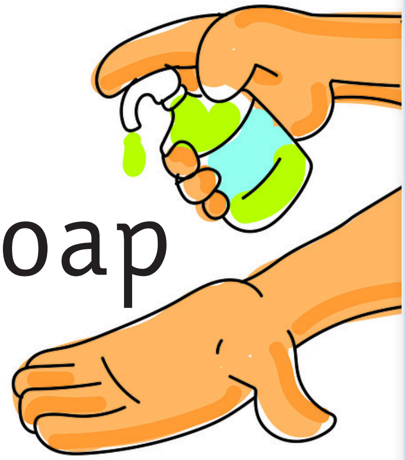

Soap

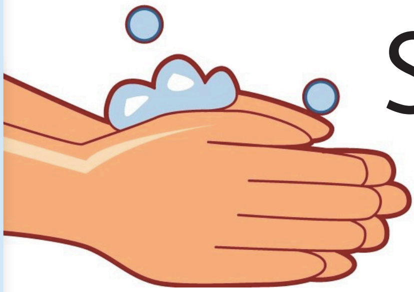

Scrub

Rinse

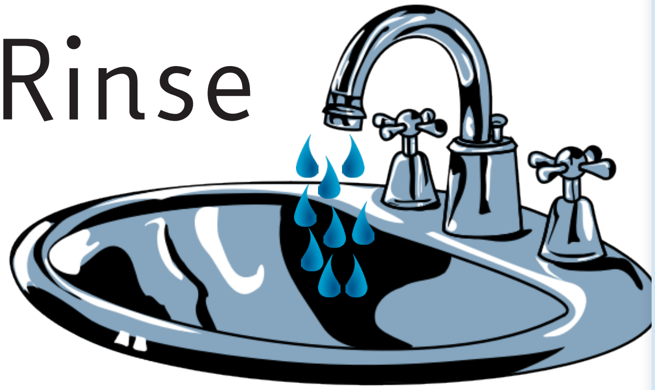

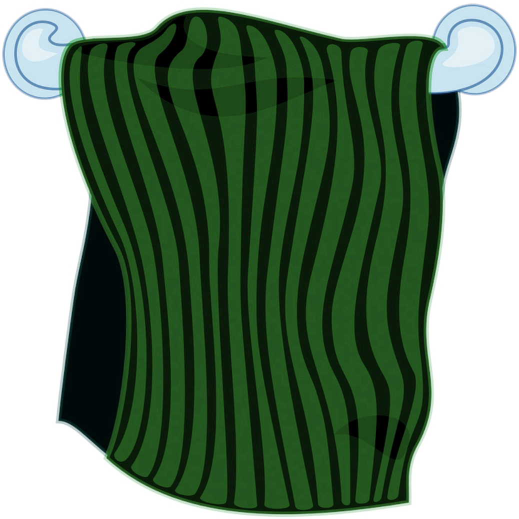

Dry

Educational material connected to the book "Welcoming children in the classroom after the coronavirus emergency" released under the Creative Common license BY-NC by ebookscuola.com
